# ﻿Jakob Emanuel Lange: The man and his mushrooms

**DOI:** 10.3897/mycokeys.89.79064

**Published:** 2022-04-26

**Authors:** Ronald H. Petersen, Henning Knudsen

**Affiliations:** 1 Ecology and Evolutionary Biology, University of Tennessee, Knoxville, Tennessee, USA University of Tennessee Knoxville United States of America; 2 Hauchsvej 15, 1825 Frederiksberg, Denmark unaffiliated Frederiksberg Denmark

**Keywords:** Biography, mycologist

## Abstract

Jakob Emanuel Lange (1864–1941), Danish mushroom taxonomist and illustrator, was an agricultural educator and economic philosopher. A follower and translator of the American Henry George, Lange was Headmaster of a “Small-holders High-School,” which served as a model for American folk-schools. Lange visited North America on three occasions. The first, in 1927, relied on his professional expertise; the second, in 1931, was purely mycological; and the third, 1939, was a combination of the two. All of this was lived against two World Wars and the Great Depression. This paper summarises the circumstances of Lange’s life against a background of the American mycologists of the day, the ominous events over his adult lifetime and his magnum opus, “Flora Agaricina Danica”, of five volumes illustrating ca. 1200 species on 200 coloured plates.

## ﻿Chapter 1. Introduction

The year was 1927. Henry Ford was wrapping up production of the “Tin Lizzy,” with 15 million on the road. With US population nudging 115 million, that was roughly one car for every 14 men, women and children, to say nothing of other companies and brands. The model T had leaf springs but no shock-absorbers and with few paved roads, automobile travel, like history, was a bumpy ride.

The United States was flexing its muscles. The era of Robber Barons had led to economic and financial strength. Names like John D. Rockefeller, Cornelius Vanderbilt, Henry Ford, Andrew Mellon and Andrew Carnegie resonate to the present day. Teddy Roosevelt had not only changed the view of the country regarding its “infinite” land holdings, but had taken on the monopolies and their lack of regulation. Railroads connected the coasts and carried the raw materials and finished products of the “Second Industrial Revolution.” The backbone of major industry, mining and manufacturing - factories, steel, logging and coal mining – was matched by budding technology, such as typewriters, cash registers and adding machines transforming how people worked. Ford had introduced the “assembly line”. The economic explosion included not only industrial growth and urban expansion, but also growth in agricultural technology, such as mechanical reapers. In a tiny corner of this juggernaut was the transfer of agricultural research in plant pathology into the hands of farmers (Petersen 2021). Mark Twain dubbed it “The Gilded Age”.

In a time of great expansion and fewer regulations surrounding wealth and business practices, circumstances were ripe for the rise of a class of extremely wealthy individuals who composed a very thin veneer of society. They wielded the power and means to create opportunities and jobs for the working class, but often with little regard for “workers’ rights” issues, such as discrimination, exploitation, workplace conditions and low wages marked the era. Interclass battle lines were being drawn.

In spite of this disparity and inequality, at least the United States did not have to deal with royalty. This was not so in Europe. For centuries, Europe’s social structure had revolved around the royals, the clergy and favoured mercantiles. The peasants remained poor and at the service of their local authority. Only the scent of the Enlightenment sought to provide some relief to the underclasses. Such movements, of course, were not supported by the ruling class and, while the cast of characters differed from that in America, the basic parameters of the social structure were similar. For Europe, the tableau played out in the 19^th^ century, for the United States, the 20^th^.

**Figure 1.**
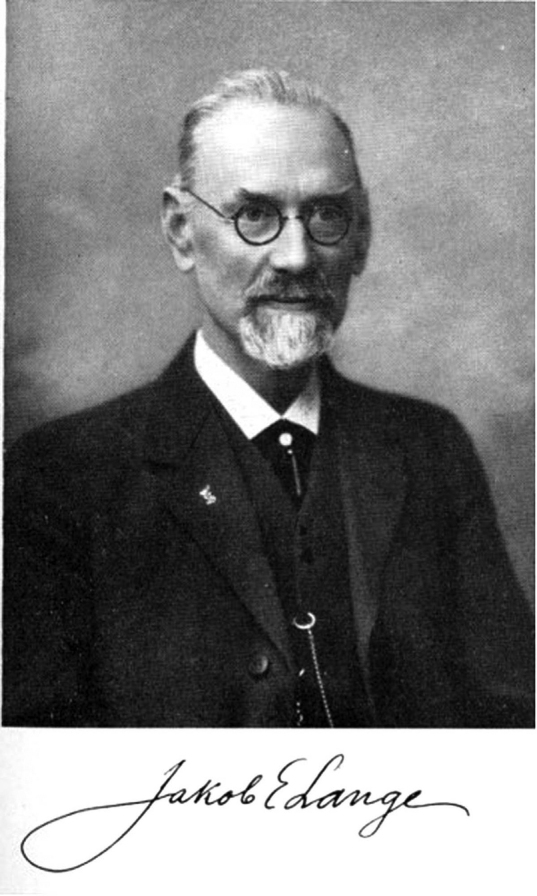
Jakob Emanuel Lange. Comparison of Lange’s signature reveals that it changed remarkably little over many years.

In the far-off Denmark of 1927, a modest farmer/teacher/politician named Jakob Emanuel Lange (2 April 1864–27 December 1941; Fig. 1) was already 63, in the prime of life. While Jakob Lange might not be a household name, he was to make his mark on not only the social equality movement in America, but the arcane world of mushroom taxonomy (Pearson 1946).

Born in 1864, Jakob Lange was a child of war. His father, M.T. Lange, was a vicar living in disputed territory between Germany and Denmark. His strong alter-interest was botany and, in 1858, he penned the first local flora, “The Flora of the Southern Funen Archipelago” (Lange 1858).

As a footnote to the turmoil in Europe during the mid-19^th^ century, in 1849, a new Danish constitution tempered the absolute rule of the Danish monarchs. In 1850, after a 2-year revolution, the southern provinces of Schleswig (a Danish duchy) and Holstein (a German duchy) seceded from Denmark and allied themselves with their German-speaking neighbour to the south, Prussia. A decade of turmoil ground on, pitting neighbours against neighbours, a conflict which would harden both Danish and German nationalism, profoundly instrumental in the Rev. Lange’s attitudes about life and education. In 1862, M.T. Lange became vicar in Angel [bordering Germany], but, because he did not want to work for the German cause, he was sacked by the Germans in 1864 and had to flee to Flensborg [further north, in Denmark] and there, during the escape from Germany, Jakob Emanuel Lange was born (Buchwald 1942). In 1864, the Prussian Germans took control of Schleswig-Holstein and it was ceded to Prussia in 1866, under the Treaty of Prague (Begtrup et al. 1926; Frommers 2021). Then, in 1866, another new Danish constitution was adopted, but it was more conservative than the one of 1849 and granted more power to those who paid the highest taxes, in other words, the landowners. Society was bound for re-stratification, not egalitarianism. It was in this era that Jakob Lange grew up. Henry George would have been disappointed.

As a young man, Jakob Lange became interested in botany, working as a gardener in high-profiled gardens and going to England (Kew) and Paris (Jardins des Plantes) for two years (1885–1887). Upon his return, he accepted a job as teacher at “Dalum Landbrugsskole” as a master gardener, although without formal education. The job was for four months, but he stayed 30 years. “It soon turned out that Lange had extraordinary abilities as a teacher; he could talk, he had no problems in visualising subjects and teaching was fun for him. His route in life was now on track and it was teaching/education in which he worked through the largest part of his life” (Buchwald 1942). At Dalum, he obtained his degree in 1887. His responsibilities included teaching young farmers botany, social economics and, interestingly, a bit of chemistry (Lange 1996). He also penned textbooks on botany (Lange 1897, 1916b) and physics (Lange 1899), each of which went through several editions.

**Figure 2.**
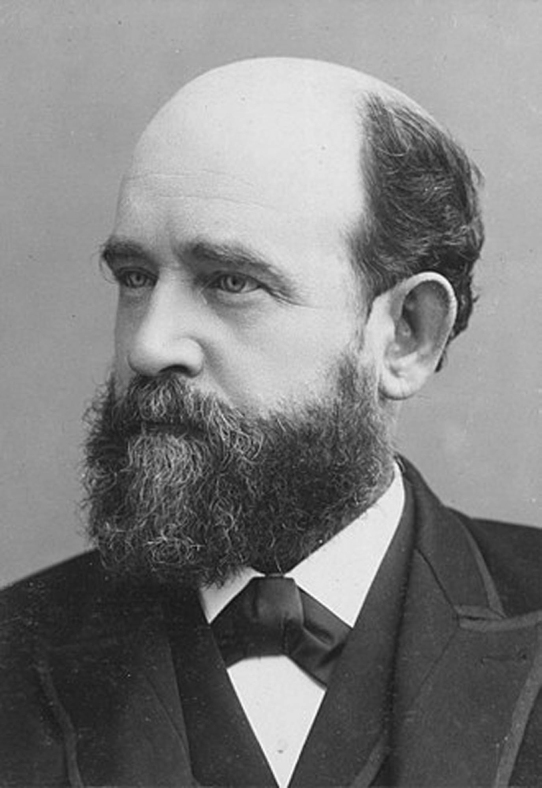
Henry George. Source: Wikipedia.

“During his time in England, Lange heard about Henry George^[1]^ (Fig. 2) and became acquainted with his major work, ‘Progress and Poverty’ (George 1879). In this way, his main interest in life, social economy, was started. From his first teaching year, he started giving lectures in social economy and the next year, he wrote his first textbook, ’Social Economy’ (Lange 1909), reprinted in a number of editions. In this way, it was Lange who introduced George’s thoughts to Denmark. Later, he made translations of some of Henry George’s books (Lange 1898, 1905, 1938c). His ideas about social economy were in focus when the so-called Land-law of 1919 was approved by the parliament, whereby ‘the peasants rights to the land, the people’s right to the land-interest’ was approved (Buchwald 1942).

“Already in his youth, through his father, [Jakob] Lange had received impressions about the popular and progressive thoughts about schools, a movement which grew in the middle of the 1800s. His father eagerly supported the small ... free schools which had been founded on Funen [a small island on Denmark’s east coast; also known as Fyn] in the 1860s and 70s, and through this, [Jakob] Lange early became aware of “small-holders’” social conditions. His attitude toward the popular context was further supported by being together with his father-in-law, free-school teacher Knud Larsen. In this way, he soon came in contact with the Danish small-holders movement and this led to his appointment as principal of “Fyns Husmandsskole”. In his leadership of this school, he enjoyed invaluable support from his wife, Mrs. Leila [Larsen] Lange (1884–1943), for whom the small-holders’ conditions were sensitive in her mind” (Buchwald 1942).

As adjunct to his participation (and subsequent teaching position) at the Dalum school, Jakob Lange also became prominent in social and political movements, over time becoming a leader of the Danish Social Liberal Party [now Det Radikale Venstre]. In 1914, he was asked to assume the superintendence of the “Husmandsskole” (“Small-holders’ school”) in Odense near Dalum on the Island of Fyn (aka Funen). He refused, professing that he was comfortable at the Agricultural Folk High-school, but the invitation was renewed in 1917. “The Great War” was at its height, Denmark was neutral and this time he accepted. Buchwald (1942): “It did not take many years before Lange threw himself into the study of the fungi, the exploration of which would become his real speciality, i.e. the difficult group, the agarics. One of the difficulties in this study is that their occurrence is restricted to a few months in the autumn. However, these conditions were in his favour, because the agricultural schools were closed in those months so the students could go home and help with the harvest – and this gave him a unique opportunity to study these fungi”.

Jakob Lange was multidimensional (Lange 1996): his pedagogical and political lives filled his days, but a growing fascination for the local mushrooms of Fyn, had, from its beginnings in the 1890s, bred a small library, growing knowledge and a cache of water-colour illustrations that were becoming known and appreciated by the Danish mycological community. Of Lange’s three trips to the United States, the first was borne of his professional occupations, the second by mushrooms and the third by both. Indeed, the impetus of his 1927 transatlantic travel had little to do with mushrooms, but everything to do with the common cause for the dignity of the working class.

Jakob’s first invitation to visit the United States had come in 1914. The fateful assassination in June of a second echelon royal lanced the abscess of ill-will in Europe and before the summer’s end, war was spreading. For America, “The Great War” (World War I), as it expanded across Europe, seemed distant. As Lange (1938b) put it, “In 1914, just after the war had started, I received an invitation from America to talk about the Danish situation, at the American teacher-organisation’s annual meeting in San Francisco, 1915. ’It is expected you can speak an understandable English, you will be given 25 min. and a salary is not given for principle reasons.” It was added, in a modicum of good will, that the host would arrange a paid tour of lectures in connection with the meeting. “It was flat out exciting; and I also answered yes, under one condition: that the war at that time [1915] would not be close to Denmark – and then, of course, the plan was cancelled and any thoughts of a travel to US were also cancelled”. Although Denmark remained legally and politically neutral, transatlantic ship traffic was insecure. The cancellation was followed by a 13-year hiatus for hopes for a U.S. visit.

**Figure 3.**
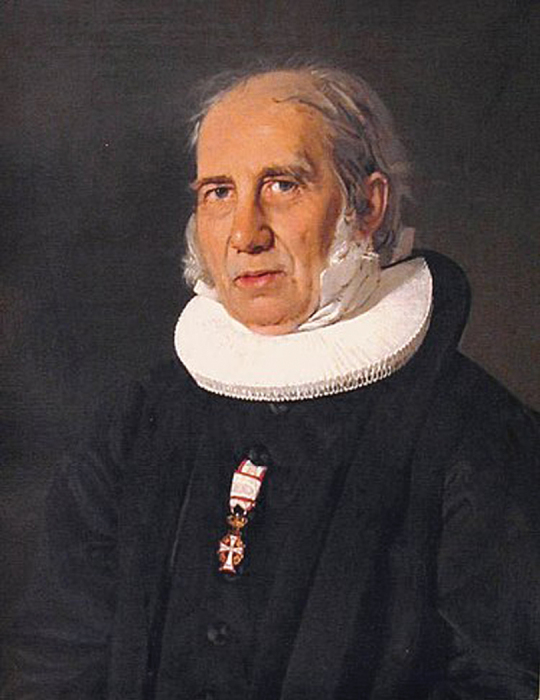
Nickolaj Frederik Severin Grundtvig. Source: Wikipedia.

However, what was “the Danish situation” about which he was to speak to the American teachers organisation? Surely not Danish In the mid-1800s sketched above, there was little movement towards equality of society by the peasantry. It was only from 1788 that farmers were freed of their attachment to the nobility, i.e. they could now live wherever they wanted. Against this grain, Nikolaj Frederik Severin Grundtvig (1783–1872; Fig. 3), a Lutheran cleric born of the upper-class, spoke of a peasantry with power within the greater population. Studies at Trinity College, London, in 1831, 1832 and 1833, helped to mature his thoughts and he began to develop the idea of a kind of folk school particularly for those who could not afford formal education, producing a peasantry ready to take its place in Danish culture and governance. His mantra was “First a Dane, then a Christian”. His followers celebrated nature through Danish language, song and history and became known as “the Happy Danes.” Using his pulpit as Lutheran pastor, his sermons reached growing congregations and, in 1844, the first “Grundtvigian high-school” was founded. Within a few years, the 1849 Constitution granted some aliquot of governance to the people, the Grundtvig movement quickly grew and, by the end of the 19^th^ century, several hundreds of such schools had been formed, some reputable, others not so. Both the Agricultural Folk High-school at which Jakob Lange taught for 30 years and the “Husmandsskole” in Odense, to which he moved, were of the Grundtvigian tradition.

**Figure 4.**
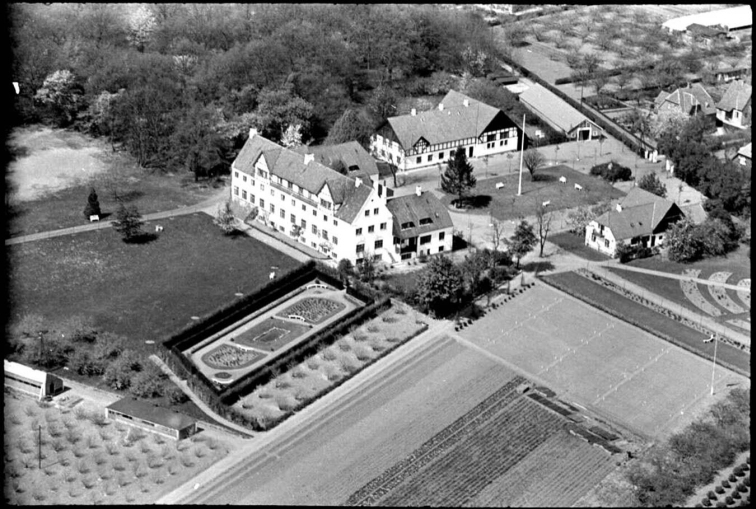
The Odense ‘husmundsskole’, probably pre-WW II. Major building and ancillary buildings. The practising farm is at lower right, the orchard is top right and lower left, the formal garden surrounded by hedge is central. Source, DKArkiv.

**Figure 5.**
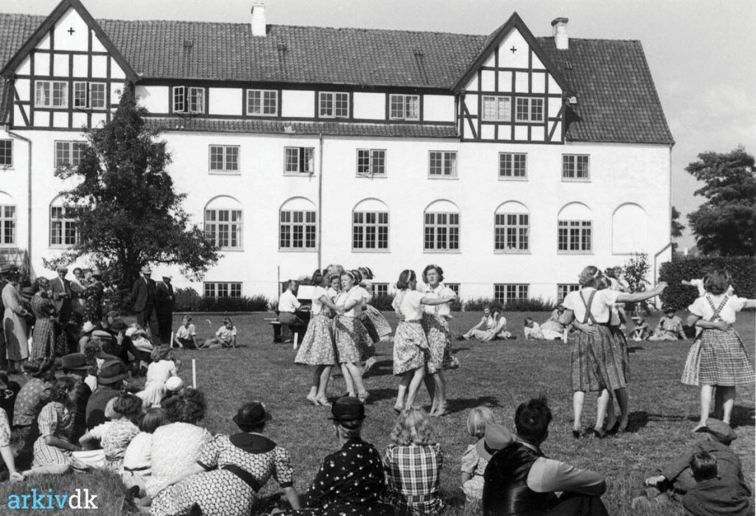
The Odense ‘husmundsskole’. 1940. Source: DKArkiv.

**Figure 6.**
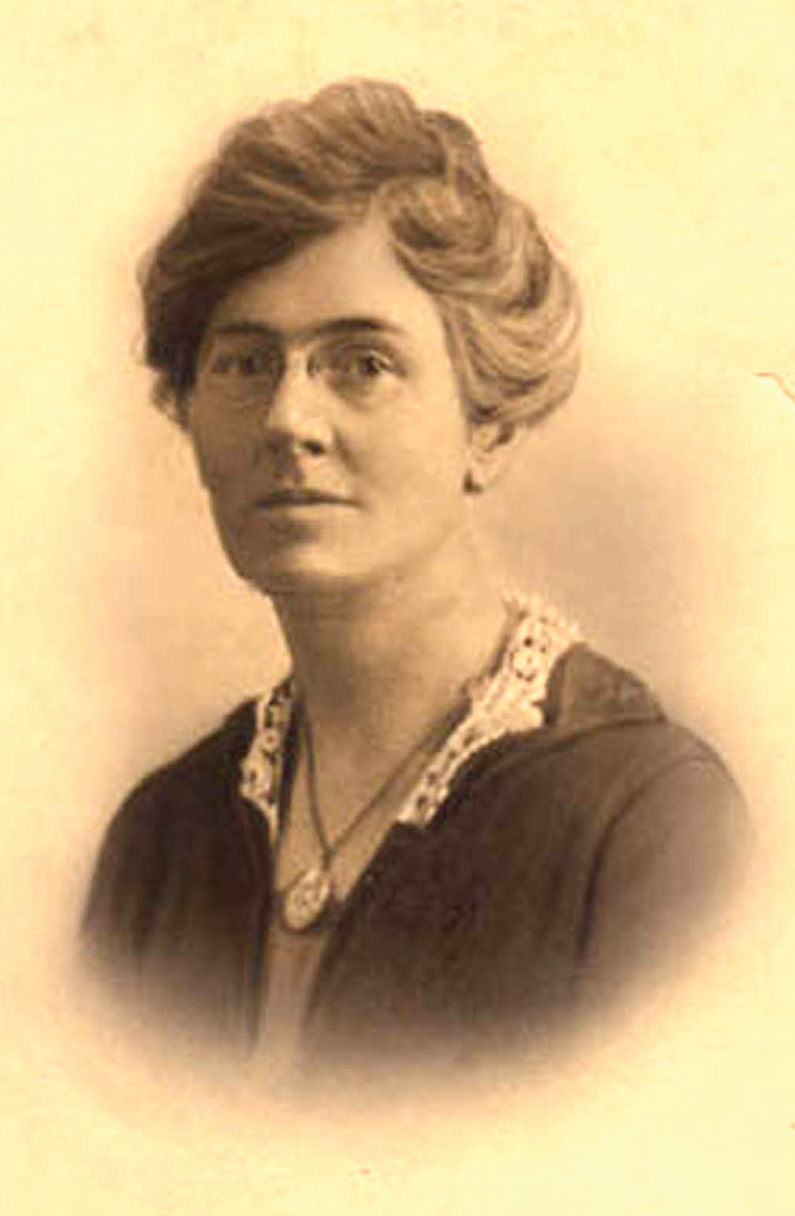
Olive Dame Campbell. Source: Wikipedia.

**Figure 7.**
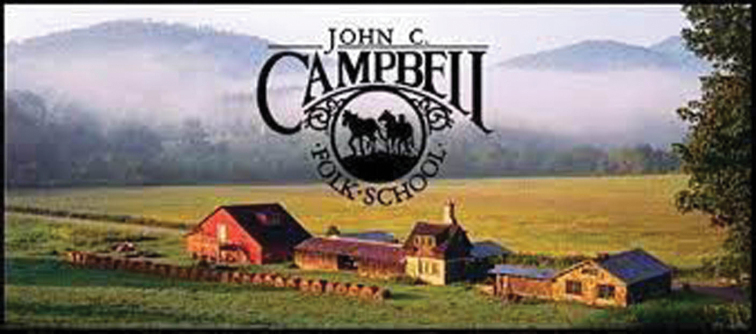
John C. Campbell Folkschool advertising design. Source: John C. Campbell Folkschool.

Although founded before Lange’s leadership, the Odense Folk High-school (Figs 4, 5) had become reputable even beyond Scandinavia. As part of its popularity, the school played host to frequent visitors, some of whom were advocates from the United States. Lange (1938b): “One of the most important Americans who visited the school, was Dr [Philander Priestly] Claxton [1862–1957], in his time ’commissioner of education’ [i.e. commensurate to today’s Secretary of Education], which modest title can be better translated as ’general director for education in the United States’. Claxton was very interested in the conditions in Danish agriculture, especially because he encountered a trend which did not (without fighting) accept the minority feeling (a feeling of less- worth) which the COUNTRY-CULTURE almost all over the world has against CITY-CULTURE, not least in America. Both the [Danish folk] high-school and the ordinary village-school, with their independent teacher-families, were ideal to him. He was only too used to seeing the American country district teacher, more often female, always turning their face towards the nearest city, longing for advancement to a city-school, just as, on a dark winter night on a deserted road, you would be longing towards the cultural centre you may faintly see on the horizon over Svendborg or Bogense [two local Danish cities].” Claxton was a Tennessee native, born in a log cabin and an evangelist for state funding of rural schools. Lange: ”“The one among all our guests, who developed the deepest appreciation of the Danish high-school system and thoughts about schools, was, however, Mrs. [Olive Dame] Campbell^[2]^ (Fig. 6). She visited first the high-schools in Scandinavia and Finland, where she found a way of organising school work, she preferred to take to the ’small farmer’ society of poor mountain farmers in the Allegheny Mts. [sic], which she for her whole life had been interested in to work among and for. The book she later wrote [Campbell 1928] about Nordic high-schools is undoubtedly the most intimate understanding written about this subject and the ‘small farmer’ school she founded in North Carolina [John C. Campbell Folk School in Brasstown, North Carolina; Fig. 7] is in a strange way a living ‘work,’ which in various ways serves the ‘propagation’ of the ‘Mountain peoples’ public and socio-economic ‘liberation’ and prosperity. Above the entrance door to this school is a bell, given to her by Danish high-school people” (Campbell 1929). On the other side of the mountains, in Tennessee, Horace Kephart was revealing the life of southern mountaineers in more whimsical prose (Kephart 1913).

**Figure 8.**
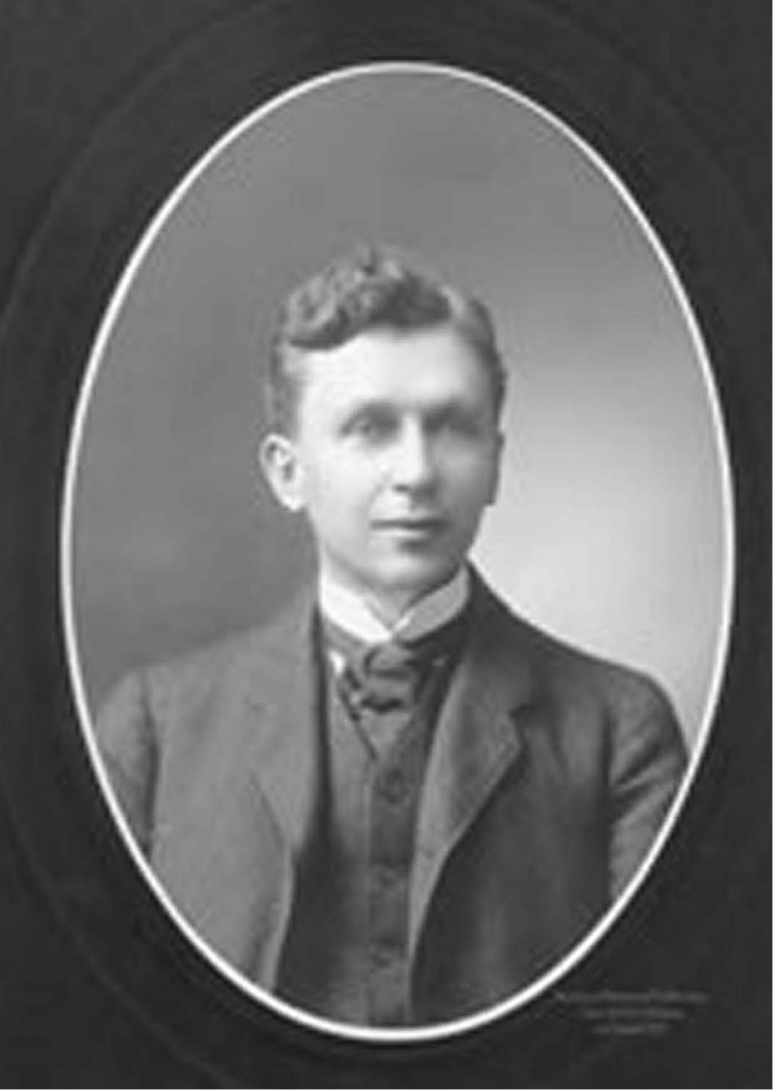
John Charles Campbell. Source: Wikipedia.

Widowed in 1919, Olive Dame Campbell (1882–1954) had carried on the work of her husband, John Charles Campbell^[3]^ (Fig. 8), surveying and measuring the rural cultures of the southern Appalachian Mountains and conjuring what could be developed to bring education and dignity to those isolated populations (Campbell 1921, 2018). She and her friend, Margarite Butler of rural Kentucky, were each awarded fellowships from the Russell Sage and the American Scandinavian Foundations to assess the Scandinavian Folk High-School movement, curricula and ambiance.

**Figure 9.**
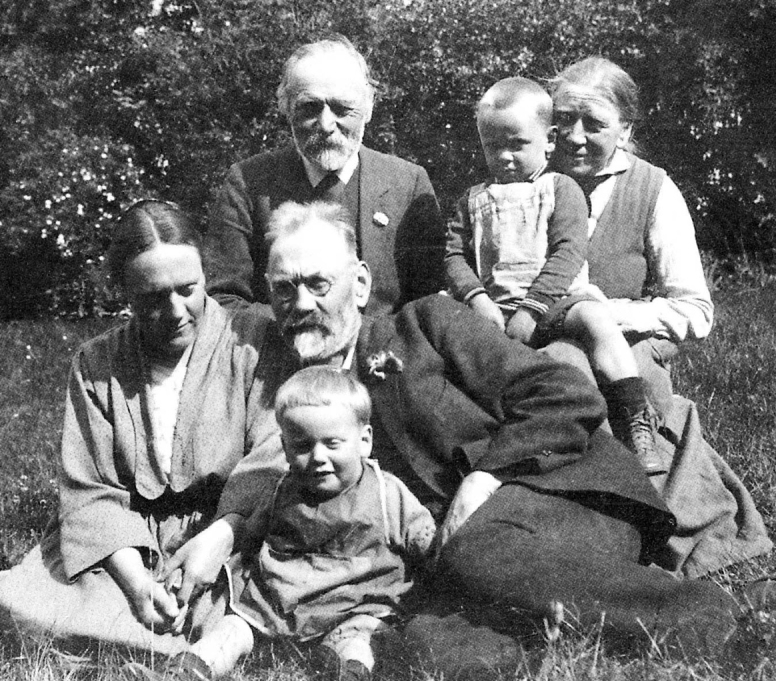
The Lange family. Left: Leila Lange; Centre; top, Leila’s father; centre, Jakob Lange; lower, Jens Jakob Lange; Upper right, Knud Morten Lange (age perhaps 2), Johanne Lange.

**Figure 10.**
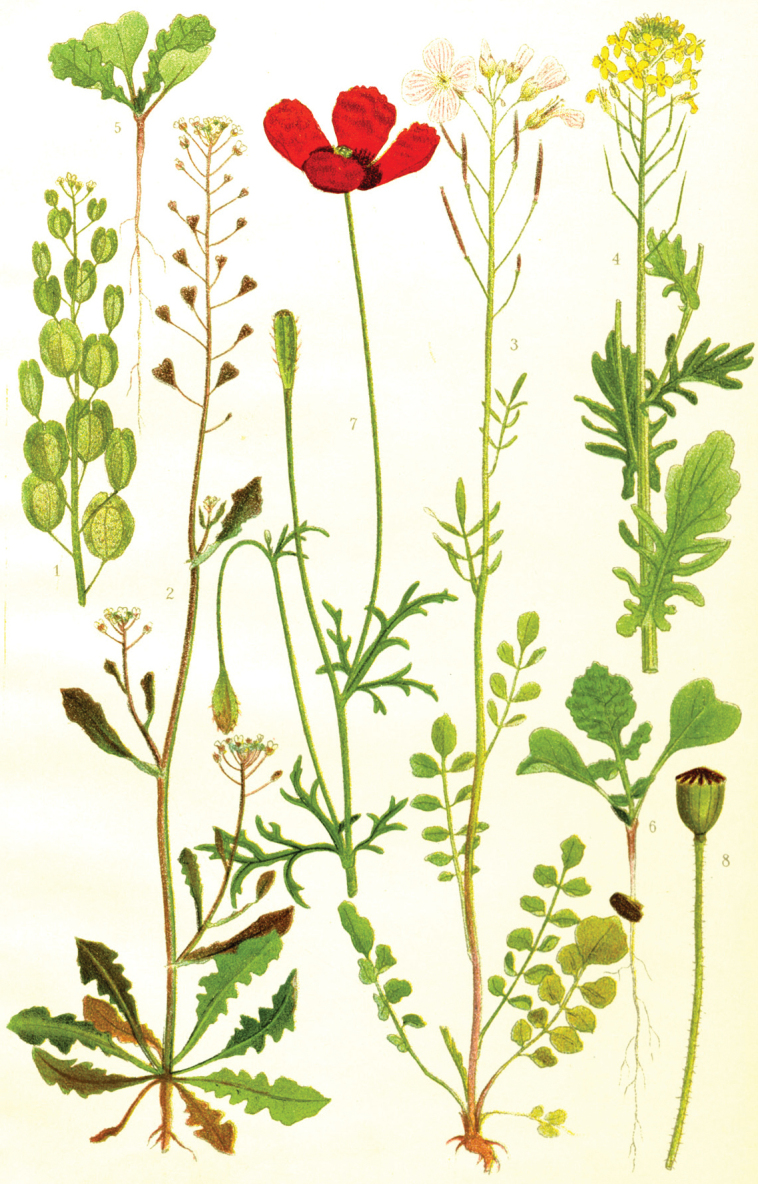
Wildflower painting by Jakob or Johanme Lange. Source: Natural History Museum of Denmark, Copenhagen.

Lange’s sincerity of feelings was shared by Mrs. Campbell (1928): “The relationship of equals was certainly well maintained at Odense. Possibly the fact that the students were older and came with a serious purpose had something to do with the atmosphere of comradeship. I recall always my impression of this on my first visit, early in my stay in Denmark. When Mr. and Mrs. Lange [Fig. 9] led us down to the dining room and found us a seat on the long benches, something ‘folkeligt’ as we say in Denmark, was to be sensed in the atmosphere – something which may have been due to equal ages, small numbers, simple food, lack of convention, or especially to Mr. Lange’s convictions and Mrs. Lange’s very warm and natural personality. Conversation was carried on freely, often across tables and even across the room. We had, I remember, barley porridge with butter and sugar, cold ham and carrots stewed in a sauce, rye bread and a pink fruit drink … When Mr. Lange rose with ‘*Velbekomme*,’ our ‘*Tak for mad*’ (thank you for the meal) was such as we would have given in any country home”. According to school lore, the walls were decorated by paintings of local wildflowers, mostly by Jakob (Fig. 10).

Campbell (1928): “The aim of a Grundtvigian high school was to help common people qualify as active and engaged members of society, to give them a movement and the means to change the political situation from below and to be a place to meet across social strata. This atmosphere would materialize in the collegial atmosphere and mutual respect between the teaching staff and the students who would all live together in a small community transcending social classes. The egalitarian atmosphere at the schools was important. By singing, reading and exploring their surroundings, the students at a folk high school were also meant to have a joyful and uplifting time, not only to obtain educational knowledge (Warren 2012, 2021). Examinations were prohibited”.

Democratic (with small “d”) principles permeated the Odense school. “Mr. Lange told us that a committee of seven met yearly, when necessary and could dismiss the principal on six-month’s notice. Five of the committee members were from Fyn, two being selected by former students. The chairmen of the organisations of Sjaelland and Jylland are *ex officio* members of the committee, but did not take an active part in the management. Mr. and Mrs. Lange got the same salary and board regardless of whether the school was prosperous or not, an arrangement which left them much freer than as if they had the burden of private ownership. They were not likely to be dismissed or interfered with unless they should become violent reactionaries in politics or radical opponents of religion, in which case ‘we would ourselves undoubtedly wish to leave’. … A combined cultural and practical program calls for a skilled and expensive staff”.

[Experts from the Agricultural College taught at the Folk High-School in the winter, when they had spare time from regular duties during the growing seasons.] “The *Forstander* (Superintendent) can engage whom he pleases. … The school also owns a large farm, run solely for school support although on demonstration lines. It is in the charge of one of the old students, a trained farmer. Each year, five new graduates are given the much-prized opportunity of working there for the practical training. They are permitted to remain only one year, with the exception of the cattleman who stays longer.

[The school] “stood firmly on the two essentials, family life and the relationships of equals. … The relationship of equals was certainly well-maintained at Odense”. Campbell (1928): “The men’s course of five months takes up geography, history, literature, life of the peasantry since 1788, botany, physics, a very little chemistry, social economics, animal husbandry, anatomy, accounts and bookkeeping, gardening, plant cultivation, soils and manures, with occasional other lectures. Gymnastics is also given. The manner of teaching is much the same as in the regular folk school”. A five-month course is offered to women as well as to men although about one-third leave 1 August, after three months. Besides the usual cultural subjects, they receive cooking and sewing lessons. In addition, each woman is given a small garden plot where she raises both flowers and vegetables. Two periods of one and one-half hours a week are devoted to this work under the direction of the horticulturist. The weekly schedule shows not only subject matter, but Lange’s responsibilities in the classroom.

**Figure 11.**
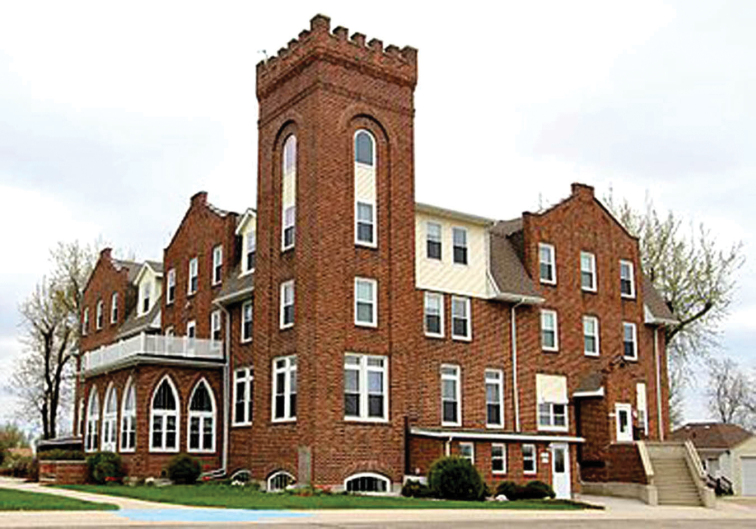
Danebod Folkschool building. Source: Danebod.com.

The durability of the Grundtvigian folk school is testified by Danebod, a small neighbourhood in Tyler, Minnesota. This tiny Danish enclave was settled in 1885 and, almost immediately, a three-storey brick building for the school was built. For more, see bibliography under Danebod (2021a, b; Fig. 11).

In 1924, the Social Democrats became the largest party in Denmark and took the reins of government. The large Left Party (i.e. the liberals) suffered internal strife resulting in a division between the larger group, which adopted a moderate liberal attitude and a small group whose direction inclined to a more radical liberalism. In both parties, the rural population was the fulcrum, but farmers and small-holders were found in both (Hollman 1910; Begtrup et al. 1926). It was in this more radical wing that Lange was to be found.

So to the question of “the Danish situation” over which Lange was to expostulate in the United States in 1915 (the invitation of 1914): it could have been political parties in Danish politics, the Grundtvigian folk-school movement, agricultural education in Denmark or Danish professed neutrality in the growing war in Europe, in all of which Lange had more than passing expertise.

## ﻿Chapter 2. 1927

**Figure 12.**
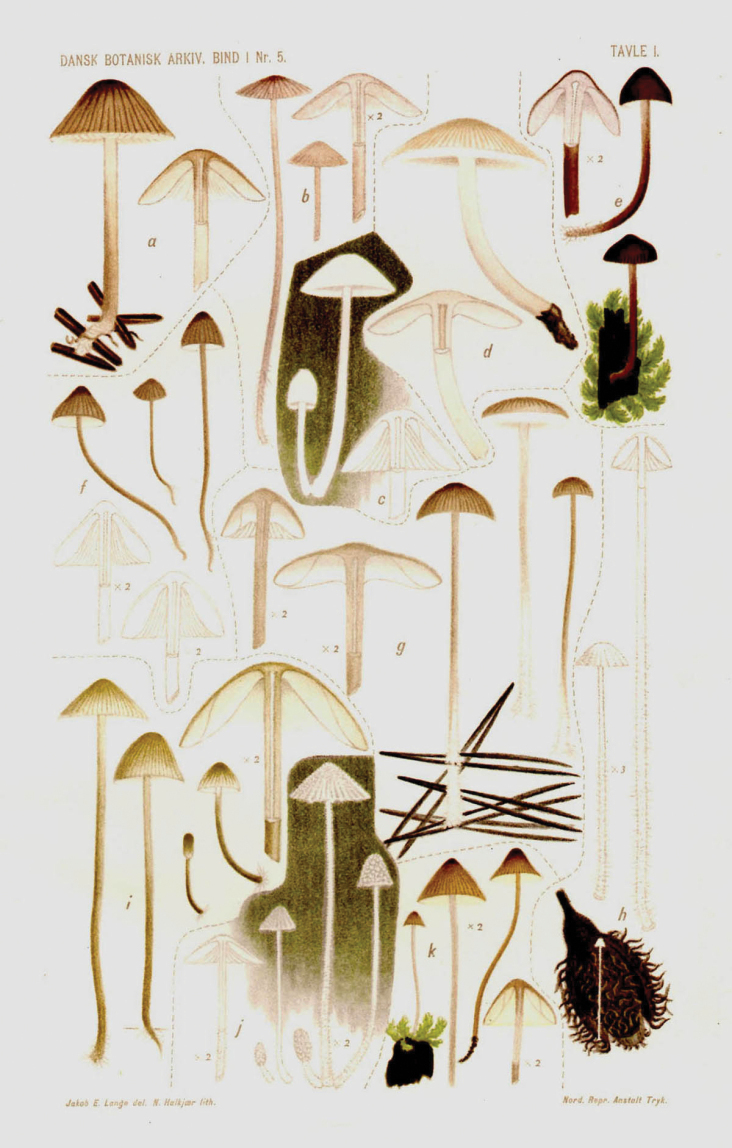
Plate of *Mycena* taxa from first instalment of Lange’s “Studies.” Source: Dansk Botanisk Arkiv.

In 1914, the same year as the first invitation to visit the United States, Lange began a series of published studies on the mushrooms of Denmark – with text in “understandable” English! – sometimes accompanied by montages of his own watercolours of the species involved (Fig. 12). He wrote that he had started creating the images in 1893 and, by 1914, numbered 622 (they would eventually climb to over 1200). He contemplated that they would supplement his “Afbildninger af Danmarks Agaricaceer” (“Illustrations of the Agarics of Denmark;” Lange 1914), Lange’s name for his collection of aquarelles, later to become “Flora Agaricina Danica” (Lange 1969). The “Studies” series, 12 in all, extended from 1914 through 1938, three years before his death Lange (1914, 1915, 1917, 1921, 1923, 1926, 1928, 1930, 1933, 1935b, 1936a, 1938a). Already in 1888, Lange had written on potato-blight on tomatoes and his interest for this practical use of mycology continued in his pamphlet on common plant pathogenic fungi (Lange 1916), not published in some abstruse journal, but independently, so it could be distributed directly to the nearby farmers.

Two principles undergirded the “Studies” project. First, a thorough study of the agarics of Denmark had not yet been accomplished and no one was more familiar with them than Lange. Accordingly, the series furnished keys and descriptions and, where necessary, illustrations, including occasional coloured plates. Second, with the polyglot of language across Europe and Danish certainly minor amongst them and with the larger mycological community of Great Britain and the United States sharing a single language, English was preferable.

Lange was already obsessed by the notion of “agaric richness.” Starting with the first in the series, Lange’s own tally of Danish species numbers was compared to counts of Fries’ species of the same genus. Lange (1914) was always impressed that, in such a small area as the Island of Fyn (“barely 40 km in both ways”), he could produce such large numbers of species – comparable to those logged by Fries, with the exception of the Scandinavian conifer fungi. He was to expand further on his ideas after his 1931 American foray (Lange 1931, 1934).

Lange (1938b): “... in 1927, the [1914] invitation was repeated in a new form. We had at the high school eventually received many connections in socially and pedagogically interested circles abroad. And now came a request to me to talk at an international congress in Lansing (Michigan), called for by the American department of an organisation, which in English is called ’Country Life Association’ and whose purpose was all over the world to find out and speculate in what could be done to improve life conditions in the countryside, economically, socially and by raising the information level. And furthermore, I received an invitation to talk at the old Univ[ersity] in Williamstown in Massachusetts [Williams College] at a meeting there, also of international contents, organized by a society calling themselves ’Institute of Politics,’ really a national-economical society for information. The payment at these meetings was so ‘American’ [considerably more charitable than European] ... that the travel costs could be met. And by a row of other connected lectures (for a more European payment), it was made possible for me to bring my wife too, which became especially interesting to me since it was arranged that our travel could be extended all the way down to North Carolina, where we could be present at the inauguration of the new assembly building Mrs. Campbell had established as the centre for her new high-school”. Neither Knud Morten Lange (24 November 1919 – 10 November 2003; in 1927, eight years old and known to everyone as Morten), nor Jens Jakob Lange, their other son, were mentioned and assumedly remained at home, fully attended by the students and staff of the school.

Lange^[4]^ (1931): “It was an extremely interesting travel, very different from the American travels other [Danish] high-school men had taken in preceding years. For them, the main purpose was to visit the Danish small villages and religious circles, especially in ‘the Middle West,’ the States to all sides with Chicago as the centre. My travel on the contrary went through the old States, the East coast, from Maine to North Carolina and only at one point to the west, passing Niagara to Lansing in Michigan. And we did not see many Danish. On the contrary, we received a much varied and quite in-depth impression of the Americans, far more comprehensive than the Danish high-school people normally got, even after many years in the US.

“The ’Aquitania’ was one of the largest swimming monsters of the time, with seven decks. But a sailing trip across ’The Pond’ was anyway not very exciting: remarkably few ships are met with on the route. Days may go by without seeing any. And then when you finally reach the harbour in New York: passing the Statue of Liberty, the light of which, according to malicious tongues, is only shining outwards, not over America, the symbol of which she should be.” This observation on societal inequality, among other experiences, was a reaction to the United States’ Johnson-Reed Act of 1924, known as “The Immigration Act”. It restricted immigration to 2% of the number of nation-immigrants from each country, measured by the census of 1890. Asians were prohibited totally.

“After Medical Control [on Ellis Island, the reception centre for immigrants for decades] and other modern plagues, we finally came to the city and saw the ’skyscrapers’ of Manhattan in front of us. The strange thing about these is that it was a BEAUTIFUL view. The reason is probably their sky-striving character (the one they share with the old Cathedrals, with which they have a certain resemblance in my memory). It is almost as if all the Cathedrals of the World have decided to meet here. And add to this their functionalistic appearance, a feeling of ’problem solved’- mathematically correctly. In this way, it is possible, under narrow area conditions on stony ground, to build a business town for a million population. But it was strange, when you walk around in the narrow streets, which felt like the bottom of huge canyons, to see amongst them an old church, with a tower which was impressive for its time but now looked like a small, sleepy candle-snuffer, hardly reaching 1/3 towards the top of the skyscrapers from its small green churchyard in the centre of a large city’s stone desert”.

“The congress in Lansing, the capital of Michigan, took place in beautiful surroundings, at this state’s agricultural high-school [later renamed as Michigan State University]. Everywhere in America, universities – and even smaller schools – are surrounded by large parks with very old trees of beech, elm and maple and the buildings are usually large, wonderful and dominating.

“One of my best days was when I spoke to a thousand-fold audience of farmers from the area. It was almost like being home at the agricultural school to a camp – only that here there were loudspeakers and other devices at that time unknown in Denmark and a huge orchestra of about 50 musicians. ... We also had curious experiences, like at a ’Rotary’ lunch, which was very American and took place in a large hotel in Lansing. My wife and I were, of course, placed at the head table with the other guests of honour: to the left the beauty queen of Michigan (with her mother and fiancé), to the right ‘the world champion mouth-organ player!’ I had the triumph that I, although handicapped in the competition, could spell-bind the busy businessmen, who here had a short schedule, by my story about the Danish cooperative movement– for even more than 20 min, which was said to be a “record”.

“At the meeting/camp in Williamstown [Williams College, Massachusetts], the old conservative university in Massachusetts (where female students were not allowed), the atmosphere was somewhat different, more upper class. It was very international. A dominant role was taken by the young H. A. Wallace^[5]^ – now [written in 1938] known everywhere as Roosevelt’s secretary of agriculture and legislative active administrator. The British foreign-press chief talked (about England’s economical policy in China); a former German Reich’s finance minister shed light on Germany’s economic policy after the war [World War I, reparations] etc. And here I was to talk about something so small in a world context as Danish farmers. Was it likely to be of any interest? – It was a hall the size of the Copenhagen cathedral, but luckily there was a loudspeaker – and [the talk] was OK; at least the Italian Count Sforza hurried up to my wife after the lecture and kissed her hand as a thank you: what else can you ask for? By the way, they had changed the title of my lecture. I had called it ‘Progressive Peasantry’ (farmers in an era of progress); but it was changed to ’Agricultural Progress’. Why, I asked? And received the answer that ’peasant population’ and ’progress’ were mutually exclusive - two items which could not be thought of as being related. That gave me a lot to think about.

“I shall not count the different agricultural high-schools and universities we visited during the tour. When we had the laborious tour behind us, we could with a good conscience go to Mrs. Campbell in ’the mountains’.

“While most of the villages in the southern states are ’Black,’ there is a population in the Allegheny Mts. valleys which is, for America, an unusually clean and pure English-Scottish race. These mountain people constitute a ’tribe’ of their own. They are descendants from an early immigration to the North Carolina lowlands, but were later forced by mass immigration from Europe into the valleys in the mountains, where they settled, whereas the other immigrants continued across the [mountains] and into Mississippi. Now they constitute a peculiar farmer-people, extremely old-fashioned, speaking practically what you would call an old-English dialect, living from pieces of old poems, homemade, traditional art-work etc., but now [find themselves] in [a general] explosion and dissolution, along with the arrival of modern central schools: highways form a net of modern culture into the valleys of the theretofore almost roadless valleys’ quiet life.

“It was among these farmers and small farmers that Mrs. Campbell saw that the high-school had a mission, as already mentioned. Now she had come so far that her own agricultural farm was not alone in exemplary order, but she had founded a co-operative dairy, organized co-operative buying etc. and also in a modest way started the school. And now finally the first real house, the assembly hall made of timber-logs was finished and it was for this celebration we were invited. A lovely room, with mat brown waterboards on the walls and homemade chairs instead of benches, one from each home in the village.

“It was curious to see the population coming from near and far in their delicate [horse-drawn] chariots with picnic-baskets, babies etc, almost as you may imagine the [Danish] ’sky-mountains parties’ (the highest point in DK is 189 m) in the old days of Blicher [a Danish poet depicting country life]. The speaker following me was President Hutchins from the Peoples’ University of Berea^[6]^ in neighbouring Kentucky (Berea 2021). And to the population, as for us, this celebration was a strange event, which established that now, with the new enlightenment, co-operative efforts had resulted in something real. To us, as Danish, it had the strongest effect when a small choir of the youngsters in the school sang for us in Danish a few verses of ’I am a simple farmer’ to greet us and as an expression of the living connection to the Danish high-school and farmer-culture. ’I sing behind the plough’ was selected as the motto for the school and is written on their emblem.

“From North Carolina, we headed for New York. Before we sailed home, we had the opportunity to participate in a Henry George gathering. It was interesting to meet men, whose names were well known to me from the information and agitation work of George’s ideas through many years. The person I had the most living impression of was Hamlin Garland, whose collections of short stories (“Main Travelled Roads” from the 1890s) was the first who gave a ’living picture’, based on a realistic social background, of the American country population’s life and thinking. The most direct connection I had, however, was by meeting Mrs. Anne George de Mille, the youngest daughter of Henry George, who is fully occupied with the continuation of her great father’s ideas among the people of our day. At the congress we, by the way, had the impression that this was needed. The audience was mostly old people, but fortunately Mrs. de Mille and other younger people’s efforts now seem to be fruitful among the younger people.

“The most important of the veterans from Henry George’s own time, Louis F. Post^[7]^ and his wife Alice Thatcher Post were not present at the [Lansing] congress, but we got a very living impression of them in their home in Washington [DC]. They are now very old but fully engaged by all the important questions of our time. I made them happy by bringing my thanks for their weekly journal ’The Public’ which through many years meant a lot to me by its progressive and bold talk”.

Almost as an after-thought, Lange mentioned a couple of mycological oddities he saw in the wilds of far-western North Carolina. The first (Lange 1934) was reported within a discussion of mycological “parallelism,” a subject expanded in a subsequent chapter of this paper. Briefly, Lange suggested that many American mushroom species resembled European agarics so closely that they were (in his terminology) “parallel”.

“One of the most peculiar cases of parallelism is the existence in America of a phosphorescent form of *Panusstipticus*. While there is no record, as far as I know, of any phosphorescence in the European form, the American one is renowned for its bright noctilucency [sic]. I myself encountered this phosphorescent form in North Carolina (Cherokee Co.) in 1927. When seen by daylight the specimens were exactly like those so commonly collected in Denmark. Whether the phosphorescence is a real specific difference or can be accounted for by atmospheric conditions (or bacteria) remains to be decided by further investigations”. The local folks were familiar with luminescence, which was known as “foxfire.” Had Lange been present later in the year, he might have seen the “Jack O’lantern” mushroom, *Omphalotusilludens*, much more impressive than foxfire.

Lange (1931): “In North Carolina, I found a very peculiar fungus, a short-stemmed gastromycete, with an outer membrane which is very ephemeral, like grainy clay or moduling cork, which, when it disappears, reveals a smooth, egg-like pale grey-brown ‘sporehouse’, the opening of which is decorated with a star-shaped, shining cinnabar red mouth, which very aptly has been given the common name, “beauty-mouth,” most likely *Calostoma* [*lutescens*].

Surrounded by well-wishers as they were on their trip, the Lange’s had little opportunity to experience the exhilaration of city culture, which was in the midst of “The Roaring 20s”. The nation’s economy was growing almost too quickly, leaving the common worker behind. Not only was Prohibition, instituted in 1920, in full swing, but so were its violations. “Speakeasies” were ubiquitous. The Harlem Renaissance included the Savoy and Cotton Club; Cab Calloway, Duke Ellington and Ethel Waters performed there. The Lindy Hop and The Charleston were popular dances. The “Ziegfeld Follies” dazzled Broadway. The nation was still agog over Charles (“Lucky Lindy”) Lindbergh’s solo transatlantic aeroplane flight in May. American extravagant exceptualism was on display.

With their District of Columbia stop behind them, the Langes sailed for Europe in late autumn, 1927, perhaps to reach Odense in time to celebrate Morten’s birthday. Jakob seemed pleased with their tour, having delivered the message of the Danish cooperative movement and sharing experiences and philosophies with many people of similar views. The movement in the United States was represented by numerous eastern institutions, from colleges to folk schools to dreams in the minds of progressive individuals.

## ﻿Chapter 3. 1931

As aethereal as the United States economy and society were during Jakob’s first visit in 1927, just so low was the country during his second, in 1931. In October, 1929, the Wall Street bubble burst, the Stock Market swooned and by 1931, there were bread lines across the country. Herbert Hoover (1874–1964) was President, but there were rumbles of disappointment. Prohibition was still the law of the land, but violations were even more prolific than during Jakob’s first US visit. “Shanty Towns” and “Hoovervilles” of displaced workers existed in the squalid shadows of all US cities. Although some of the elite families had lost everything, many others had not and societal stratification was more severe than ever. It was the juvenile stage of “The Great Depression.”

As marginal as mycology had been for Jakob’s prior visit, so his political and cultural ideas were in 1931.

The Mycological Society of America did not exist in 1931 (Fitzpatrick 1932, 1934). Mycologists met as a section of the American Academy for the Advancement of Science. Nonetheless, local to national forays took place where mycological professionals could rub shoulders over specimens and breakfasts.

Lange (1931) “This second trip had partly other purposes and therefore also a different character than the first. It was mostly about botanical items, especially, of course, research on mushrooms. During my first trip, I naturally now and then had looked at the mushroom flora in different areas and found that it was mainly composed of the same species as at home. But in the books about American mushrooms I mostly found “American” species mixed with relatively few European. It was quite clear to me that this most likely was caused by, so-to-speak, a misunderstanding of each other’s language, so what in Europe was called by one name had another name in America. [But by now] I had ... been painting portraits of Danish mushrooms for a long time - about 1000 species. And I got the idea to go there (US), bring the pictures and gather the American mycologists and discuss with them, armed with the [common] background of the pictures and in this way come closer to the ’truth’.

“I sent my plan to the leading universities and to my private correspondents in the US and had, in this way, in 1931, planned such small mycological meetings in different areas, at Universities in the states of New York (Cornell), Michigan (Ann Arbor), Canada (Winnipeg), Oregon (Corvallis), at the Department of Agriculture in Washington [DC], in the forest- and lake-camp in the Adirondack Mountains and finally the mycological society in New York. So, it was a rather comprehensive plan, but the Americans were very interested and took good care of the arrangements. The largest approval was at Cornell where mycologists from about ten states (about 50 all told) were summoned and where a large apparatus with microscopes etc. was present and excursions arranged. The whole business had for me the important result that it was living proof of the value of such illustrated works and thus shaped the background for my thoughts of publishing my large mushroom-picture-work [*Flora Agaricina Danica*], which was realized starting in 1934 (Lange 1935a) and now is close to being finished.

“The Carlsberg-Foundation and the Rask-Ørsted-Foundation supported my plans and this along with the lecture payments made an economic balance possible, even if we were three, since both Leila and our 12-year-old son, Morten, joined the trip”.

In his English language report (Lange 1934), he gave a somewhat different itinerary. “Setting out from New York in the middle of August, my itinerary took me through Massachusetts, Vermont, the Adirondacks, Michigan, Minnesota, part of the territory south of Lake Winnipeg to the Canadian Rockies and the Pacific coast from Vancouver to Los Angeles and back to the south-eastern States, returning via Washington to New York towards the end of October”.

Lange’s purpose was not only to experience American mushrooms and to share opinions with American experts, but to exercise his concepts of ecology, distribution and taxonomy of both the mycota and flora. He was well-versed in the northern European groups. In his English report of the 1931 trip (Lange 1934), he outlined his concept of similarities between the European and North American floras (Lange 1913). “When a European botanist makes the acquaintance of the plant life of north-eastern America, he cannot avoid being impressed by the similarity of the floras on both sides of the Atlantic. To be sure, he encounters leading American genera, such as *Aster* and *Solidago*, represented by hundreds of species, which are only scantily represented in the European flora and vice versa. But apart from these, the flora on the American side consists mainly of two types: the introduced species and the parallel ones (i.e. species which, although not identical with the European ones are so similar that it often requires a keen and practised eye to distinguish between them)”.

However, did these designations also apply to the mycoflora?

Lange (1934) “That certain fungi have a universal distribution is a well-known fact. These cosmopolites are either native (e.g. many coprophilous fungi, such as Coprini) or they are introduced from one continent to another, having become established in their adopted home, sometimes even more fully than in their native land. This is true of numerous parasitic forms, such as the potato-blight (*Phytophthorainfestans*), the gooseberry-mildew (*Erysiphe Mors-uvae*) and the hollyhock-rust (*Puccinia Malvacearum*)”.

“On the other hand, it is also a well-established fact that the flora of any continent, besides these cosmopolites, comprises an element of endemics: species and even genera, which are exclusively American or European. In the phanerogamic plant world, the overwhelming majority of the species are decided endemics. But what about the mycoflora? Is the main body of the American fungus-flora decidedly ‘American’ or does it consist for a large part of cosmopolitan species which are familiar to the European mycologists of old and, therefore, in common parlance are called ‘European’ species? And finally, are the specifically American species mainly parallels or are we more likely to meet with species which represent other types, widely separated from those met with in the Old World? In other words, is the American fungus-flora chiefly characterized by congruency, parallelism or incongruity?”

An anecdote was inserted: “But stronger and more lasting than any other impression is the evidence of the wonderful cosmopolitanism of the Agarics. When you have once found, in a Danish *Sphagnum*-bog, a few specimens of the ‘new’ species *Strophariapsathyroides* Lange, it gives you a shock to meet with the very same plant in a bog in Oregon, near the Pacific Coast and only an hour later to come upon *Lepiotacygnea* Lange, of which the only known specimens were theretofore those gathered in 1925, a few miles from my Danish home”.

Lange furnished examples of agarics he considered to belong to these categories and, in “*Flora Agaricina Danica*” (FAD), repeated some of his conclusions (Lange 1935b).

In America, word spread of Lange’s proposed appearance. Harry Morton Fitzpatrick (1931), a ringleader of the movement to establish a formal mycological society (Fitzpatrick 1934) and a professor at Cornell, issued an invitation: “Doctor Jacob [sic] E. Lange, well known Danish student of the mushrooms, will arrive in New York the middle of August for several weeks of collecting in the north-eastern United States and eastern Canada. He wishes to study especially the parallelism and identity of American and European species of Agaricaceae. A definite itinerary has been arranged. Inquiries regarding its details may be directed to Doctor C.W. Dodge at Pawlet, Vt.

“From August 28 to September 2 inclusive, Doctor Lange will be at Ithaca, N.Y. The region about Ithaca is especially interesting to him because Atkinson published over a period of years on locally collected materials. Fungus forays will be made daily to nearby points of interest in the effort to see a large number of species.

“In order that the conceptions of species as held by Peck, Atkinson, Kauffman and other older American workers in the group may be clearly understood, it is imperative that Doctor Lange be enabled to exchange ideas in the field with their students. To this end, American mycologists, especially those interested in mushrooms, are urged to come to Ithaca and cooperate in making these forays a success. Students with only a minor interest in the Agaricaceae will also be welcomed and the foray will be arranged in such a manner that collecting in other groups will be fruitful. Incidentally, the Atkinson herbarium has been put in good order in recent years and is now available for consultation in the new Plant Science Building at Cornell University.

“Those who plan to attend the Ithaca forays are asked to notify the undersigned at as early a date as possible. Arrangements will be made for lodging, meals and transportation at a reasonable rate. Information concerning these items or other features of the plans for the forays will be gladly given”.

On his previous trip in 1927, Lange had emoted on the New York City skyscrapers, but now in 1931, he must have marvelled over the new Art Deco-decorated Chrysler Building, opened almost simultaneously with the Stock Market crash in 1929 and then soon eclipsed by the Empire State Building some blocks south, opened in 1930. For many later years, they would dominate the city’s architecture for travellers arriving by luxury liners or later by aeroplane.

Before relating Lange’s “study-tour,” it is necessary to acknowledge the clues left in the collections made during his visit which remain in herbaria. MycoPortal (2021) can be sorted by collector and date and when searched for Lange and 1931, the result is 14 such collections, but just as important, the dates of each collection, location, in some cases, his co-collectors and the herbaria housing these specimens are disclosed.

Harvard, Cambridge, Massachusetts (dates unknown). Lange did not detail a stop at Harvard, but his comments on some of the original mushroom illustrations by Bridgham and Krieger, commissioned by William G. Farlow, attest to his presence. Donald Pfister (1975 & pers. comm.) observed that Lange had access to more than 600 such paintings, from which only 103 plates were composed (Farlow 1929). Although the format of Lange’s “Flora Agaricina Danica” differed from that of Farlow’s “Icones”, the two publications were not totally different in concept.

**Figure 13.**
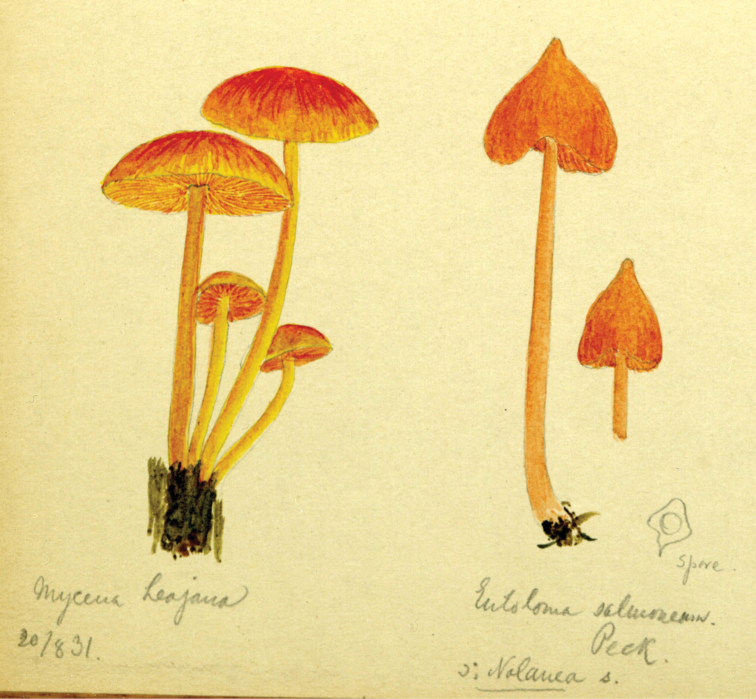
Lange aquarelle sketch dated 20 August 1931 (Pawlet, VT). Left: *Mycenalaeiaena* (“*jaejana*”). Right: *Entolomasalmonea*. Courtesy Natural History Museum of Denmark, Copenhagen.

Vermont (18, 19 August). Although several destinations of Lange’s itinerary were anticipated, some were more explicit. An example of the latter was his visit to Vermont. That such a foray actually existed is confirmed by Lange’s (1941) mention of collecting *Volvariapubescentipes* Peck. An additional sketch (Fig. 13) confirms this. MycoPortal records five such collections, all housed at the Farlow Herbarium, Harvard, perhaps indicating attendance by David Hunt Linder (1890–1946; Rusden 1947) and/or William H. (“Cap”) Weston (1890–1978; Wilson 1979).

The family home of Carroll William Dodge (1895–1988: see above) was in Pawlet, southern Vermont. He had studied at Middlebury College and, there, had come under the influence of Edward Angus Burt, who soon moved to St. Louis as Professor at Washington University and mycologist at the Missouri Botanical Garden (Petersen 2019). Dodge also moved to St. Louis and received his PhD from Washington University in 1918. After a hitch in the Army (World War I) and some time at Harvard, in 1931, he was packing his belongings for a move from Harvard to take up the position held previously by Burt. Probably, it was the family home in Vermont that the informal foray of 1931 used as headquarters.

It was the first foray for Lange in the United States and he had not yet established a routine. He took time to make eight water-colour sketches and recorded more than 40 names in his notebook.

**Figure 14.**
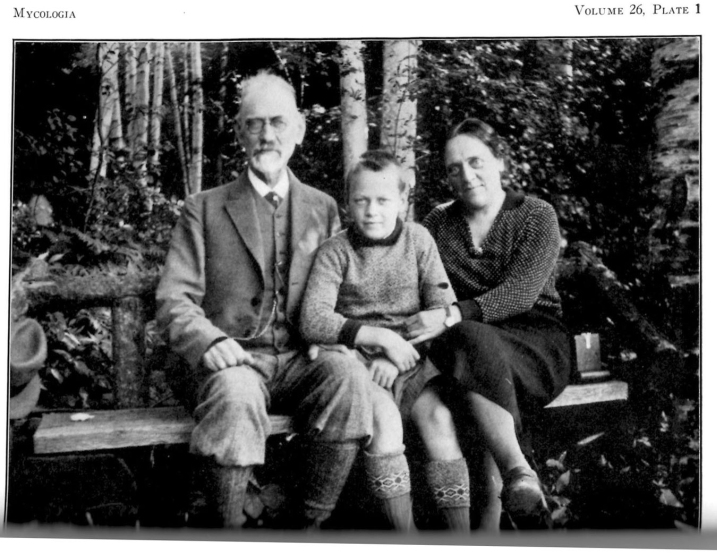
The Lange family, probably in the Adirondacks in 1931. Source: Mycoologia 26(1): 2.

**Figure 15.**
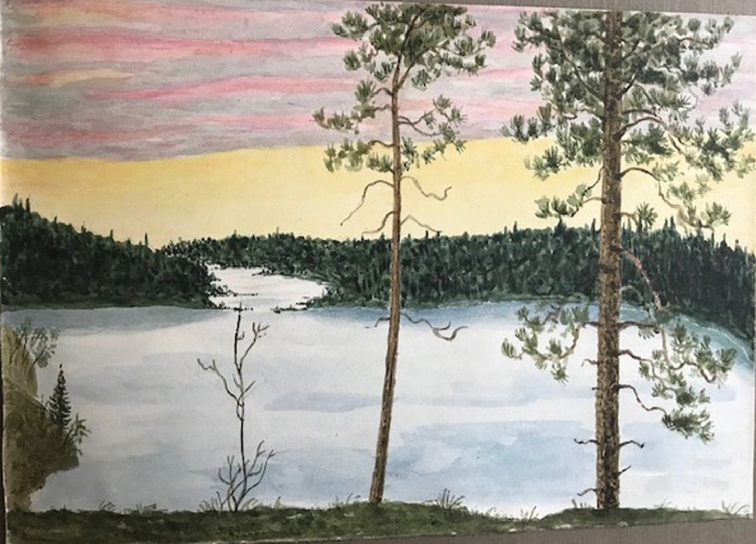
Jakob’s painting of the scene at Seventh Lake, New York. Source: Lene Lange.

Seventh Lake, The Adirondacks, New York (20–25 August). The first widely announced stop on Lange’s itinerary was a select foray in the Adirondack Mountains of New York. Fred Carleton Stewart (1868–1946), plant pathologist at Cornell, had a summer “camp” on a lake-shore in the Adirondack Mountains (Fitzpatrick, 1947; Gruff 2007) and, on more than one occasion, he opened its “spartan” facilities to mycological forays for small groups, including the Lange family in 1931 (Fig. 14). A typical scene was painted by Jakob, unmistakable with its “white pines” and evergreen forest and, perhaps, Seventh Lake (Fig. 15).

Lange (1941) reported that a relatively rare species, *Lactariusrepraesentaneus*, was observed and he compared it to the European *L.deliciosus*. MycoPortal lists two collections, *Inocybelongicystis* and *Pholiotaspumosa*. The first remains at Cornell (probably deposited by Fitzpatrick), the second at the Farlow Herbarium, again perhaps indicating attendance by Linder and/or Weston. Lange completed eight water-colour sketches and registered (questionably) some 60 names in his notebook.

Years later, in his memory of the affair, Hesler (1975) furnished a rare physical description of Jakob Lange. “… I recall that, in my good fortune, I attended the Adirondack Foray in 1932 [sic]. Lange brought with him his 200 completed plates, later published in FAD. The weather being clear and calm, one day he spread all the plates over the lawn of our cottage for us to view. I recall also that one of his paintings (a *Lepiota*) was made by Lange out on the lawn, where he used a barrel for a table and an orange crate for a stool. To complete this painting required some fifteen or twenty minutes, as I witnessed the job. It appears perhaps as one figure in either plate 12 or 13 [of FAD].

“Lange also brought with him his wife [Leila] and his son, Morten, then about 8 years old [actually 12] and, at that time, he [Morten] knew many of the agaric genera at sight. (Dr. Morten Lange has for some years now been mycologist in The University of Copenhagen [written in 1975]).

“… He (Jakob) was of rather exceptional physique, perhaps 6 ft., 4 in. height, 190 lbs., lean, good humored, a delightful personality, he spoke and wrote excellent English and, as a conversationalist, one of the best. His devotion to the people led him, while here at the foray, to visit the Penland (N.C.) Folk School”. While certainly possible, Lange surely visited the John C. Campbell Folk-school, also in North Carolina.

**Figure 16.**
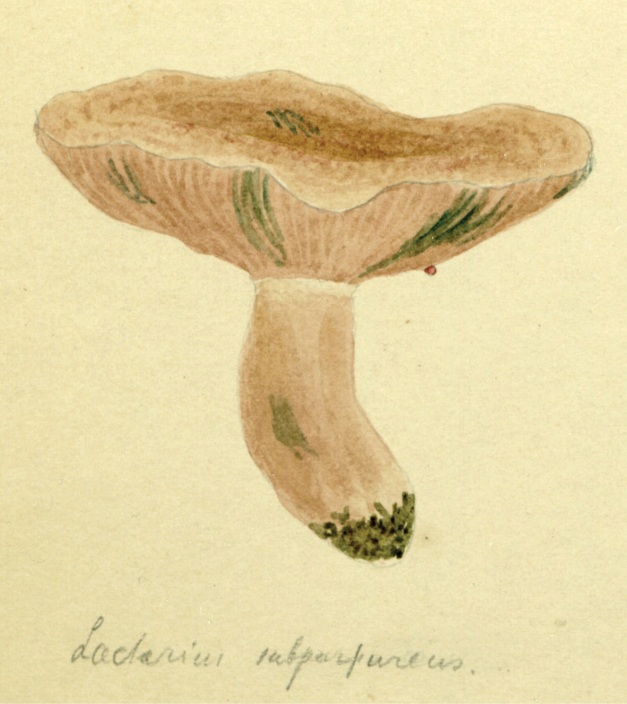
Lange aquarelle sketch dated 28 August 1931 (Cornell foray). *Lactariussubpurpureus*. Courtesy Natural History Museum of Denmark Copenhagen.

**Figure 17.**
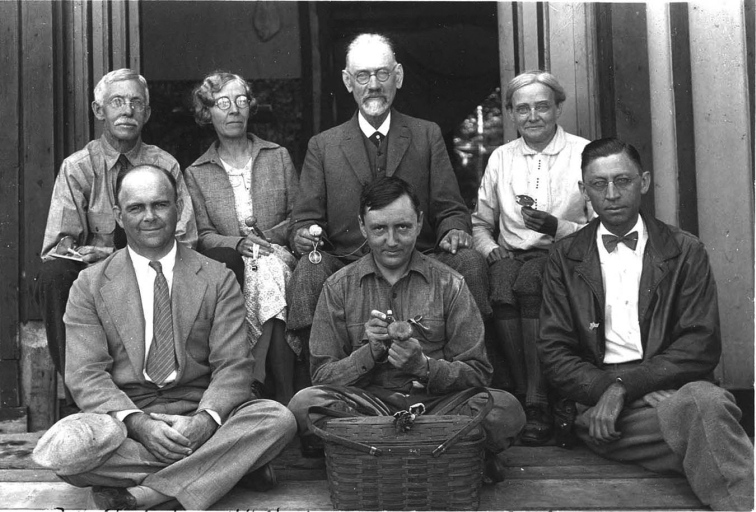
Mycological group at Cornell. Left to right: F.C. Stewart, H.M. Fitzpatrick, Vera Charles, Jakob Lange, C. W. Dodge, Gertrude Burlingham, L.R. Hesler. Source: Cornell University.

Cornell University, Ithaca, New York (25–28 August). Although MycoPortal data show a month-long hiatus between the Adirondack foray (26.8.31) collections and those from Corvallis, Oregon (27.9.31), Lange’s notebooks and paintings make the Cornell dates more precise. Fitzpatrick’s announcement of the Cornell event set the dates as 28 August through to 2 Sept. In his post-tour report (Lange 1934), Lange noted that about 50 mycologists of all specialties, from some 10 states, gathered for those days. Forays visited some of Atkinson’s (and his student, Calvin Kauffman) favourite collecting spots. Korf (1991; Petersen 2021) wrote that at least three of these well-known collecting grounds around Ithaca had been purchased sub rosa by Curtis Gates Lloyd (1859–1926) and were to become known as the Lloyd-Cornell Preserves. Strangely, MycoPortal lists no collections from these forays. If collections were preserved, their identifications, locations, collectors and herbaria remain undiscovered. Lange painted only two mushroom sketches (Fig. 16), the last of the entire trip, surely reflecting a more hectic routine resulting in copious notebook entries. He logged 50+ names. A list of participants has not been unearthed, but an iconic photo shows F.C. Stewart, H.M. Fitzpatrick, Vera Charles, Lange, Gertrude Burlingham, C. W. Dodge and L.R. Hesler, an august assemblage (Fig. 17).

Hesler’s interest in the Adirondack and Cornell forays remains somewhat murky. Before and after his arrival at the University of Tennessee in 1919, he was a phytopathologist, distinctly in the mould of his Cornell PhD under H. H. Whetzel (Petersen 1978, 2021; Korf 1991). By his own recollection, Hesler’s migration from plant pathology to mushroom taxonomy came much later, at about the time of the Adirondack and Cornell forays. He had begun to show interest in the fleshy fungi of the Great Smoky Mountains and was known to hitch rides on logging trains in and out of the mountains during the mushroom season. It was not until the catastrophic fire of 1934, which destroyed the entire botany department and all its faculty offices, laboratories, classrooms, library and herbarium that Hesler decided to point his career towards agaricology. What role his interaction with Lange might have had remains obscure, but he sent a signed copy of his “Mushrooms of the Great Smokies” (Hesler 1960) to Morten Lange “with personal good wishes”.

Ontario (?Ottawa; dates unknown): The discrepancy between Lange’s notebook dates for Ithaca and those in Fitzpatrick’s announcement may have provided enough time for a side trip mentioned only in passing in Lange’s notebooks. Lange’s described itinerary does not report any time in eastern Canada, but MycoPortal lists a single collection by Lange from Ontario, Canada, *Thelephoraterrestris*, on 16 September 1931 – nearly two weeks after explicit Cornell and well before implicit Vancouver – with no co-collector, but improbably housed at the University of Wisconsin, perhaps a link to a co-collector.

By searching for such a link, though, it might be coincidental that, in Toronto, Herbert Spencer Jackson (1883–1951) had taken charge of the fungus herbarium at the University of Toronto in 1929. He had degrees from Cornell, Harvard and the University of Wisconsin (PhD in 1929) and was a vigorous mycological researcher (Bailey 1952). It is pure conjecture that Jackson might have come south to participate in the Cornell forays and invited Lange to return with him to Toronto.

University of Michigan, Ann Arbor, Michigan: Again, this visit was noted only marginally, but Lange noted the water-colour illustrations in the collection of Dr Howard Atwood Kelly (1858–1943), executed by Lewis Charles Christopher Krieger (1873–1940; Petersen 2019), which had been recently bequeathed to the University and housed in the herbarium. Krieger’s illustrations surely rivalled those by Lange himself. No field trips or specimen numbers were recorded in Lange’s notebooks.

Edwin Butterworth Mains (1890–1968; Smith 1969) was a Michigander by birth and rearing. After starting at Michigan State University, he transferred to the University of Michigan for his B.A. and earned a PhD in 1916, with Calvin H. Kauffman as major professor. After some years at Purdue University, Mains returned to become a professor at the University of Michigan in 1930 (after the debilitating illness of Kauffman). Kauffman’s illness and death must have been a disappointment for Lange: Kauffman was an enthusiast of colour photography for illustrating mushrooms and had used black & white photographs for his volumes on the agarics of Michigan (Kauffman 1918), while Lange insisted on watercolours. Kauffman also had experience with western agarics and so could have prompted Lange as to what he might find there.

Bessie Bernice Kanouse (1889–1969) was also a Michigander through and through. Her B.A. was from the University of Michigan, as was her PhD in biology in 1926. She was appointed Curator of the University of Michigan Herbarium and assistant to the Director, Calvin H. Kauffman. She accompanied Kauffman on at least two collecting trips, but her own interest was in “water moulds,” on which she published (Kanouse 1927a, b).

Notably, Kauffman’s student, Alexander Hanchett Smith (1904–1986) had been obliged to change professors upon Kauffman’s illness and now was being supervised by Mains. Smith’s M.A. was gained in 1929 and his PhD dissertation, “Investigations of two-spored forms in the genus *Mycena*” earned his PhD in 1933. Conversation with Lange could have been lively. As Atkinson and Kauffman before him had used photography (Petersen 2019), Smith was to use this medium through his entire career. Moreover, the first instalment of Lange’s “Studies” series was on *Mycena* (Lange 1914), so taxonomy could have been discussed as well as methodology. Pat Rogers, currently collection manager for fungi at the University of Michigan, commented that Smith spent extended time in Nova Scotia, Canada, during the time of Lange’s visit, perhaps as assistant to Lewis Edgar Wehmeyer (1897–1971; Wehmeyer 1934).

Just a few buildings away, Dow Vauter Baxter (1898–1965; Smith 1967) was a forest pathologist in the School of Natural Resources (now the School of Environment). An inveterate field botanist, he was already engaged in a long-term study in Alaska. Whether or not he met Jakob Lange is unknown to us.

A half-day’s ride from Ann Arbor was East Lansing, the home of Michigan State University. In 1931, Ernst Athearn Bessey (1877–1957) was serving there as Dean of the Graduate School, building its programme in plant pathology, amongst many others. Whether he might have met Lange is unknown to us, but it would surely have been possible. In addition to his Deanship, Bessey was developing his book, “A Textbook of Mycology” (Bessey 1935).

Minnesota (perhaps also Wisconsin): Although both States were merely proposed, but not further mentioned in Lange’s notebooks, these States had a wealth of Scandinavian settlers and universities with strong phytopathology research faculties.

Lange’s reference to a stop in Minnesota is opaque. One possibility might have been in Minneapolis, where Clyde Martin Christensen (1905–1993) was a graduate student at the University of Minnesota. Christensen (2021) obtained his B.A. there two years before (1929) and his M.S. in 1930. In 1934, he joined the faculty and was awarded his PhD in 1937. He spent his entire career at that university until 1975 and retirement. While his research did not emphasise fleshy fungi, he wrote two popular volumes on the subject (Christensen 1943, 1946), neither of which bore reference to Lange.

The Minnesota Mycological Society was founded in 1899, as the second oldest such organisation in the country (Boston was first, four years previous). For some years, its founder, Dr Mary Whetstone,^(8)^ was corresponding secretary (Dillon 1924; Davis and Seaver 1930). Her considerable correspondence (and, apparently, associated specimens, usually fresh!) with Charles Horton Peck (1833–1917), Curtis Gates Lloyd (1859–1926), F.J. Braendle [USDA botanist in Washington, DC; dates unknown; for whom Peck (1899), found some to be new] and others often led to identifications and return letters. Clements’ (1910) popular mushroom book was available, even although Clements had moved on. Given the host of Scandinavian and German settlers over this land of lakes, forests and biting flies, it would have been no wonder that mushroom hunting was popular and a European expert would have been welcome to join forays.

Although still marginally known for “pure” mycology in 1931, the University of Minnesota was a juggernaut in plant pathology, especially cereal diseases. By 1931, Elvin Charles Stakman (1885–1979) headed the Plant Pathology Department of the Division of Vegetable Pathology and Botany at the University (Peterson 2001). Amongst many other students and post-doctoral fellows, he acted as consultant to A.H.R. Buller at the Manitoba Agricultural School and was largely credited with the establishment of plant pathology in Canada. Although marginal to Jakob Lange, Stakman and his fellow faculty would have been politically cogent conversants.

Winnipeg, Manitoba; Longpine Lake, Ingolf, Ontario, Canada (14–18 Sep): Unlike the forests encountered on both coasts, south-central Canada is located at the northern rim of The Great Plains. Although less rich in large mushrooms, careful inspection of scattered large groves of aspen and/or conifers could produce results. Lange (1941) noted a collection of *Xeromphalinacauticinalis*, surely found under conifers, but MycoPortal does not record any such. Enigmatically, collection numbers from 175–190 were noted by Lange, as well as a single sketch (*Lepiotaillinita*). The collection numbers overlapped those later recorded from both Lake Louise and Vancouver.

Guy Richard Bisby (1889–1958) was a professor at the Manitoba Agricultural College (later University of Manitoba) in Winnipeg. A South Dakotan by birth, Bisby earned his B.A. from South Dakota State College, his Master’s degree from Columbia University (NY) and PhD from the University of Minnesota in 1918 (Purdue 2021). In 1920, Bisby moved to Winnipeg (and gained citizenship of Canada; Anonymous 1932a). In 1937, he accepted a position with CMI, Kew. His research centred in compilation of floristic lists, “The Dictionary of Fungi” (with Geoffrey Clough Ainsworth, 1905–1998) and nomenclature of fungi (Bisby 1946). Although editors changed over time, “Ainsworth & Bisby’s Dictionary of the Fungi” reached its tenth edition in 2008 (Kirk et al. 2008).

Lange (1931): “One of the funniest of these gatherings - but we were only two, a Dane [mycologist] and a Canadian (+ Morten) – was when we came up to Winnipeg in southern central Canada, close to the large Lake Winnipeg. When we arrived, we were met at the station by Dr. Bisby; he immediately asked my wife if she could cook. ’Yes, at the household level,’ she answered. ’Then the plan can be realized,’ he said. ’I have borrowed a cabin in the wilderness, 20 miles from here, there we will go for some days’”.

The cabin was actually Longpine Lake, near Ingolf, a short distance over the provincial line in Ontario. There, they lived in a cabin as noted by Bisby. His human side was revealed in a small note by Morten (Lange 1996): “The first month [of our trip] I avoided English conversation. It greatly improved when we came to Canada and lived in a hut at a lake in the forest near Winnipeg. Our host, Professor Bisby, took me on his daily tour in the canoe to the grocery store. In the quietness of the canoe, he quietly convinced me that I probably could speak English, when he was the only one to listen”.

Some details of those days can be found in MycoPortal and a search for appropriate collections in the National Herbarium of Canada at Ottawa (DAOM). Arrival in Winnipeg 14 Sep (a Monday), was spent in the town, with some time devoted to a stroll on the campus of Manitoba Agricultural College (with seven collections). It is assumed that the 15^th^ was spent in transportation to the hamlet of Ingolf, about 145 km away; Longpine Lake was nearby. Upon arrival, only three collections were made, but on the 16^th^, at least 32 samples were collected and, on the 17^th^, 41. On the 18^th^, only 12 specimens were collected, but on that day, it may have been necessary to pack and head for Winnipeg.

A couple conclusions may be drawn from these data. First, Guy Bisby was a very organised host. He must have organised a drier and some way to bring at least 96 collections back to Winnipeg. Moreover, these collections were labelled appropriately and sent to Ottawa for deposit in DAOM. Second, in 96 collections, there was not a single duplicated name. Third, aside from four non-agaric fungi, all other collections bear names of mushrooms. Fourth, a rough review of collection labels shows a typical roster of autumn mushrooms, at least further east in North America, dominated by *Cortinarius*, *Lactarius* and *Russula*. Sixth, while all collections bear both Lange’s and Bisby’s names as collectors, there is no notation on the name of the identifier. It is, therefore, impossible to know if the fungi were recognised as “typically European” by Lange or by Bisby as “American” with European authors’ names.

A fellow faculty member of Bisby in Winnipeg was Arthur Henry Reginald Buller (1874–1944; Estey 1986). An immigrant from Great Britain, he arrived in Winnipeg in 1904. He was allowed to spend three or four months per year back in Birmingham, England or, later, at Kew, doing research in laboratories and libraries. By the time Lange came through southern Manitoba, Buller had published the second volume of “*Researches on fungi*,” which would eventually (and posthumously) reach seven volumes (Buller 1922). In addition to this major achievement, Buller wrote scores of individual papers, usually in the “Transactions of the British Mycological Society”. In spite of the popularity of his pedagogical fieldtrips, whether Buller joined Lange and Bisby or whether the Langes stopped in Winnipeg is unknown to us.

Although John Dearness (1852–1954) was retired in 1922, he remained active, amongst other contributions, as co-author of “The Fungi of Manitoba” (Bisby et al. 1929, 1938). His entire mycological career, however, was spent in western Ontario and he may have met Lange if he (Lange) travelled to Ontario after the Cornell forays.

**Figure 18.**
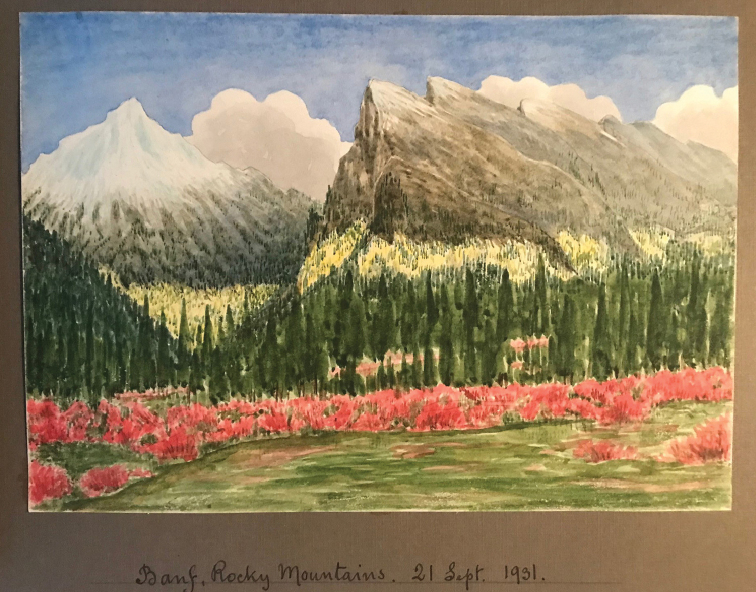
Jakob’s painting of the scene in Banff, Alberta, Canada. Source: Lene Lange.

Lake Louise, Alberta, Canada (including 21 Sep): Included merely as a mention in Lange’s itinerary, collection numbers 178–183 and 191–195 were recorded. These numbers overlap those from Long Pine Lake, Manitoba. There was time for a “portrait” of Banff (Fig. 18).

**Figure 19.**
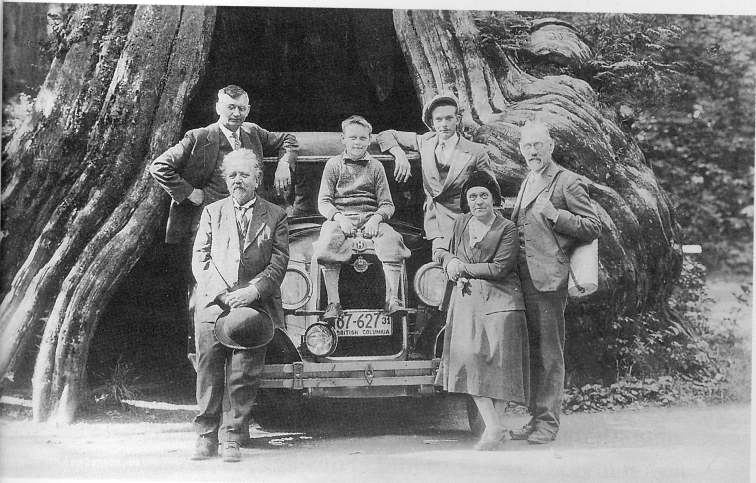
The Lange family near Vancouver, Canada, 1931. “Chain-restaurant owner Frøstrup, teacher Chistensen and former pupil at the *hudsmansskole* Knudsen”. Source: Lange 1969? Centre, Morten Lange; rightmost, Leila and Jakob Lange.

Vancouver, British Columbia, Canada (approx. 23 Sept): In an easily overlooked paper in *Mycologia* on Vancouver agarics, Jean Davidson (1930) thanked “Prof. F[rank] Dickson of this University” [Univ. of British Columbia, Vancouver] for his help in identifying her collections and the writing of the paper. English born, Frank Dickson (1891–1969; Yarwood 1970) was the first instructor of plant pathology at the University and taught forest pathology and mycology there from 1923–1956 (Estey 1994). In addition to his duties on campus, Dickson was part of the staff at the Aleza Lake Research Station (2007), over a hundred km north of Vancouver, probably during the summer months. Although only a slim chance, Aleza Lake may have been Lange’s opaque reference to the Canadian Rockies. Dickson’s name appears in the directory of mycologists of the British Empire (Anonymous 1932a). Morten Lange provided a photo of the family with some local Danes in front of a great tree in the vicinity of Vancouver (Fig. 19). Otherwise, little was made of the stop. Collections numbers 178–187 and 191–195 were logged, but these numbers overlap those from Manitoba and Alberta.

Seattle, Washington (dates unknown): The data confirming a stop in Seattle are sparse; only collection numbers 202–222. To be sure, to have missed this venue would have been unfortunate, for it offered potentially excellent collecting grounds.

One of the earliest papers on west coast fungi was Murrill’s (1912a), but it was of little help when dealing with fresh agarics in the field. In fact, Murrill made his way down the coast and MycoPortal reveals that he collected in the vicinity of Seattle, Corvallis, and Newport, Oregon and even as far south as the Santa Cruz mountains below San Francisco.

John William Hotson (1870–1957), a Canadian, came to the University of Washington, Seattle, in 1911, like Murrill and soon was teaching several courses, including botany, forest pathology, general mycology and elementary agriculture. His Harvard PhD was granted in 1913. Early in his Washington career, he came into contact with Sanford M. Zeller who later took an appointment in Corvallis, Oregon, as a plant pathologist (Ammirati, pers. comm.) and with whom Lange collected during his 1931 trip.

Hotson organised the first fungus herbarium at Washington and many of his specimens are cited in treatments of *Agaricus*, *Amanita* and other genera of agarics.

Daniel Elliot Stuntz (1909–1983; Stuntz 2021), soon to become the mycologist at the University, was a junior undergraduate when Lange visited in 1931. Whether he and Lange met, through Hotson of course, is unknown, but perhaps Stuntz’s interest in fungi was spurred by Lange’s presence. No record of field trips survives, but fertile field potential extended from the Cascades to the north, Mt. Rainier to the south and the Olympics to the west. Perhaps Hotson related Calvin Kauffman’s comment on west coast mushrooms: “In 1922, when Dr. C.H. Kauffman visited this state on a mycological trip, he made the remark that ‘… as a rule the mushrooms of Western Washington and Oregon are Friesian or new’, indicating that our forms are more like those found in Sweden than those of eastern United States” (Hotson 1936). To this, Lange could add his own observations. Kauffman’s (1925, 1930) papers on fungi of relatively nearby areas preceded Lange’s visit and may have spurred Lange’s interest in the forests of the west coast.

Perhaps Lange’s visit may have spurred Hotson to put his mycological experience into words (Hotson 1934, 1936) and he encouraged students to also pursue work on the agarics (Hotson and Lewis 1934; Hotson and Stuntz 1938). In the paper with Stuntz, Lange’s “*Studies*” (no. 6, 1926) and his 1934 report of his United States trip were cited. As a grace note, however, their illustrations of the agarics of the Pacific Northwest were all photographs.

They also echoed Lange’s sentiments this way: “The genus [*Agaricus*] is well represented in the Pacific Northwest, but, when one wishes to identify collections made in this region, he faces an obstacle in the lack of available information concerning the local flora and also in the fact that some of the species occurring here are more closely allied to European forms than to those found in the eastern United States. To further complicate matters, the student will often find a lack of agreement among mycologists concerning the interpretation of a particular species and is apt finally to be more confused than helped by the literature he consults. This condition has been found as prevalent in Europe as in America” (Hotson and Stuntz 1938).

Corvallis, Oregon (approx. 27 Sept): Sanford Myron Zeller (1885–1948) was the mycologist at the Oregon Agricultural Experimental Station (associated with Oregon State University) in Corvallis. His PhD was from Washington University in St. Louis, where Edward Angus Burt was the mycologist. At the time of Lange’s visit, Zeller’s research emphasis was on parasitic fungi, less so on gastroid fungi (Gilkey 1949), including hypogaeous forms, the latter a group not previously investigated by Lange, but which he noted when discussing his itinerary. Together with Zeller, at least two field trips were undertaken (only 27 Sep appears in MycoPortal), although their reported destinations were hardly compatible in one day. On one foray in the vicinity of Mt. Hood, collection numbers 223–237 were recorded. On the second, to the Coast Range, Lange (1941) later reported finding his own *Lepiotacygnea*, not yet published, but theretofore known only from a couple collections from his home in Denmark (Zeller 1938), amongst collection numbers 238–248. Equally, what Lange knew as his own species, *Strophariapsathyroides* from a Danish *Sphagnum* bog, was discovered in the same habitat in Oregon. MycoPortal reports three specimens: two of *Lepiotaoculata* and one of *Gomphidiusnigricans*.

At the University, Helen Margaret Gilkey (1886–1972) was Professor of Botany and Curator of the Herbarium. Her Master’s degree thesis entitled “Oregon Mushrooms,” had led to doctoral work at the University of California and her PhD dissertation, with William Albert Setchell as major professor, was “A revision of the Tuberales (truffle fungi) of California” (Leonard 2021). With this background, she was already a likely contact for Lange, but her talent as an artist-illustrator might well have matched Lange’s insistence on accurate depictions. Lange, however, saw little value in dried herbarium specimens and coloured portraits of Tuberales were less than helpful. They might have differed over this subject. Gilkey’s (1939) later magnum opus took in all of North America and was accompanied by superb illustrations.

Berkeley, San Francisco Bay Area, California (visit problematic): Surely, a most conjectural stop for Lange and family was the California Bay Area; San Francisco and especially the University of California, Berkeley. Lange may have seen Edwin Bingham Copeland’s (1873–1963; 1904a, b) small series on new and interesting California fungi, but these papers were written years before Lange’s tour, when Copeland was a student at Stanford University in Palo Alto and before his PhD from Halle, Germany. As coincidence would have it, Copeland was changing venue in 1931 from the Philippine Islands to Berkeley, where he was less familiar with mycological surroundings. Copeland was to become internationally known for his work in pteridology (Wagner 1964, 1965).

A person with a much longer career in Berkeley was William Albert Setchell (1864–1943; Drouet 1943). Setchell was already better-known in phycology than mycology, although he had acted as supervisor for Helen Gilkey’s Master’s degree. She may have advised the Langes to visit Setchell.

Elizabeth Eaton Morse (1864–1955; Bonar 1956), a New York City teacher, made her summer vacations into naturalistic trips, one of which was to Berkeley in 1925. There she stopped and inquired as to any summer courses in botany. There was none, so she proceeded to Yosemite National Park to collect fungi. She never returned to the east, but was put on staff at Berkeley, remaining so for many years as curator of fungi and all-round mycologist (Vellinga 2020). She could have been a candidate to take the Langes into the field in Berkeley’s environs.

Elmer Drew Merrill (1876–1956; Robbins 1958) had served as Dean of Agriculture at Berkeley, but, in 1929, had moved on to become Director of The New York Botanical Garden and afterwards to the Arnold Arboretum of Harvard.

It must be written again, however, that we have no evidence that Lange stopped in the Bay Area, so his possible interaction with any of the triumvirate of botanical friends is purely conjectural.

Los Angeles, California (dates unknown): Los Angeles appears only as a single-word mention in Lange’s proposed itinerary. The nearest predictable mushroom-hunting grounds were in the mountains away from the coast, but even more improbably, mycologists were also less prolific.

One person with mycological experience and resident in the Los Angeles area was Effie Almira Southworth Spalding (1860–1947; Southworth 2021). Reportedly, she was the first woman hired in a mycological field by the USDA, including those in the Washington, DC, headquarters and her story is interesting.

Born in New England, she attended Allegheny College for a year, but transferred to the University of Michigan, where she was awarded a B.A. in 1885. She immediately became an instructor at Bryn Mawr College, near Philadelphia, on a two-year contract. In 1887, she was hired by the USDA as the third person in the Section of Mycology, along with Erwin F. Smith and Beverly T. Galloway and moved to Washington, DC (Ristaino and Peterson 2013; American Phytopathological Society 2001). Her paper on cotton cankers was a major advancement in plant pathology. Her short paper in the “Bulletin of the Torrey Botanical Club” dealt with an assumed polypore – perhaps a large sclerotium (Southworth 1891). Five years later, she became an Assistant in Botany at the newly-established Barnard College in New York, where she must have interacted with Lucian Underwood, the mycologist at Columbia University across Broadway from Barnard. She married Volney Morgan Spalding, a botanist at the University of Michigan in 1895 and moved there. Soon, though, his health began to fail. Eventually he was diagnosed with tuberculosis and the couple moved to Tucson, Arizona. There, he worked at the Desert Botanical Laboratory of the Carnegie Institute of Washington. Retired in 1909, he spent the rest of his years in a Loma Linda, California, sanatorium, where he died in 1918. Almost immediately, Effie was hired to the faculty of the University of Southern California as Assistant Professor of Botany. There she spent her remaining years, during which she was awarded an M.A. in 1922, with a thesis entitled “Form alterations and growth in cacti,” established the herbarium and wrote occasional papers on plant pathological fungi. She died years later, in 1947. Her name was mentioned as a notable early mycologist by Maroske and May (2018).

That she was on faculty and could have met Lange in 1931, is beyond dispute, but whether they, in fact, met, is highly questionable. She would have been 71 years old and a foray or two would have been improbable. What was much more expected, however, was that Los Angeles served as the Langes’ terminus along the west coast and their departure point for return to the east.

In 1922, Bonnie C. Templeton (1906–2002) moved from Nebraska to Los Angeles. According to Wikipedia (Templeton 2021), amongst several jobs she occupied during the following decade, one was assignment as an assistant to a botanist at the California Botanical Garden in 1928. She fell in love with the work of sorting and identifying plant specimens and the following year was appointed Curator of Botany at the Natural History Museum of Los Angeles County, a position she held from 1928 until 1970. Through night courses, she earned B.A. (1941) and Master’s (1947) degrees from the University of Southern California. As was common, she was refused permission to enter a doctoral programme at USC, but obtained her PhD from Oregon State University. She was a person of wide interests and MycoPortal records two specimens of fungi she collected in 1931, both of them earlier than Lange’s time in southern California. Whether they met is lost in history.

Although preceding Southworth by several years, Alfred James McClatchie (1861–1906), a Professor at Throp Institute of Technology (now known as California Institute of Technology, “Caltech”), was an active botanist, including fungi and published two papers which included fungi of Pasadena vicinity (McClatchie 1895, 1897). McClatchie was educated at Olivet College in Michigan, with an A.B. from the University of Nebraska, with Charles Bessey, in 1890. McClatchie’s Herbarium was sold to the New York Botanical Garden and Murrill (1912b) noted that McClatchie’s early specimens had been sent to Setchell, Ellis, Morgan, Peck, Underwood, Barnes and Hasse (the last two not familiar names to us: RHP, HK).

Also worthy of mention, Charles Fuller Baker (1872–1927; Essig 1927) collected fungi amongst many other biological groups in southern California at about the same time as McClatchie. Born in Michigan, he attended the Michigan Agricultural College (later Michigan State University). Spending much of his professional life in tropical regions, he was an inveterate collector; his entomological specimens alone numbered over 400,000. From 1899–1901, Baker was zoologist at the Alabama Polytechnic Institute and connected with the Alabama Biological Survey, where he was a colleague of Franklin Sumner Earle (1856–1929) just a few years after George Francis Atkinson (1954–1918). In 1903, Baker was on faculty at Pomona College in southern California, but his brush with the region was short-lived.

“Southern states” (Arizona, Kentucky, Tennessee; dates unknown): From MycoPortal, we know that the Langes were in the neighbourhood of Corvallis, Oregon, on 27 Sep. At that moment, however, the monitor goes black and the next time we have a firm date is 24 October, in Washington, DC. Assuming their mode of transportation as rail and Jakob’s reference to “the southern states,” we can conjecture that they took the Southern Pacific route through southern Arizona (Morten found the Grand Canyon “shocking”), New Mexico and Texas. Reference to the specific States appears only in Lange’s proposed itinerary, with no collection numbers recorded or sketches executed.

William Henry Long (1867–1947; Stevenson 1949), a Texan, obtained his B.A. from Baylor University in Waco, in 1888, after which he was appointed to faculty there until 1892, when he moved to Burleson College in Greenville. In 1899, he left Burleson to study at the University of Texas, where he received his M.A. in 1900. After nine years on faculty at North Texas State Normal School in Denton, a PhD was granted by the University of Texas in 1917. Several summers during this time, Long spent periods with G.F. Atkinson at Cornell University and, thus, rubbed shoulders with the numerous faculty and graduate students there in the Plant Pathology Department (Petersen 2021).

His professional career was at the USDA where he soon was transferred to the Office of Forest Pathology in the Bureau of Plant Industry in Washington, DC. There he found himself in a stellar group of mycologists/forest pathologists. Soon, he was appointed to head up this work in the south-western States, headquartered in Albuquerque, New Mexico. There, he stayed until retirement in 1937. Although his research emphasised tree rusts, he was interested in the gastromycetes and it was probably this that would have caught the eye of Lange. If the two met, possible field work for Lange would have been at relatively high elevation where the pine forests dominated.

From the dry southwest, the Langes would have proceeded through Louisiana, Mississippi and Georgia into North Carolina. Hesler’s comment (written in 1975) that Lange visited Penland School in western North Carolina before returning to Denmark, raises the probability that the Langes re-visited the John C. Campbell Folk-school in Brasstown, NC, only some 100 km away from Penland. Unlike the Campbell school, founded along the same Grundtvigian principles as the Danish schools, Penland was, from its beginning in the 1920s, a school of crafts, chiefly weaving, selling the products to supplement the meagre wages of the local population (Craft Revival 2021).

Washington, D.C. (dates surrounding 24 Oct): The Langes (minus Morten) had already seen Washington during their 1927 trip (see above). Their 1931 visit was for very different reasons and now they found a goldmine of mycological activity. The discipline was represented at the US Department of Agriculture, located downtown. The Department had been established in 1862, during the Civil War and within days of the Morrill Act enabling Land-grant Colleges (Petersen 2021). Its functions were divided between two headquarters: research in one location, the herbarium in another. Mycological research centred in phytopathology, as might be expected, which occasionally spun off preserved specimens that travelled to the Smithsonian Institution on the Mall. A considerable roster of plant pathologists worked in the Department. While their research rarely dealt with agarics, they were eager field-biologists and appreciated their local mushrooms. They were (and are) notable and deserve individual sketches.

MycoPortal reports only three collections, from Glen Echo, a suburb and only collection numbers 252–255 were recorded in Lange’s notebooks.

By 1931, Cornelius Lott Shear (1865–1956; Stevenson 1957) had seen numerous changes in the Department since his hiring in 1898 as Assistant Agrostologist under Frank Lamson-Scribner (1851–1938), chief of the Division of Agrostology (later succeeded by Beverly T. Galloway (Petersen 2021). In 1901, the Bureau of Plant Industry was established and absorbed much of the Department’s research personnel. He was a “moving spirit” in forming the American Mycological Society, which existed from 1903–1906, also a charter member of the Mycological Society of America some thirty years afterwards. At the turn of the century, he was an active member of the short-lived Washington Mycological Club, largely composed of USDA workers. While working days, Shear earned his PhD from George Washington University in 1906. In 1923, Shear was made head of the newly-established Division of Mycology and Disease Survey in the Bureau of Plant Industry, formed by consolidation of Divisions of Mycological and Pathological Collections and the Plant Disease Survey. Shear partnered with Bernard O. Dodge (1872–1960) on a seminal paper introducing *Neurospora* as a laboratory tool. All the while, Shear was establishing himself as a scholar of fungus taxonomy and nomenclature, attending successive International Congresses to promote a uniform nomenclature code. Of note was also his partnership with Frederic Edward Clements (1874–1945) on “The Genera of Fungi,” (Clements 1909) which saw both reprinting and revision (Clements and Shear 1951). An appreciation for mycological history saw collaboration with Neil Everett Stevens (1887–1949) on several papers sketching historical mycologists and their activities (Shear and Stevens 1917). Retired in 1935, Shear saw the movement of the mycological herbarium to new headquarters in Beltsville, Maryland in 1941 (Benjamin 1963; Lentz and Lentz 1968), just days before the United States entered World War II. He remained active in the mycological community, including forays, for additional years.

Vera K. Charles (1877–1954; Cash 1956, Rossman 2008) was a transplanted Pennsylvanian who moved to and lived in the District. She attended Holyoke College, but her A.B. degree was from Cornell. Straightaway, she came from Cornell to the Bureau of Plant industry in 1903 and carried on her work in the Office of Mycological Collections and its successors from that date until her retirement from government service in 1942. While her formal occupation dealt with plant pathogens, especially of imported plants, her bulletin on “Mushrooms and other common fungi” in 1913, with Flora Patterson (Galloway 1928; Patterson and Charles 1915; Charles 1929; Rossman 2002) spawned later bulletins on the same subject (Charles 1917, 1931) and another just a few months before the Langes’ visit (Charles 1931; Rossman 2008)). Vera Charles participated in the Cornell Forays and, during Lange’s Washington visit, they were able to do some mushroom hunting in the nearby suburbs (MycoPortal). After her retirement, she spent several winters in Florida, sometimes accompanied by C.L. Shear and Gertrude Burlingham. Hesler (1975) wrote of her: “Miss Charles was a keen collector of fungi. I saw many of her collections at the Mycological Society Foray at Highlands, N.C. in 1933 where she found several which had rarely been found there before.”

Edith Katherine Cash (1890–1992) professed that her only mycological training was a night course at the USDA Graduate School. Nonetheless, her 1912 A.B. from George Washington University in history and languages got her a job at the USDA as a botanical translator in 1913. Over the following half-century, she progressed through the ranks from junior pathologist (1924) to mycologist (1956). Her “day job” in pathology was augmented by a collaboration with Vera Charles on a USDA Bulletin on common mushrooms. She was a vital part of the triumvirate of women mycologists at the USDA; Charles, Patterson and Cash.

According to Batra (1994), Miss Cash’s work on the discomycetes led to an outpouring of monographs of genera. She would have been an eager person to interact with Lange in 1931.

Another USDA mycologist was William Webster Diehl (1891–1978; Lentz 1981). With an M.A. from Iowa State University (1915), he accepted a job at Clemson College for a year, but soon moved to the USDA in 1917, as Scientific Assistant in plant pathology, but spent World War I in Ithaca, New York, learning the skills of the signal corps. In 1919, he returned to the USDA and began a career in plant pathology. He became a lecturer at George Washington University in 1927. In the 1920s, he met Curtis Gates Lloyd and, by Lloyd’s death in 1929, his (Lloyd) herbarium was left to the Smithsonian Institution, but was housed within the USDA. At the AAAS meeting in Des Moines in 1929, Diehl met W.H. Weston from Harvard and soon prepared a proposal to study *Balansia* on grasses at the Harvard Herbaria. The proposal was approved by John A. Stevenson and C.L. Shear and Diehl was on his way. There is no mention of his meeting Jakob Lange in 1931, but, in 1932, Diehl obtained his Ph.D. from Harvard. In 1941, the USDA mycological collections were moved from downtown to Beltsville, Maryland and, during World War II, Diehl was part of the fabric deterioration unit. In 1958, he retired from the USDA. Over the years, he published with the other contemporary mycologists. Had he been in Washington over particular days, he surely would have joined Lange in the field.

Anna Eliza Jenkins (1886–1972) was a New Yorker. Born in Walton, in 1907, she enrolled at Cornell University, where she obtained a B.S. in 1911 and an M.S. in 1912. She was hired by the USDA in 1912, without a Ph.D. and she became an Assistant Scientist in the Bureau of Plant Industry. After some additional studies in Washington DC, she received her Ph.D. from Cornell in 1927. Her professional duties dealt with diseases of imported plant material, but soon she began a series of studies of particular causal organisms. She progressed through the ranks to Mycologist in 1945 (through 1952). Her distinguished career surrounded Lange’s visit of 1931 and she was one of the several working mycologists in Washington at the time of his visit (O’Brien 1975). Along with many others, her collections are housed at the Agriculture Research Service installation in Beltsville, Maryland.

Another of the mycologists hired by the USDA just after the turn of the 20^th^ century was John Albert Stevenson (1890–1979). Born in South Dakota, his early years passed in a fishing village on the shore of Lake Superior in Wisconsin (Farr 1985). His undergraduate years were spent at the University of Wisconsin, where he was taught by M. Freeman, F.C. Stakman, F.E. Clements and C. Bessey. He emerged with intentions of becoming a plant pathologist, attending George Washington University (D.C.), but leaving “ABT” (all but thesis).

Stevenson’s career, like that of several other young mycologists, began with some years in the Caribbean, especially Puerto Rico (Stevenson 1975). As outlined by Farr (1985), there “he also served as librarian, plant quarantine inspector, herbarium curator, editor of Station publications, disbursing officer and assistant to the director”. At the end of World War I, 1918, he left the tropics to work at the USDA as pathological inspector. Subsequently, he occupied several titled positions and, in 1927, succeeded J.R. Weir at the Mycology and Disease Survey, in charge of development of the collections. There he was under the supervision of C.L. Shear, a kindred spirit. This was his position during Lange’s visit in 1931. We could not find any evidence that he joined in the 1931 forays with Lange, but he would have been enthusiastic nonetheless. Over the years, his title changed and he oversaw the move of the National Fungus Collections to Beltsville in 1941. Although he reached mandatory retirement age (70) in 1960, he continued work under a series of emeritus titles.

Stevenson was a collector. Professionally, his collections were of botanical specimens and professional books. As a second penchant, he was a philatelist. The latter two collections were more than significant. Farr (1985) offered the following anecdote: “An avid bibliophile throughout his long career, Mr. Stevenson acquired – frequently with his personal funds – all mycological or phytopathological literature available, including many old, rare and obscure publications. For example, a renowned European mycologist found some rare European books in the NFC [National Fungus Collections] that he had vainly searched for all over Europe and throughout other U.S. libraries (M.A. Donk, letter to Stevenson, March 1970)”.

Stevenson’s major contributions were archival (Stevenson and Cash 1936; Stevenson 1971, 1975).

Brasstown, North Carolina (21 Oct): This stop appears only on the Lange’s itinerary, but must have been sentimental, as it surely was centred in the John C. Campbell Folk-school and perhaps a reunion with Olive Dame Campbell.

New York City (surrounding 26 Oct): Contrary to its inauspicious announcement in *Mycologia*, Lange was sent off from his American visit at an august occasion (Anonymous 1932b). “On October 26^th^ [1931], the newly formed Society [New York Mycological Society] in collaboration with the Torrey Botanical Club and The New York Botanical Garden gave a luncheon in the American Museum of Natural History in honour of Dr. Jakob E. Lange, the Danish expert on gill fungi, who has been spending the summer in the United States. Preceding the luncheon, Dr Lange made an exhibit of several hundred water-coloured drawings of European gill fungi”. As usual, there were last-minute changes in venue and arrangements. A letter from C.W. Dodge to Lange (dated 23 Oct): “We first planned to have the mycological meeting at the Museum Building at The New York Botanical Garden, but later on changed the meeting place to the American Museum of Natural History, where we expect to have luncheon at one o’clock, after which we shall meet in the Academy Room for your talk and exhibit.” In short, the Langes were left to improvise. The cover charge was 60 cents.

Equally low key, Morten (Lange 1996) chronicled their arrival home in Denmark as 5 November.

With their American odyssey over, what were the repercussions?

## ﻿Chapter 4. Intermission

Even as the Langes toured North America in 1931, a drought had descended over an enormous territory from Colorado to Nebraska, a condition observed by Lange (1934). For several years thereafter, drought continued, exacerbated by homesteading and ploughing in areas where rainfall was already only marginal for row crops. High winds produced dust storms month after month, year after year. By 1934, the year of Lange’s (1934) report of his 1931 trip, an estimated 35 million acres of formerly cultivated land had been rendered useless for farming, while another 125 million acres–an area roughly three-quarters the size of Texas–was rapidly losing its topsoil. The worst dust storm occurred in April 1935. News reports called the event “Black Sunday”. A wall of blowing sand and dust started in the Oklahoma Panhandle and spread east, depositing grit as far as New York and ships at sea. Roughly 2.5 million people left the “Dust Bowl” states (a term coined by the press) during the 1930s, one of the largest migrations in American history. As an agricultural educator, Lange must have followed this story from Funen, where such a catastrophe would have been next to impossible.

As the Langes pulled away from the American dock in fall, 1931, political jockeying was already underway for the 1932 presidential election. The Great Depression engulfed the country, and whether at fault or not, Herbert Clark Hoover (1874–1964), the President, was perceived as a non-action leader. His Republican political party shared a philosophy of a small government role, while Franklin Delano Roosevelt (1882–1945), the eventual Democratic candidate, promised a larger role of government in bringing the Depression under control and delivering aid to the people. A year later, Roosevelt won the November 1932 election (both popular and Electoral College) by a landslide, but by law, the election winner in November only took office in March of the following year, in this case, 1933. The American people had to wait for Roosevelt to act. Jakob Lange, after all, was a seasoned politician and surely was interested in the American political process. During the trip, conversations with Americans must have been animated. In Denmark, Lange’s party would side with a large and liberal role for national government and he would have appreciated the Roosevelt programme more completely than that of Hoover.

Upon arrival home, there was little time to digest the kaleidoscope of the trip. By the time the Langes left for their trip, eight instalments of the “Studies” series had appeared and already plans were afoot to publish all his water-coloured mushroom “portraits” together in a more substantial form.

Morten (Lange 1996) summarised the situation: “The first wave of initiative [for *Flora Agaricina Danica*: FAD] came from an invitation to a mycological (and political) study tour to USA in 1931. The encounter with American agarics and American mycologists was very stimulating and the plan for the printing of FAD was under development. The two [Danish] professors with standing in mycology, Ø. Winge and C. Ferdinandsen…took interest in [Jakob’s] plan and started hunting for the necessary economic support …. A hard job in those years of economic crisis”.

Jakob’s duties as headmaster of the Folk-school competed with mycological activity. “Specimens were collected on a morning’s walk through the wood or at one or two Sunday excursions. Each year gave some new paintings” (Lange 1996). Nevertheless, Jakob penned a report, in Danish, to give other Scandinavian interested parties his impressions gathered in America (Lange 1931). For this audience, he pointed out numerous examples of identical mushrooms found on both continents, other very close fits and still others clearly different from both continents.

It took Jakob three years to gather his thoughts into written form for English-speaking colleagues (Lange 1934). He chose to publish them in *Mycologia*, then one of the journals of The New York Botanical Garden and only in the process of being adopted by the fledgling Mycological Society of America as their official print organ (Fitzpatrick 1944). Both of Lange’s reports were acknowledged by “Annales Mycologici” [under “Neue Literatur”, “Annales Mycologici” 33(1): 128. 1935, lists both Lange’s Danish and English reports of his study tour of the US].

It is not easy to extract the core of Lange’s (1934) report. Some problems seem to involve semantics – Lange’s use of an English word which, to an English speaker, creates confused concepts. Some abbreviated excerpts follow. “In other words, is the American fungus-flora chiefly characterized by *identity* [i.e. identical morphology on both continents; in short, the same morphotaxon from both continents], *parallelism* [i.e. European and American basidiomata so close morphologically that separation requires very close observation] or *incongruity* [i.e. European and American basidiomata clearly distinct]?” [sometimes, Lange used the word ‘*diversity*’] (Italics are his).

For Americans, it must have been a challenge to comprehend Lange’s ideas of parallelism. Whether the difference between an American mushroom and its European counterpart was 1% or 4%, distinction was distinction and at what percentage of distinction (and of what characters) did such mushrooms qualify as simply infraspecific variations: forms, varieties or subspecies? At each stop on his tour, Lange was more than willing to exhibit his illustrations, but to appreciate them, the viewer was obliged to make close observation, even to the point of using a hand-lens.

Lange (1934): “Nevertheless, I make bold to state as a preliminary result that the difference between the European and the American fungus-flora is not nearly as great as might be expected from American monographs and floras. If one turns to such publications of recent years, the general impression will be that the proportion between exclusively American species and ‘Europeans’ is about 7:3. But not only in the eastern States but also in the North and West, wherever I gathered a fairly large number of species, I found more nearly the inverse proportion: 70 per cent which were known to me from the European side of the Atlantic, against 30 per cent specifically American. Wherever a European mycologist may go in American woods, he will meet with species familiar to him. Generally, the main aspect of the American fungus-flora will be very ‘European.’ Or to put it more correctly: *The American mycoflora has more of a cosmopolitan stamp than of an exclusively American one*” [Italics are his].

“But what about parallelism in the world of fungi? It goes without saying that the more numerous the truly Americo-European species are, the less will be the chance of meeting with parallels. Still their number seems to be not at all insignificant.

“Altogether the number of true parallels substituting European forms in the Western Hemisphere and not found on the European side of the Atlantic, seems to be rather limited. Evidence is as yet far too incomplete to draw any final conclusions with regard to the problem of parallelism. But to my mind, the facts point in a certain direction: Everywhere in the vegetable and animal kingdom new ‘small species’ or varieties seem to arise by ‘mutation’’ (sudden leaps or sidesteps from the straight path of heredity). If such new forms be equally well - or better - adapted to the natural conditions in the country where they arise, they may establish themselves there or even become the exclusive possessors of the territory hitherto occupied by the parent species. If such new forms have limited means of dispersal, they will become local species. If adapted for wide dispersal, they may gradually spread over unlimited areas.

“… in addition to the direct effect of the climatic conditions, the fungus-flora evidently is influenced by the phanerogamic vegetation, more especially by the presence or absence of certain trees [with] which the particular species is attached. This may account for a good deal of the incongruity of the floras of Europe and America and those of the eastern and western United States”.

Lange (1934): “By far the greatest distinction in the world of fungi is not between a western and an eastern flora, but between southern and northern”.

Description of mycorrhizae had begun in the late 19^th^ century and by 1934, the idea was well-developed, although species-to-species associations and detailed mycorrhizal anatomical studies were still 20 years ahead. Why Lange did not use the term remains obscure.

“But stronger and more lasting than any other impression is the evidence of the wonderful cosmopolitanism of the Agarics” (Lange 1934). This sentiment was not lost on the Americans. John Dearness (1936), the Canadian mycologist, wrote: “In 1931, the veteran Danish agaricologist, Jacob [sic] E. Lange, made a trip across the middle latitudes of this continent for the express purpose of comparing the fleshy fungi of the region traversed with those of Europe. The last statement in his interesting report of the trip … is that his strongest impression was of the evidence of the wonderful cosmopolitanism of the Agarics. He concludes with the question – Who can trace the aerial course of a spore?”

Even as the Langes made their way across North America, there was an “Announcement of formation of the Mycological Society of America at the New Orleans (1931) meeting. Its first meeting will be December 28–30 (1932) in Atlantic City” (Fitzpatrick 1931).

**Figure 20.**
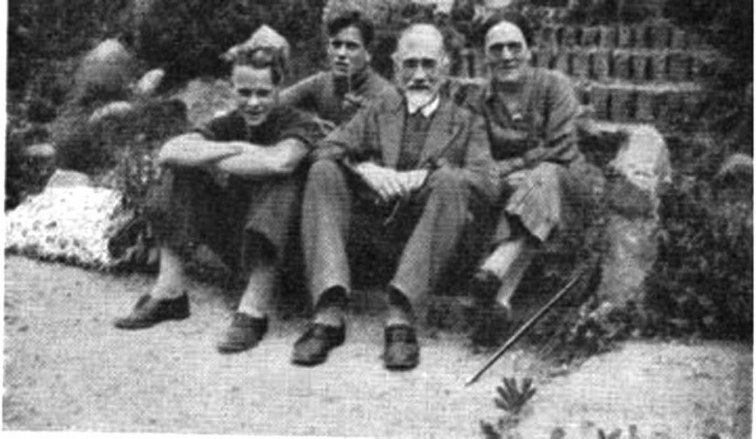
Lange family, about 1934-1935. Left to right: Morten, Jens Jakob, Jakob E., Leila. Source: Morten, 1996.

After a career of teaching, Jakob retired from active administration of the Odense “Husmandsskole” in 1934 (Fig. 20), but his mycological endeavours continued. As Morten (Lange 1996) later related, the first experimental plate of mushroom portraits came off the stone and was approved by the collaborators and supporters. Plans were laid for the appearance of the first volume of FAD the following year.

**Figure 21.**
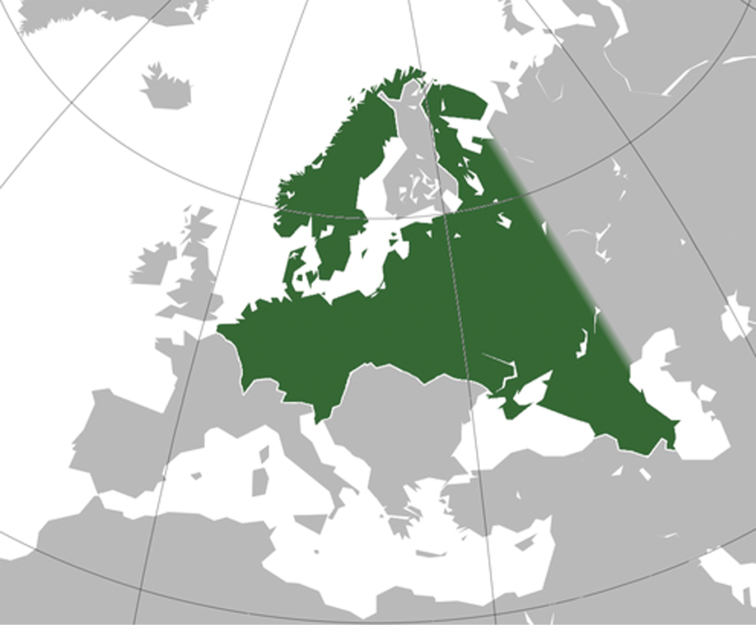
Extent of “*Lebensraum*” as envisioned by Germany in 1934. Source: Wikipedia.

Across Europe, the 1930s had started with a conquered Germany and widespread physical and material ruin. Soon, however, disquieting words and sentiments sent signals of a new Germany (and later, Italy) echoing years of a philosophy dubbed “*Lebensraum*” (“living space”), introduced in the 19^th^ century, but now championed by a rising personality, Adolf Hitler (1889–1945). The idea had two roots: the Aryan people were special (“*Übermenschen*”) while neighbouring people were lesser (“*Untermenschen*”); as the Aryan people needed additional soil through which to support themselves (Fig. 21), the inhabitant population must be eliminated by whatever means necessary. Intermarriage was considered a weakening of the Aryan race and therefore forbidden.

By the mid-1930s, Hitler had taken control of German government and re-armament was underway, officially “swept under the rug” by the conquering powers, themselves tired of war. Austria was gathered under the German Reich as fellow Aryans and “*Lebensraum*” was dusted off, openly threatening Poland and Czechoslovakia. Viewing the gathering clouds, Scandinavia, again, strived to stay, officially at least, neutral.

Meanwhile, Franklin D. Roosevelt took office as president on 4 March 1933 and immediately began implementing programmes to alleviate the economic crisis of “The Great Depression.” In June, he passed the National Industrial Recovery Act (NIRA), which gave workers the right to organise into collective representative organisations - in short, unions. Its most significant passage was:

“Employees shall have the right to organize and bargain collectively through representatives of their own choosing and shall be free from the interference, restraint or coercion of employers”. Although the NIRA was ultimately deemed unconstitutional by the Supreme Court in 1935, it was immediately

replaced by the Wagner Act legislation (the National Labor Relations Act) with the same intent and workers were encouraged to join and strengthen unions and other such organisations. Expressly excluded from the union movement were agricultural workers – but the law’s overall motive was close to the heart of Jakob Lange.

The “mid-term elections” (held in the midst of the presidential term of office) of 1934 might have reflected the “radical upheaval sweeping the country,” as the Roosevelt administration won the greatest majority either party ever held in the Senate and in the House of Representatives, 322 Democrats to 103 Republicans. Coincidentally, the strength and independence of the Executive Branch was expanded as never before.

The year 1935 brought the 10^th^ in Lange’s (1935b) “Studies” series, this time a floristic monograph of *Cortinarius*. Taxonomy within the genus, Lange wrote, depended heavily on colours, but written descriptions were difficult to understand when colours were named, especially when non-colorimetric references were made such as “pecan” or “cream”. In other instances, the same colour was described with different terms, such as “lilac”, “lavender”, “violet”, “purple” or “bluish”. An accurately coloured “portrait”, however, by-passed such confusion. Lange was aware of Kauffman’s (1918) treatment of *Cortinarius* for Michigan or eastern United States, who cited Ridgway (1912), but did not use Ridgway’s terminology. Lange’s (1935b) sole plate was special for the instalment, not reproduced later in FAD. It is interesting that Kauffman’s (1932) posthumous offering on *Cortinarius* for “North American Flora” was not cited by Lange (1935b); perhaps this series was not part of Lange’s library.

An unexpected reader was John Dearness (1935) in Canada. “Professor Jacob [sic] E. Lange has recently published a monograph in English of the Danish Cortinarii in which he keys and describes 120 species, being about twenty more than half the total number of North American species described in the N[orth] A[merican] Flora. He accepts the six Friesian subgenera and keys them separately dividing each subgenus into two subsections. The quality of the keys is always proved by their use; however, a careful reader of these six examples will probably admit the author’s claim that they are so clear and plain that “a novice may be able to follow their lead without getting side-tracked on the road”.

**Figure 22.**
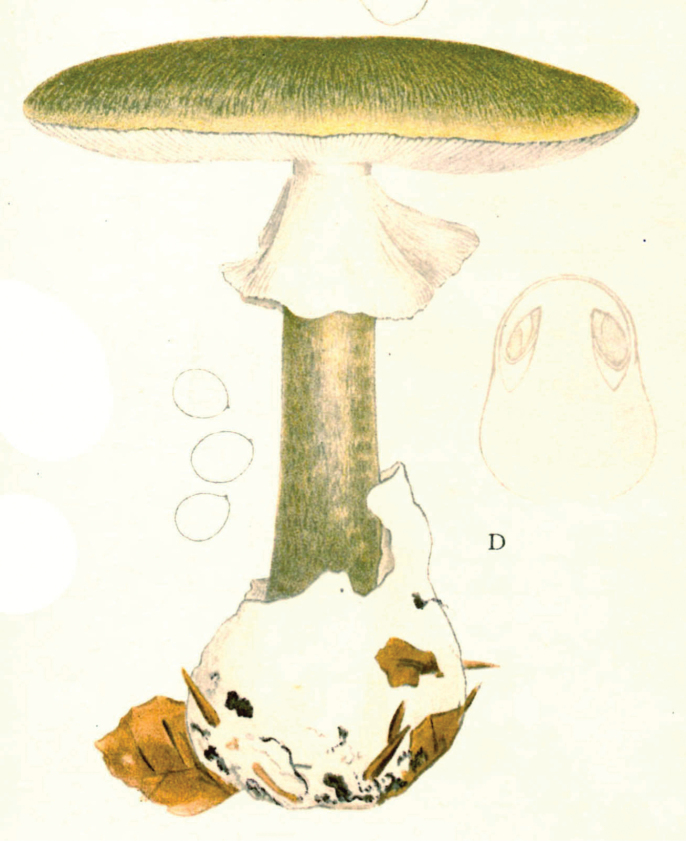
Detail from “Flora Agaricina Danica”, Vol. 1. Plate 1D. *Amanitaphalloides*. Original 11 × 13.5 cm.

**Figure 23.**
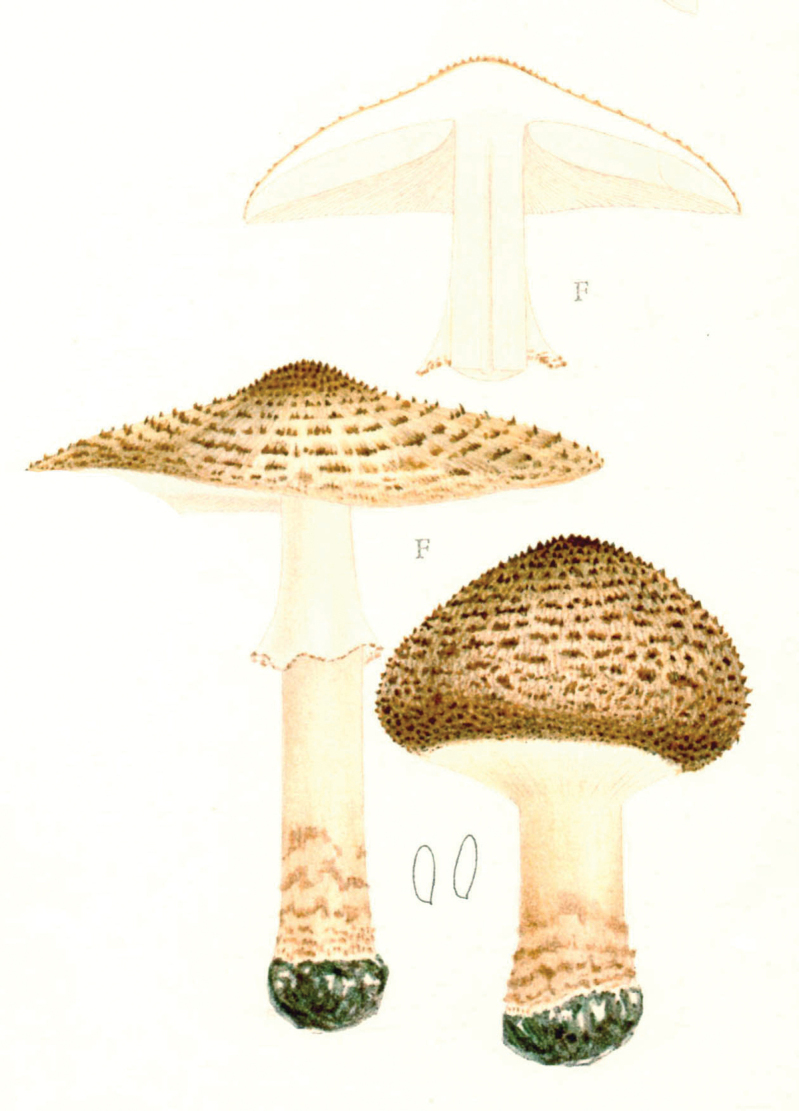
Detail from “Flora Agaricina Danica”, Vol. 1. Plate 10F. *Lepiotaacutesquamosa*. Original 11 × 14.5 cm.

**Figure 24.**
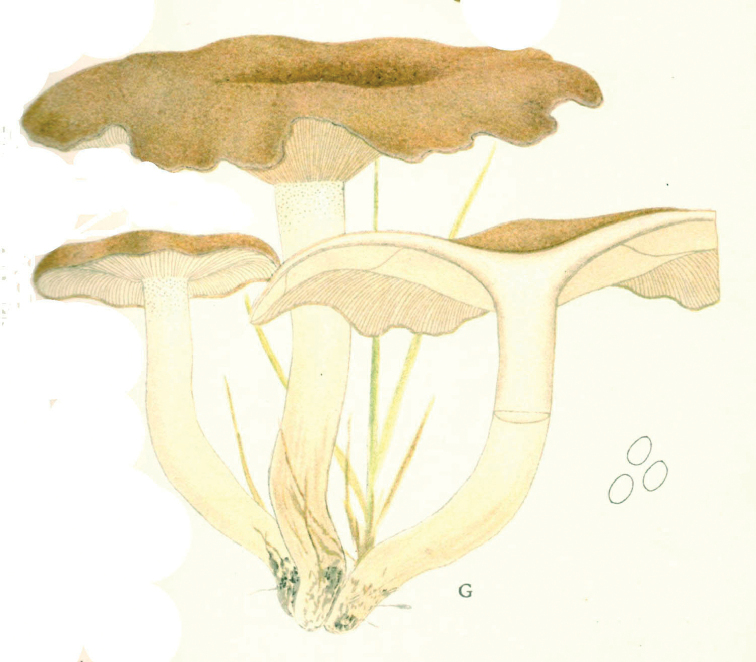
Detail from “Flora Agaricina Danica”, Vol. 1. Plate 40G. Clitocybeaggregatevar.ovispora. Original 12.5 × 14.5 cm.

According to Morten (Lange 1969), no. 10 in the “Studies” series appeared in July 1935 and, in November, the first part of Volume 1 of FAD was completed (Figs 22–24). There must have been cause to celebrate – plans for the FAD series had been laid well in advance. However, a close reading of Morten’s report would indicate that several other persons were in charge of various tasks in the production and support of FAD. “Poul Larsen, Ferdinandsen and Winge have their great part in the production of the work through their pressure to secure the economic basis and in the primary development of the printing standards to be met. N.F. Buchwald served as secretary, helped to see the pages through the press, completed the index and organized the distribution. The production was in itself a very excellent job. The technical work of transferring the pictures to the lithographic stones was done by one highly skilled man (O.R. Poulsen) and as much as ten colours were generally needed to make up the final print”. Even Lange’s inked images of cystidia, basidia and spores were faithfully reproduced in lithography. The tasks remaining for Lange were to write the text and provide the images: Morten wrote “[J.E.] Lange was no friend of long descriptions...” and Latin diagnoses only became required for nomenclature in 1935, just in time to plague the author. The plates were montages of Lange’s original “portraits” on glossy paper, while the text was on quarto opaque paper of large print-face. A separately paged general introduction was culminated, but added as an introduction before Volume I, consisted of Latin diagnoses of new taxa and index of all illustrations by genus and by volume/plate. Few descriptions bore detailed geographic information; these details were included in the two notebooks mentioned by Morten (Lange 1969) and now at the Natural History Museum of Denmark, Copenhagen. Soon, in the middle of March (Lange 1936b), the second part of Volume I was produced. FAD took its position amongst contemporary and preceding illustrated works on European agarics (Petersen and Hughes 2017; Krieger 1927).

The appearance of FAD was the result of clear-eyed business collaboration, not only the realisation of a mycological dream. Years previously, Lange had already had a mycological disagreement with Ferdinandsen and Winge (1924–1925), two of the chief collaborators on FAD. It concerned proposal by the latter two of a new species of *Russula* (*R.solaris*), which Lange considered only *R.raoultii*, already described by Quélet and already known from the Danish mycoflora. Later (Studies XII: 103, 1938a), however, Lange acknowledged the new species. The three remined friends and colleagues throughout.

Such an auspicious start was bound to be recognised by the mycological community, both European and American. In the German journal, “Annales Mycologici” (1935 [1936], 34(2): 265) both number 10 of the “Studies” series and Volume I of FAD were simply acknowledged under “*neue literatur*”.

In Paris, Roger Heim (1936) inaugurated the new journal, “Revue de Mycologie”, with a review of Volume I of FAD. In part, he wrote (translation from French HK): “The world’s botanical authority, the author of ’Studies of the Agarics of Denmark’ ... in fact, [makes] this publication of particular importance. ... It is not only a matter of providing a very new system for the agarics, but the masterly qualities of the author – his sincerity, his clarifying spirit, his fine scientific sense – allow consideration of his personal concept for each species as a defining statement.

“He seeks – within the genera – to group the taxa in a logical way by using simple and clear distinctions. Mr. Lange is a master of this game and it is perhaps especially this charm from his publications which is retained and reappears in an agreeable way. ... Lange always insists on the precision of the descriptions by adding twigs, dung and other precise biological characters [to the illustrations], rarely recognized by other authors.

“All those who regretted that these essays were applied to a small number of genera will be fully satisfied when they learn that this Flora will be the generalization of the author’s previous observations, to which are added excellent watercolours in the sense that they are those that mycologists may desire; without too deliberately artistic effects, but scientifically complete and rigorous”.

As Volume 1 dealt with *Amanita* and *Lepiota*, Heim discussed these genera and their infrageneric taxonomy, noting differences between Lange’s arrangement and that of contemporary French authors.

“We sincerely hope that the real difficulties in these times [the Great Depression and the build-up to WW II] will not delay the publication of such a precise work, which simultaneously brings honour to the knowledgeable author and to two Danish groups which have taken on this enterprise”.

Heim (1931) was no novice in such publications. His treatise on *Inocybe*, for instance, had numerous colour plates quite similar to those used for FAD. Lange (1917) had also investigated the genus. From France, Robert Kühner (1935) also published his comprehensive treatment of *Galera*, citing unpublished coloured illustrations by several workers, including Lange.

Virtually simultaneous with Lange’s first instalment of FAD came a mushroom book from the United States, illustrations from which rivalled those by Lange. Louis C.C. Krieger had worked with W.G. Farlow (Petersen 2019) in producing watercolour figures of New England fungi, but in 1935, Krieger was mycologist of the State of New York, in the succession of Charles H. Peck. His “Popular Guide to the Higher fungi (Mushrooms) of New York State” (Krieger 1935) and its reprinted successor, “The Mushroom Handbook” (Seaver 1936) were illustrated by a combination of photographs (not taken by Krieger) and watercolours. The quality of the latter was quite high.

The year 1935 also saw a paper on American *Mycena* taxa by Alexander H. Smith. Smith had started his graduate work under Calvin H. Kauffman at the University of Michigan, but had to switch to E.B. Mains when Kauffman was incapacitated. Under Mains’ influence, Smith obtained his PhD in 1933, with a dissertation on two-spored forms of *Mycena* in North America. He had ample reference to Lange’s 1914 study of the genus in Denmark (Lange 1914). Lange’s “Studies” series parts I and V appeared in Smith’s bibliography. Smith’s 1935 paper was the first in a series of five (Smith 1935a, b, 1936, 1937b, 1939).

Now, some twenty years after Lange’s study, Smith wrote: “No comprehensive treatment of the genus *Mycena* giving proper emphasis to both microscopic and macroscopic characteristics has been published for the North American species. Atkinson had such a study in mind and, at the time of his death, he accumulated considerable information toward that end. Unfortunately, however, it was never published. Kauffman was engaged in a similar study in 1929, but withheld it from publication because he felt that it was incomplete. As a result, Kauffman’s treatment of the genus in ‘The *Agaricaceae* of Michigan’ and that of Beardslee and Coker (1924) on the species found in North Carolina are our most reliable sources of information.

“Very little authentic European material has been available for comparison and it has been necessary to rely on published descriptions and figures”. Lange (1934) acknowledged as much.

An important “*déviation obligatoire*” is necessary. In 1922, a small paper appeared in a new journal from Germany, “Zeitschrift fur Pilzkunde” and was authored by a young Rolf Singer (1906–1994; Singer 1922). It introduced the man who would become the leading agaricologist of the 20^th^ century, not only for Europe, but worldwide. Over the next few years, he seemed to deal especially with *Russula*, but already had greater horizons in mind (Mueller and Wu 1998). In 1936, some 50 publications later, Singer (1936) introduced his overall taxonomic scheme for the agarics. His productivity and taxonomic breadth would eclipse all other individuals and his very presence would influence all writings on the subject. His new system, presciently, was written in Leningrad, the temporary home of Rolf and Martha Singer, newly-weds, reflective of the times.

In February, 1936, Hitler convinced the Chancellor of Austria to allow Germany to control the Austrian economy, citing the unity of Aryan peoples. The next month, German troops marched into the Rhineland; numerous Germans saw difficult times ahead and felt obliged to emigrate from their fatherland.

January 1937 saw the 11^th^ instalment of the “Studies” series (Lange 1936a), this one dealing with several genera, but without a coloured plate. Two months later, the second volume of FAD appeared. In both instances the technical tasks were no longer new and especially the plates for FAD Volume II, planned well in advance, were produced almost routinely, but always under Lange’s eyes. By mid-year, the publication had arrived in Michigan, where Alex Smith (1937a), now on faculty, wrote a review.

“… The first volume [of FAD] appeared in 1935 and 1936. Although the author [Lange] has confined himself to the Agaricaceae of Denmark, his work is indispensable to critical students of the family in the United States and Canada. Many of the curious and unusual species which Dr. Lange has discovered are widely distributed and are to be found both in eastern North America and along the Pacific Coast. The work is outstanding because all the species recognized in the Danish flora are described and illustrated. An unusually high degree of accuracy has been obtained in depicting and reproducing the natural colours and fine details in each species. There are keys to all the species and brief descriptions which emphasize the characters the author considers important”.

Smith summarised the taxonomic outline of the two volumes. He then pointed out a few nomenclatural shortcomings. “Such errors as these are practically inevitable in a group where little authentic material exists and the literature is widely scattered. They do not detract materially from the value of the work as a whole. There is little doubt that Lange’s Agaricina Danica [sic] will always remain one of the outstanding contributions in Agaricology.”

A few months after the appearance of Smith’s review, he and Marcel Josserand (Josserand and Smith 1937) collaborated on a paper which centred on Lange’s (1934) English language report on his trip to America. Josserand, based in Lyon, was already a leader in French mycology. Lange’s report had dealt at some length on his own observations and his conclusions about agaric distributions. The three categories he (Lange) sketched for transatlantic mushroom distributions were “identity”, “parallelism” and “incongruity” (q.v. above).

The authors wrote: “…Lange in his comments on agarics in North America discussed briefly the existence of “parallel species” in North America and in Europe. He also re-emphasised the view, which many American mycologists have long held, that, in reality, the North American fungous flora and that of Europe are characterized by the presence of a larger number of species in common than the published floras indicate. The task of accurately determining the true synonyms and the recognition of ‘parallel species’ is a very delicate one and involves a careful study of the variations of each species not only in each country, but in various regions of the same country as well as in the same locality over a period of several seasons. The co-existence of a striking character in a European and an American species does not suffice to justify the identification of the one with the other. In order to pronounce them synonyms, it is necessary to have a perfect superposition of the two series of characters. If there is the slightest doubt, it appears to be wiser not to place them in synonymy for we believe that it is incomparably less serious and less confusing to permit the existence of two names for the same plant than to designate a mixture of two species with a single name”.

It is not surprising that Josserand & Smith should single out *Mycena* in their criticisms. In 1935, Smith was two years out of graduate school at University of Michigan, with his dissertation on two-spored forms in the genus in North America. Lange’s English report on his 1931 trip had appeared only in 1934 and the paper with Josserand was Smith’s first chance to comment on Lange’s remarks on biogeography of *Mycena*.

Time has blurred precisely how much Lange’s 1931 trip to America – person-to-person interaction and/or probable reference to the “Studies” series (FAD had not appeared in 1931) – influenced the work of American agaricologists, but the Americans sometimes mentioned Lange explicitly or cited his publications in their bibliographies. These references are of help in telling Lange’s story, but are difficult to search out. The following are examples.

Lange may have stopped in Seattle or not, but his influence was felt nonetheless. Hotson and Stuntz (1938) mentioned Lange’s (1934) report on his trip and Lange’s “Studies” XII; 1938) in their paper on *Agaricus* on the west coast. Lange’s mention of the sterile cells at the gill edge (cystidia) were mentioned and illustrated.

Hotson and Stuntz (1938) stated: “The genus is well represented in the Pacific Northwest, but when one wishes to identify collections made in this region, he faces an obstacle in the lack of available information concerning the local flora and also in the fact that some of the species occurring here may also be more closely allied to European forms than to those found in the eastern United States”. This certainly has the ring of Lange’s (1934) sentiments and also those of Kauffman (1918).

Lange’s visit with S. M. Zeller in Corvallis, listed in his itinerary and vouchered by specimens included in MycoPortal, was reflected by Zeller (1938). Under *Armillariarobusta*, Zeller drew attention to the illustration by Lange (1935b). A new species, *Lepiotaoculata* Lange & Zeller, collected at Hemlock, Oregon, was described and *Lepiotacygnea* Lange, pointed out by Lange (1934), was reported as collected during Lange’s stay.

A year after Volume II of FAD (Lange 1937), March 1938, brought the appearance of FAD III (Lange 1938c) and, in October, the last instalment of Lange’s (1938a) “Studies” series. The former was reviewed by Smith (1938), who, in a somewhat perfunctory discussion, furnished an account of the taxa and infrageneric taxonomy of *Cortinarius*, *Inocybe*, *Hebeloma* and *Pholiota*.

## ﻿Chapter 5. 1939

Jakob’s (1938b) recollections of his American travels were written a year before his final trip to America. Fortunately, Morten’s account of the 1939 trip, although brief, is precise and accurate.

From the American side, the year opened with some equivocations on that year’s foray time and location (Mains 1939). By March, dates could be posted (Linder 1939b). “The Mycological Foray will be held in the Great Smoky Mountains National Park, but with headquarters in Gatlinburg, Tennessee. In view of the fact that the meeting will be held from August 17^th^ to 20^th^ inclusive, during the tourist season, it is advisable that those planning to attend make reservations early at one of the following places, all within a radius of a mile: [a list of about 15 hotels & cabins]”. Although we know of no evidence of an American invitation to Jakob Lange to join the foray, one must have been transmitted, perhaps from John Dearness (see below) or perhaps C.W. Dodge. Such an invitation would have coincided with other, non-mycological proposed appointments.

From Morten Lange (1996): “My father invited me on a new America trip in the summer of 1939. He had received an invitation to a mushroom congress in Tennessee and to give a couple of lectures about [Danish] agricultural education in Washington and Pennsylvania. It looked as a new exciting trip. I should accompany as a ’qualified companion’ on a five-week, somewhat frenzied, trip”. (Wartime in Europe limited categories of persons embarking across the Atlantic, especially from Great Britain. Only “qualified companions” were allowed to accompany credentialled passengers) (Lange 1996).

The years leading to Lange’s 1939 trip were a chronicle of turbulence. From the United States, the Roosevelt “New Deal” was not without its detractors and, on the European side, there was little but threatening news.

As early as 1922, Italy had devolved from monarchy to fascism. Benito Mussolini (1883–1945) saw his paramilitary descend on Rome and was rewarded with the prime minister’s appointment. On 3 January 1925, he asserted his right to be supreme ruler and declared himself dictator of Italy.

A decade later, following Italy’s 1935 invasion of Ethiopia, Germany was the second country to recognise Italy’s legitimacy there. Over subsequent years, Germany’s industrial might and population propelled Hitler to eclipse Mussolini. Both Hitler and Mussolini sided with Francisco Franco (1892–1975) in the Spanish Civil War (1936–1939), with Mussolini providing 50,000 troops. In 1937, Italy left the League of Nations in solidarity with Germany. Parenthetically, 1935 saw Rolf Singer and his wife forced to flee Spain for Paris.

Perhaps a useful means of communicating the events of the period is through a chronology as follows:

12 March 1938: The *Anschluss*, the annexation of Austria into Greater Germany, began as a large contingent of German troops entered Austria. The *Anschluss* ostensibly reunited ethnically similar Aryan cultures and many Austrians welcomed the German soldiers.

March 1938: Volume III of FAD appeared in the midst of threatening times (Lange 1938c).

30 September 1938: In Munich, Germany, representatives of the victorious countries of World War I ceded to Germany the Czech Sudetenland – the Czechoslovakian borders on its west, south and north. The territory included the major defences of Czechoslovakia against Germany and Poland, so the Czechs were rendered defenceless. Soon, the remaining Czech territories and the Hungarian border land were also awarded to Germany. The British Prime Minister, Arthur Neville Chamberlain (1869–1940), upon arrival home after the Munich Conference, declared its agreements as “Peace for our time”.

In the context of the post-war (WW I) economic depression of the 1930s, the German National Socialist Party (“Nazi”) gained popularity in part by presenting Jews as the source of a variety of political, social, economic and ethical problems permeating German society. The Nazis used racist and also medieval social, economic and religious imagery to this end. Inspired by theories of racial struggle, Hitler preached the intent of the Jews to survive and expand at the expense of Germans. The Nazis, as the governing party, ordered anti-Jewish boycotts, staged book burnings and enacted anti-Jewish legislation. In 1935, the Nuremberg Laws defined Jews by race and mandated the total separation of “Aryans” and “non-Aryans”. These measures aimed at both legal and social segregation of Jews from Germans and Austrians. On the night of 9 November 1938, Nazis and their sympathisers destroyed synagogues and shop windows of Jewish-owned stores throughout Germany and Austria in what became known as “Kristallnacht”.

Simultaneously, people of German ancestry living abroad were encouraged to form citizens groups both to extol “Germanic virtues” around the world, but also to lobby for causes helpful to Nazi Party goals (Atlantic 2021). In the United States, the “Amerikadeutscher Volksbund” or German American Bund was formed in 1936 as “an organisation of patriotic Americans of German stock” (Atlantic 2021; Bund 2021). The Bund eventually grew to a membership in the tens of thousands. On 20 February 1939, the Bund held an “Americanisation” rally in New York’s Madison Square Garden, denouncing Jewish conspiracies, President Roosevelt and other controversial subjects. The rally, attended by 20,000 supporters and members, was protested by huge crowds of anti-Nazis (Bund 2021). As war broke out and German allegiance forced difficult choices, the German-American Bund fell apart, many of its assets were seized and its leader was arrested for embezzlement and later deported to Germany.

October 1938: The 12^th^ and final instalment of Lange’s (1938a) “Studies” series appeared. As planned, the summary of Danish agarics had been completed.

March 1939: German army occupied Prague, Czechoslovakia, in clear violation of the Munich Agreement.

31 March 1939: In the face of the perceived German threat, Britain and Poland announced a mutual assistance pact in case of military threat, but the treaty was not signed until August. From the British Prime Minister: “... [I]n the event of any action which clearly threatened Polish independence and which the Polish Government accordingly considered it vital to resist with their national forces, His Majesty’s Government would feel themselves bound at once to lend the Polish Government all support in their power. They have given the Polish Government an assurance to this effect. I may add that the French Government have authorised me to make it plain that they stand in the same position in this matter as do His Majesty’s Government”. On 13 April, the assurance was extended to Greece and Romania. Formal signing was accomplished on 25 August.

May 1939: Germany demanded a non-aggression pact with the Scandinavian countries. Sweden and Norway rejected the idea, but Denmark accepted, which created a major physical obstacle for the Britain/Poland alliance.

Early May 1939, Volume IV of FAD appeared in Denmark (Lange 1939a, 1969). Considering the geopolitical context, in retrospect it is a wonder that the raw materials for the production - paper, ink, lithograph stone etc. - were available.

22 May 1939: Italy and Germany signed the “Pact of Steel” officially creating the Axis powers. (Japan would join in September of 1940 with the signing of the “Tripartite Pact”).

12 August1939: The Langes, father and son, pushed off from England for New York “on a fast boat,” arriving on 18 August and immediately boarded a train headed south towards Tennessee and a “mycological congress.” Morten was impressed that they disembarked at 6:30 pm Daylight Savings time and boarded the train at 6:30 pm Standard time. Daylight Savings Time had been instituted during World War I, but afterwards had been scrapped. Roosevelt attempted to resurrect it, but it was rejected again, except for a very few States and large cities, one of which was New York. The railroads ran on Standard Time and this would persist all the way to Tennessee.

**Figure 25.**
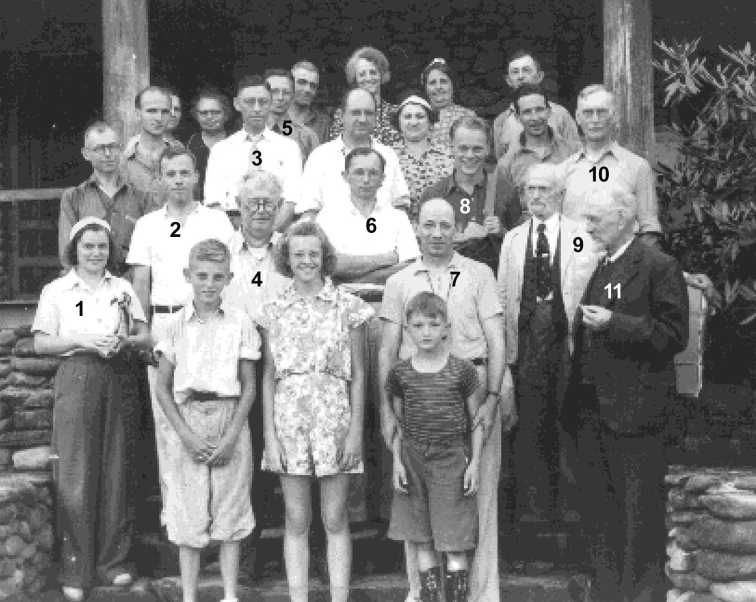
Mycological Society of America Foray, 1939, Gatlinburg, TN **1** Helen Smith **2** David Linder **3** L.R. Hesler **4** Robert Hagelstein **5** Arthur Stupka, Smokey Mountains National Park biologist **6** Alexander H. Smith **7** L.O. Overholts **8** Morten Lange **9** John Dearness **10** C.L. Shear **11** J.E. Lange. Source: L.R. Hesler.

**Figure 26.**
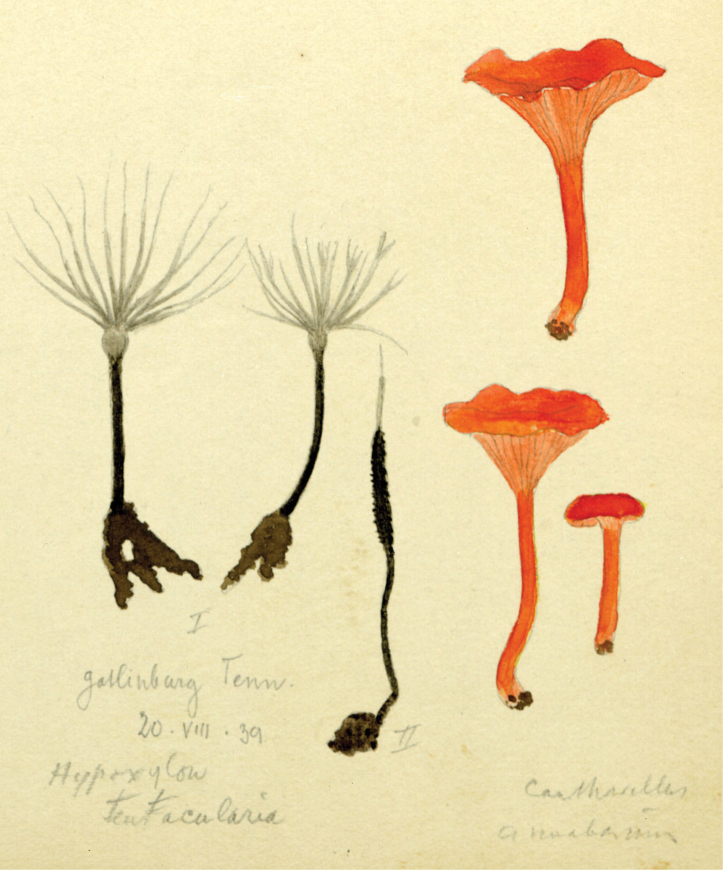
Lange aquarelle sketch dated 20 August 1939 (Gatlinburg, TN). Left: *Hypoxylon* “*tentaculus*”. Right: *Cantharelluscinnabarinus*. Courtesy Natural History Museum of Denmark, Copenhagen.

**Figure 27.**
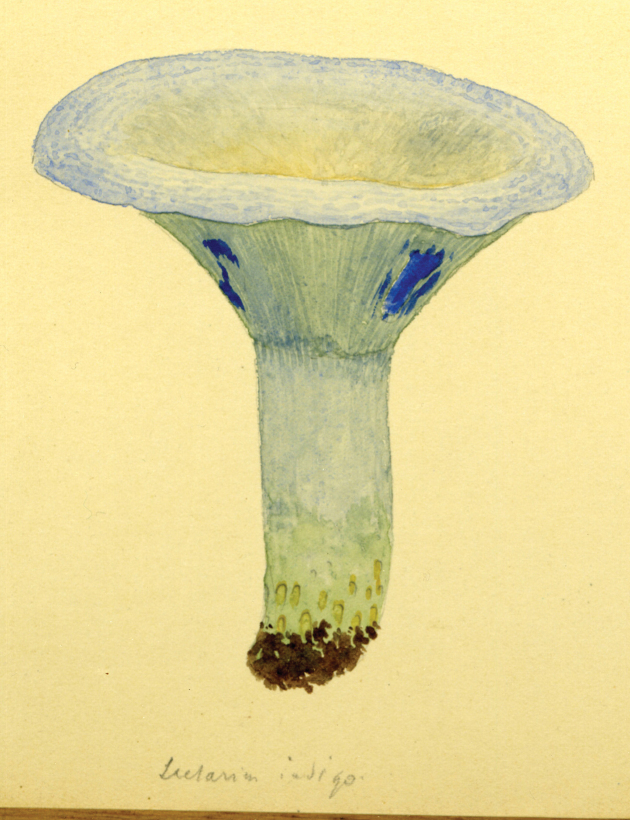
Lange aquarelle sketch dated 20 August 1939 (Gatlinburg, TN). *Lactariusindigo*. Courtesy Natural History Museum of Denmark, Copenhagen.

It must have been no sooner than 19 August that the Langes reached Gatlinburg, Tennessee and the 1939 foray of the Mycological Society of America. The foray was scheduled for 17 – 20 August, so the Langes had no more than a few hours with the large contingent of mycologists – just long enough to be caught in a group photo (Fig. 25). Morten later wrote (Lange 1996): “Among them [was] Alex H. Smith from Michigan, whom I agreed to visit soon for a study period. It was only realized later, in 1947”. By mutual agreement, that study period was concerned with *Coprinus*, in this case, the *C.ephemerus* group (Smith 1948; Lange 1952; Lange and Smith 1953). One can only imagine conversations between Jakob Lange and Alexander Smith, who had taken Jakob to task over their common interest in *Mycena*. Although time was severely limited, Jakob managed to make several sketches dated 20 August (Figs 26, 27).

**Figure 28.**
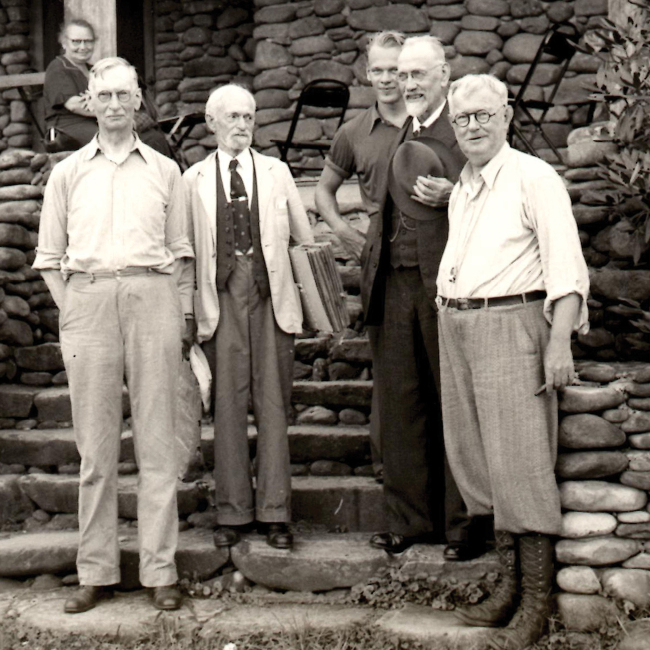
Mycological Society of America Foray, 1939, Gatlinburg, TN. Left to right: C.L. Shear, John Dearness, Morten Lange, Jakob Lange, Robert Hagelstein. Source: L.R. Hesler.

It is quite possible that a small group of mycologists remained in Gatlinburg for extra collecting and conversation. A second photo might suggest that amongst them were C.L. Shear, John Dearness and Robert Hagelstein (Fig. 28).

23 August 1939: Meanwhile, secretly, the USSR was negotiating with both Britain and Germany. The deal with Germany promised a Soviet “Sphere of Influence” including eastern Europe, the Baltic States and Finland. Joseph Stalin was attracted to this much better deal from Hitler and the USSR signed the (Vyacheslav) Molotov-(Joachim von) Ribbentrop Pact.

Morten (Lange 1996) wrote: “After four days of advanced mycology, there was room in the calendar for a fast visit to Mrs. Olive D. Campbell’s high-school in North Carolina and accordingly private mushroom trips”. This would mean about 23 August (= 4 days after arrival in Gatlinburg on 19 August). Whether Mrs. Campbell was present is doubtful, since the Langes visited her summer residence on Nantucket some days later. Nonetheless, the visit to Brasstown must have been a sentimental journey, since Jakob (with Leila) had visited there in 1927 and again (this time with Morten) in 1931. The school had built and acquired several new buildings, but perhaps more importantly, it had become the centre of a farmers’ cooperative, a dairy cooperative and a growing network of crafts people. In each case, the power and leverage of individual participants had been magnified. Activities did not take the place of public education, but introduced new methods and communication, all echoes of the “Husmandsskole”. Jakob was able to execute a small number of sketches, including one of *Calostoma*, but not the one discovered by Jakob in 1927.

25 August (probably): Morten wrote (Lange 1996) “And then there was time for lectures in Washington and at Penn State College”.

In 1931, the Lange family had visited Washington, where they spent time in the field with USDA workers. Most of those folks were still in their jobs and reunions were probably pleasant. At least Cornelius Shear and Vera Charles had been at the Gatlinburg foray, lending additional familiarity.

Morten described the lectures as agricultural, not mycological and used the plural, but nothing specific is known about them. One person already known to Jakob was Henry Agard Wallace (1888–1965), then Secretary of Agriculture under Roosevelt and slated to be Roosevelt’s Vice President after the 1940 election.

Sounding much as though it could have come from Grundtvig’s pen, “The Farmers’ High School” was established in Pennsylvania in 1855, seven years before the land-grant act was passed. The implication, of course, was that agriculture already played a significant role in the mission of the school, reflecting the rural agrarian demographics. The first graduating class of 13 males in 1861 was the first such class at an American agriculture institution. Just as the Grundtvigian schools in Denmark, agricultural programmes at “Penn. State” included numerous short courses so students could receive education and immediately apply their new knowledge to their livelihood.

In terms of mycology, at least three faculty members were on board in 1939. Frank Dunn Kern (1883–1973), expert in rust fungi, was not only Head of the Department of Botany, but also Dean of the Graduate School. George Lorenzo Ingram Zundel (1885–1950) was plant pathology’s extension representative to the farming public. Lee Oras Overholts (1890–1946) had already published several papers on polypores and had served as President of the Mycological Society of America in 1938. He had just attended the Gatlinburg foray the previous month. James Whaples Sinden (1902–1994) was becoming well-known for his development of “grain Spawn” in the mushroom industry.

In the 19^th^ century, caves in and around Chester County, Pennsylvania, had become home for a thriving mushroom growing industry, businesses often owned and run by immigrant families. With the advent of mechanical aids, especially in ventilation, the industry went above ground and, after World War II, the area produced the lion’s share of mushrooms in the United States. Pennsylvania State University evolved an advisory role to the mushroom farmers’ association, later adding a research laboratory and short courses of its own to serve as extension, under the leadership of Leon R. Kneebone (1920–2020; Kneebone 2021).

1 September 1939: Lange (1996): “The 1^st^ of September we returned to New York, [just as] the war broke out in Europe. Father’s prophet, Henry George, had been dead for a long time, but his daughter – Anne George de Mille [1878–1947; married to William, brother of the film magnate C(ecil) B. de Mille] gave an elegant private dinner and we made sure to change [our reservations] to the Swedish ship ‘Gripsholm’”.

On that very day, Hitler, convinced that Britain would not intervene and assured that USSR would not interfere, sent troops into Poland in the face of the Munich Conference agreement and the mutual assurance pact between Poland and Britain. Within days, the USSR entered Poland from the east and the country essentially disappeared.

Great Britain immediately declared war against Germany. This was not a singular event, for British agreements with its dominions – Australia, Canada, South Africa, New Zealand and other colonies such as India and African possessions – brought them also into the War, both in Europe and across the Pacific.

September 1939: Denmark’s reaction to these tumultuous events was to declare neutrality, as it had been for World War I. Danish government and society continued to function more or less normally, but always “looking over their shoulders” as their southern neighbours killed one another. The southern areas of Schleswig and Holstein were again in play. The Germans, contrary to their other incursions, considered the Danes a kind of Aryan and therefore qualified for less harsh treatment.

A stop in New York City would not have been complete without a visit to Columbia University to see John Sidney Karling (1887–1994; Anonymous 1996; Karling 2021), by then a full professor. Karling had earned his PhD there under R.A. Harper and remained on faculty. Equally, if time allowed, a side trip to the New York Botanical Garden would have been worthy, especially to meet with Fred Jay Seaver (1877–1970; Rogerson 1973; Seaver 2021), the Editor in Chief of *Mycologia* and Curator of the Herbarium.

Lange 1996: The change of transatlantic vessels “...gave us a few extra days for a fast visit on Nantucket to Mrs. Campbell’s summer residence. The Island is situated further at sea than Martha’s Vineyard and is finer. Our host had two original houses built for whaling captains”. Mrs. Campbell was now 57 years old; it had been 14 years since she had first met Jakob in Denmark.

The evident reason for the change in transatlantic schedule were widely circulating reports of German “U-boats” (submarines) plaguing shipping on Atlantic seaways. On the advice of American hosts and colleagues, bookings were changed to the “Gripsholm,” a vessel of the Swedish-American Line, which sailed under the flag of neutral Sweden. Morten (Lange 1996) described: “... and then sailing with a spotlight on the [Swedish] flag, up to the Faroe Islands [southeast of Iceland and due north of Great Britain] and inside the coast in the Norwegian archipelago from Ålesund [a town on the Norwegian coastal islands] to Göteborg [on the Swedish coast just south of Norway]. The whole development in the war [was followed] through American newspapers and as telegrams to the ship. It was tough!”

20 September 1939: The Lange men arrived home. With this, the 1939 North American visit ended, but the tension and stress of the return voyage was only enhanced by the turmoil on all sides of Denmark. Nevertheless, it must have been welcome to return to retirement at the Odense “Husmandsskole”.

## ﻿Chapter 6. Epilogue

17 December 1939: As the year wound down, in Berlin, the decision was made to occupy Denmark. Within days, German troops occupied Copenhagen, violating Denmark’s neutrality. The Germans had already planned to use northernmost Denmark as a jumping off spot in an invasion of Norway.

Morning, 9 April 1940: Germany declared Denmark a protectorate as it began the invasion of Norway. Norwegian resistance was valiant, but failed and Norway capitulated on 10 June. With Denmark and Norway in its pocket, Germany controlled commerce over the Baltic Sea.

9 April 1940: The Danish envoy to the US signed an agreement under which the US would defend Greenland, a Danish protectorate. The agreement also provided the US an opportunity to erect military bases on the Island. This arrangement moved the US closer to the war without being directly involved.

12 April 1940: With Danish permission, Britain peacefully invaded the Faroe Islands, another Danish protectorate and fortified them.

10 May 1940: The United Kingdom invaded Iceland as a pre-emptive strike, eventually turning it over to the USA in July 1941. Occupation of the Faroes, Iceland and Greenland were all attempts to preserve the northern transatlantic waterway.

Germany’s occupation of Denmark and invasion of Norway convinced Mussolini that Hitler would win the war. In continental Europe, neutral Holland (15 May 1940) and Belgium (28 May 1940) also fell to the Germans.

26 May – 4 June 1940: The German army advanced over its western front, trapping large numbers of British and French troops against the British Channel, necessitating their evacuation from Dunkirk.

June 1940: The French military collapsed and occupation began over much of the country.

**Figure 29.**
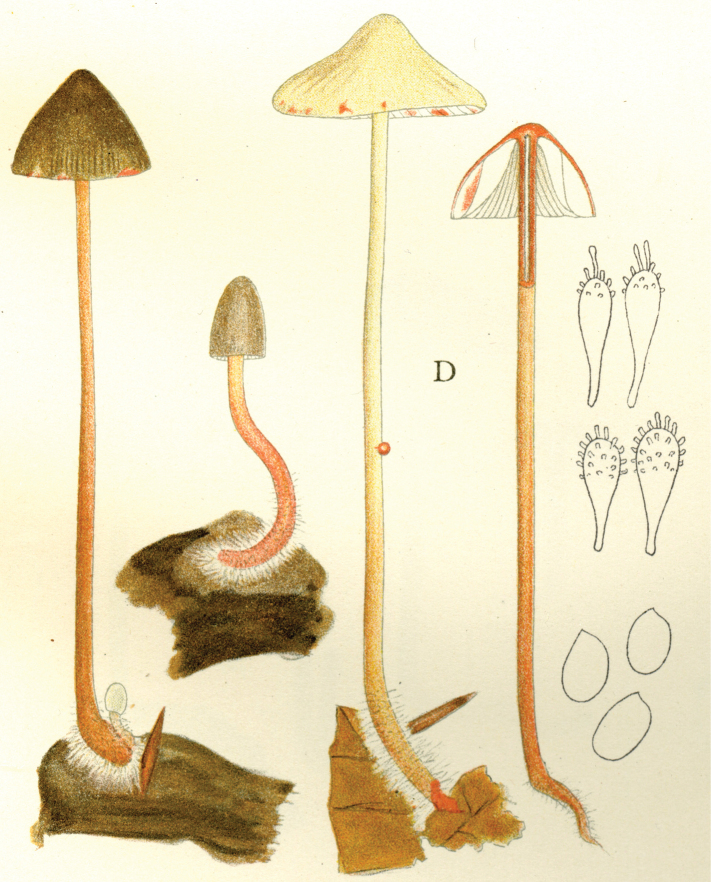
Detail from *Flora Agaricina Danica*. Plate 100D. *Mycenacrocata*. Original 9 × 11.5 cm.

**Figure 30.**
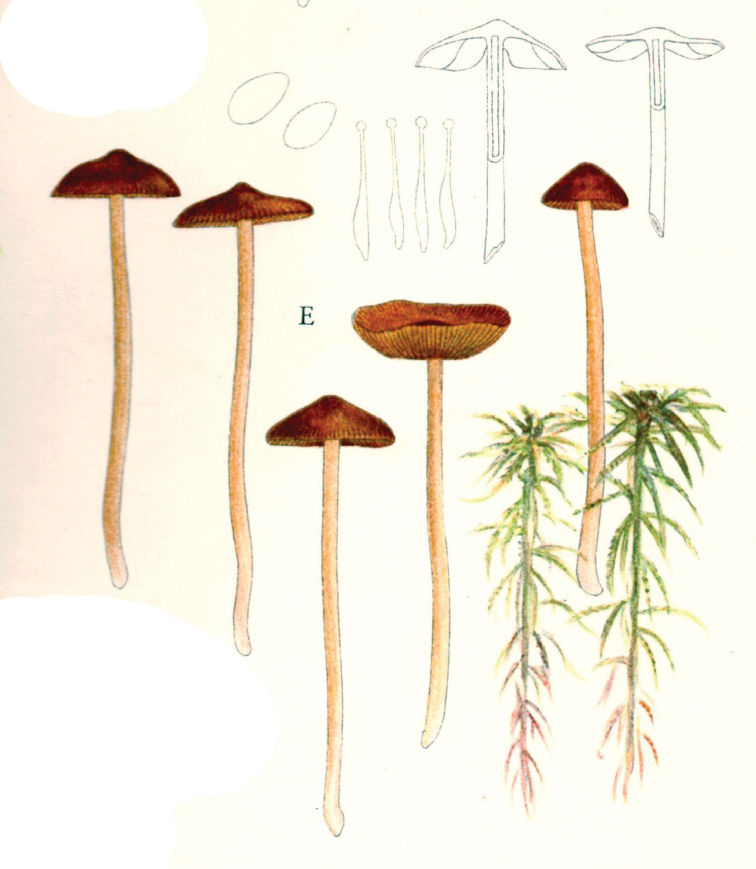
Detail from “Flora Agaricina Danica”. Plate 130E. *Galerasphagnorum*. Original 11.5 × 12 cm.

**Figure 31.**
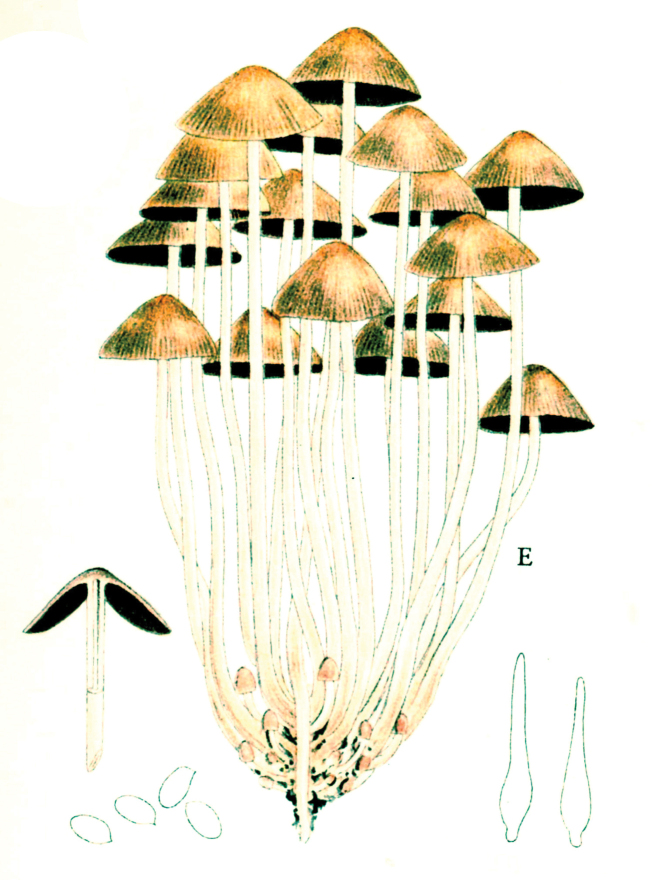
Detail from “Flora Agaricina Danica”. Plate 153E. *Psathyrastipitissima*.Original 9 × 12.5 cm.

**Figure 32.**
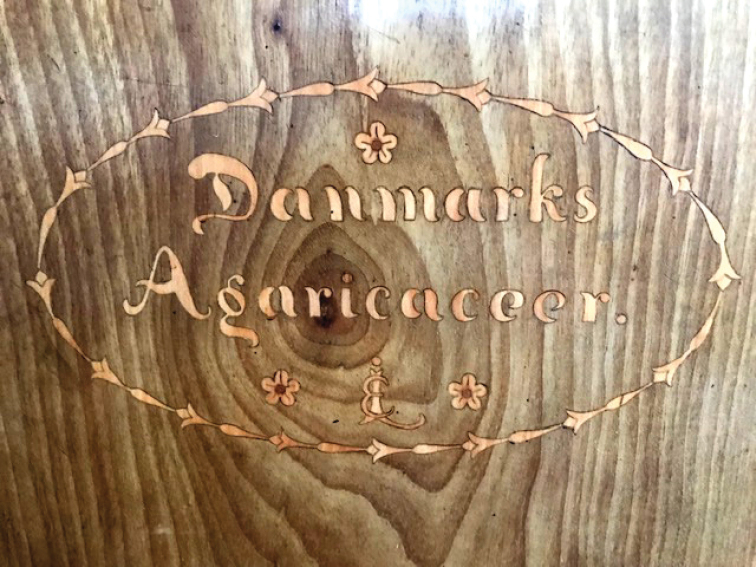
Detail of marquetry on the door of Jakob’s cabinet housing the first-run plates from “Flora Agaricina Danica”. Source: Lene Lange.

In mid-September, 1940, Volume V of FAD appeared (Figs 29–31). It had been planned as the final volume and was so. With long-term planning, it had been known that the number of plates would not reach the promised 200, so Jakob had augmented the illustrations in hand with several new ones, but without the generic organisation present in the preceding volumes. The final plates and descriptions were patched together in order to reach 200. Sometime during the production of the plates, Jakob designed and built a special cabinet to house a set of the original images. Even the door was customised with marquetry announcing “*Danmarks Agaricaceer*” (Danish mushrooms) (Fig. 32).

22 June 1941: Under “Operation Barbarossa,” Germany started its invasion of the USSR, ripping asunder the Molotov-Ribbentrop Pact.

It was surely Germany’s most grievous over-extension and the beginning of the end for Axis powers (Germany and Italy).

On the very same day, German occupation authorities in Denmark demanded that Danish communists be arrested. The Danish government complied and, in the following days, the Danish police arrested over 300 communists. Many of these, including the three communist members of the Danish Parliament, were imprisoned, in violation of the Danish constitution. On 22 August, the Danish Parliament (without its communist members) passed the “Communist Law”, outlawing the Communist Party and communist activities, in another violation of the Danish constitution. In 1943, about half of the detainees were transferred to Stutthof (Stutthof 2021) concentration camp (near the former “free city” of Danzig), where several died.

On Sunday, 7 December 1941, the air force of the Imperial Empire of Japan bombed the United States’ naval base at Pearl Harbour, Hawaii, concurrent with a declaration of war. The following day, the United States responded with its own declaration. Under the terms of the Tripartite Treaty, Germany declared war on the United States and on 11 December, the United States declared war on the Axis powers. The storm clouds of the preceding decade had broken into in a major storm.

**Figure 33.**
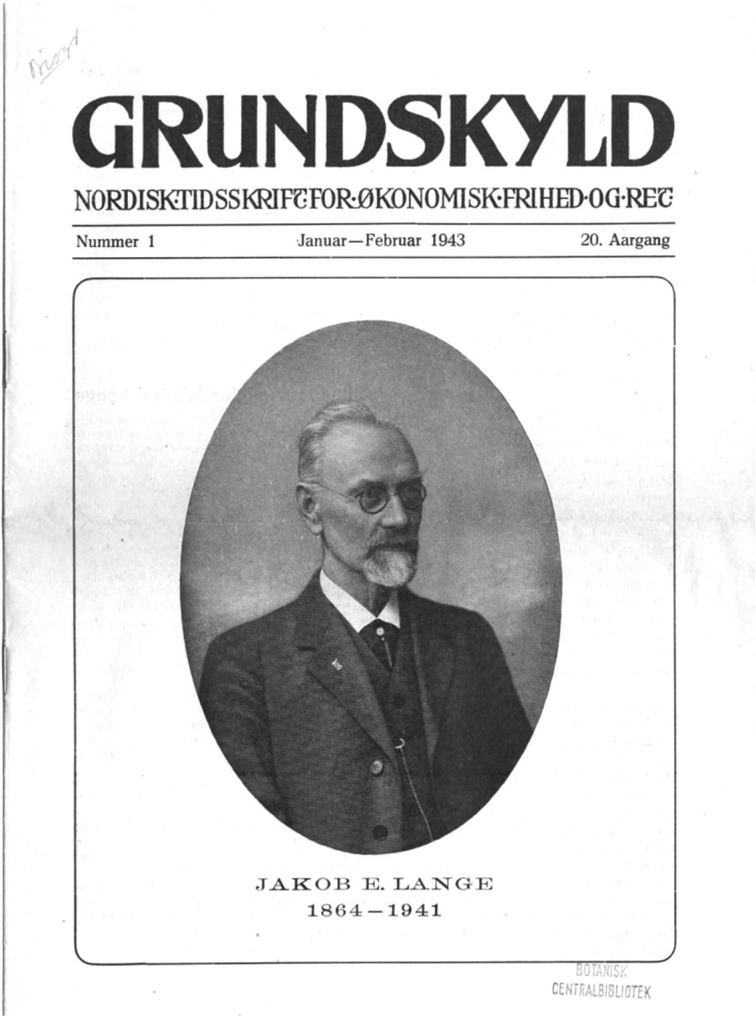
Jakob E. Lange on cover of “Grundskyld” (Nordic Journal of Economic Freedom and Justice). No. 1., 1943.

**Figure 34.**
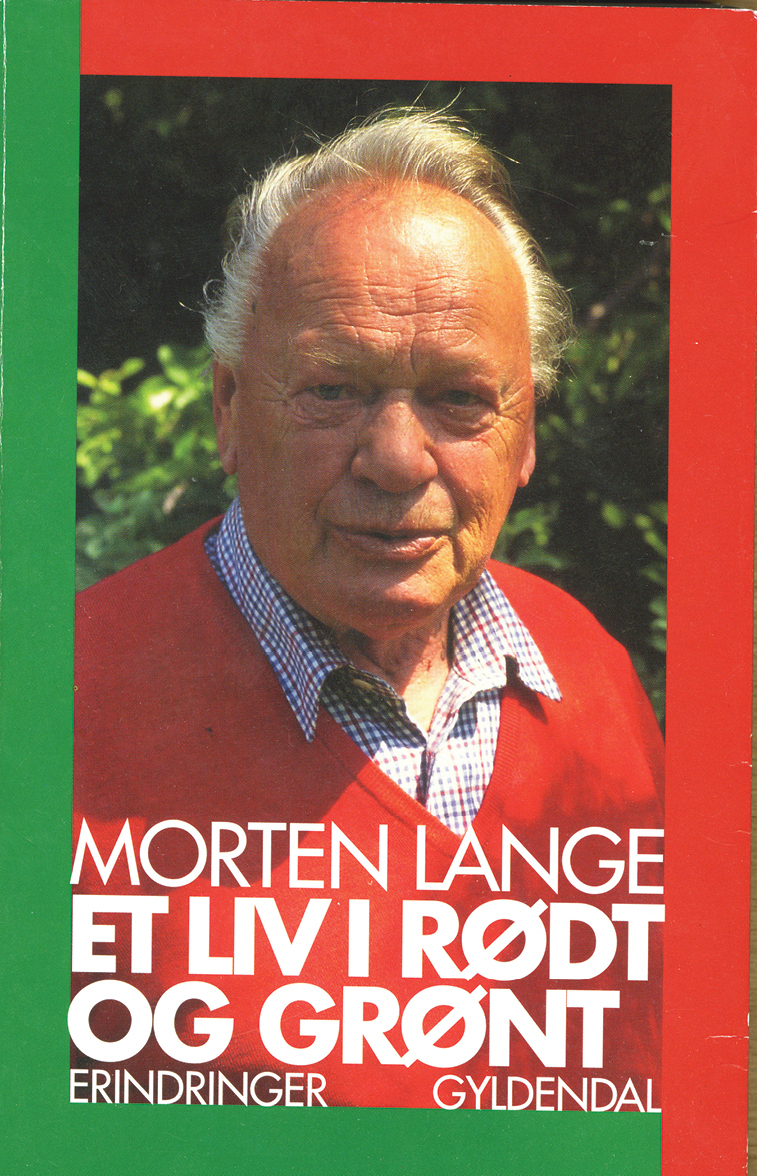
Knud Morten Lange in maturity. Source: on-line.

27 December 1941, Jakob Emanuel Lange (Fig. 33) breathed his last, just a few days after Christmas, although there might not have been much to celebrate. He was 77 years of age. His occupation had been in education, especially agricultural education and that reputation had underwritten his 1927 and 1939 trips to America. However, his preoccupation was mushrooms and especially their images (Lange 1943). This singular mindset had led to over 1000 such illustrations and their reproduction did not die with him. Morten (Fig. 34) used Jakob’s portraits in a popular English language mushroom guide (Lange and Hora 1963, with several printings), later in a Danish edition (Lange and Lange 1970), with translation editions in Dutch, Finnish, French, German, Italian, Norwegian, Spanish and Swedish. Eventually, the original set of the portraits was deposited in the Botanical Museum in Copenhagen, now the Natural History Museum of Denmark, whereas the copy set is privately owned. Morten’s daughter, Lene, remains a mycologist, with interest in ecology and distribution (Lange 1974; Samson 2018).

In a eulogy, A.A. Pearson (1947) reviewed FAD’s five volumes as well as Jakob’s life and motivation.

Far from the European theatre, Japanese forces overran the Korean Peninsula, much of China and, in 1942, extended its holdings down the Indochinese Archipelago, including Singapore and much of the “Dutch East Indies”. At least two notable mycologists were swept up; E.J.H. Corner (1981) in Singapore and M.A. Donk (Maas Geesteranus 1973) in Buitenzorg (later Bogor, Indonesia). They were treated quite differently, but survived to continue productive mycologica research.

29 August 1943: Germany placed Denmark under direct military occupation.

1943: German occupation authorities initiated the round-up of Danish Jews. Many Danes donned stars of David in hopes of confusing the military police. Many Jews were rescued and smuggled to neutral Sweden.

During those days, the German SS (‘Schutzstaffel’ or “Protective Echelon” for its origin as Hitler’s personal bodyguard), expropriated the Odense “Husmandsskole” as their main headquarters for Denmark. Morten (Lange 1996): “… [father] always loved freedom and hated Nazism. In the last years of the occupation, his old home, the Small Holders’ School, was taken over by the Gestapo and they made it the worst prison in the whole country”. In the waning days of the European campaign, the Royal Airforce of Britain bombed the place into the ground^[9]^.

Denmark was liberated by British troops from the south in May, 1945. Within days, the war in Europe was concluded after the armies of the United States and USSR met within Germany. Germany capitulated for the second time in the 20^th^ century. It took another year before Japan was forced to surrender after two atomic bombs were dropped on major cities.

## ﻿Chapter 7. After-thoughts

This paper is a tale of a small corner of turn-of-the-20^th^-century mycology. Almost the entire cast was born before 1900, generally from the close of the United States’ Civil War through the “Gay ‘90s” and most lived into the decades of World War II. The cast saw economic feast and famine, repeated wars and the growth of democratic societies. It is told, for the most part, through the eyes of a non-American, a Danish man whose long working hours and imagination were occupied by cultural equality for his fellow citizens, but whose hobby was mushrooms, their forms and habitats.

In a recent Copenhagen newspaper, Politiken (8 July 2021), under the by-line, Gretelise Holm, there appeared a “chronology” about the small holders. The headline: “Small-holders are extinct, and soon we, children of the small-holders, will be gone too”. A summary follows. From 1880–90, ca. 140,000 men working in agriculture went to the cities to work there and 300,000 Danes emigrated, most to the United States. In 1899, a law established that small-holders’ farms could not exceed 3.5–5 “tønder land” (= 4.8–6.8 acres) to ensure that the small-holders would still be available as a working force for the larger farmers[!]. Later, this was changed so they could have 6–10 “tønder” (= 8–13 acres), enough to sustain an independent family. Seventy-five years ago (1946, just after World War II), the small holders’ movement had 115,636 members organised in 1317 local chapters. Between 1950 and 70, 50,000 small-holders disappeared. No matter how poor they were, the small-holders were not socialists, since they had “their own” place, although they often had to work for others as well in order to make a living. In 1993 the “Small-holders Society” changed its name to “Danish Family-farmers” and 10 years later, they fused with the larger farmers’ society and took the common name “Danish Agriculture”. The small-holders movement had withered and died.

Finally, the reporter discussed the pros and cons for the small farmers versus the large farmers. Some of the small farmers are now organised in the “Free Farmers” organisation, the homepage of which includes: “Problems with environment, biodiversity, climate, energy, distribution of the population, unemployment as well as physical and psychological health can be solved through a radical new structure, where the right to own land is spread to more hands.”

Jakob Lange had absolute confidence in his “portraits” as substitutes for long and, for him, tedious descriptions. His aquarelles, though, could not capture the variability of the taxon, but only the form and colour of the basidiomata of the single collection used as models. Colours of basidiomata were considered “true,” so his inclusion of a colour chart at the end of “Studies” part VI (on *Russula*; Lange 1926) is unexplained. Upon even rapid examination, it seems clearly done with his watercolour palette and brushes and probably added to over time, for the colours do not fit any pattern. A footnote refers to 970 completed portraits (in 1926) at the library of the Botanical Museum of the University in Copenhagen, referred to by Lange as “*Danmarks Agaricaceer*”. By 1926, American agaricologists were using Ridgway’s (1912; Petersen 2016) “Color Standards and Color Nomenclature”, while Europeans had Klincksieck and Valette (1908).

Not long before Lange’s “Studies” series commenced, came instalments of “North American Flora”, a contribution from The New York Botanical Garden, edited by and often written by William Alphonso Murrill (Murrill 1907,^1908^, ^1914^, ^1916^, 1917a, b; Murrill and Burlingham 1910; Burlingham et al. 1915; Murrill et al. 1924; Overholts and Kauffman 1932), some volumes of which dealt with the Agaricales. For Lange, they would have been a disappointment, for they were models of tedious descriptions in small print, with no illustrations. Keys were provided, but employed characters which were difficult to interpret. Barnhart’s (Murrill and Barnhart 1916) bibliography was, however, exhaustive and, to this day, serves as a guide to the literature prior to 1920.

**Figure 35.**
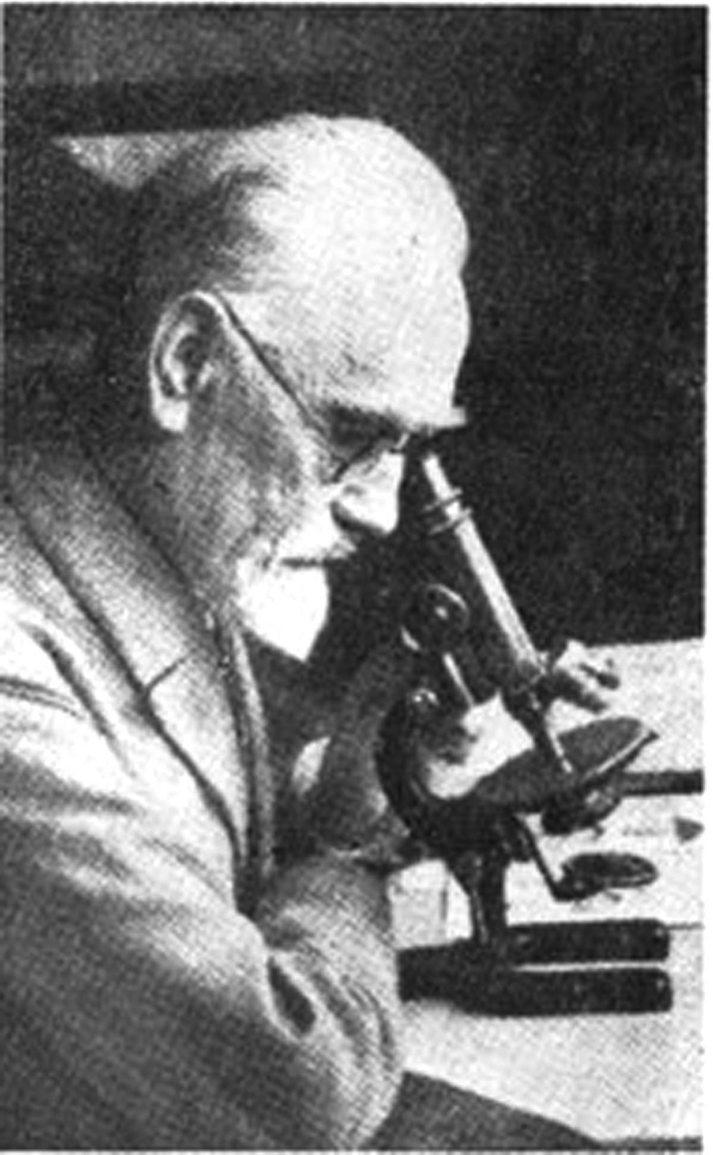
Jakob Lange at his microscope; age perhaps 60. Source: Henning Knudsen.

The meagre MycoPortal trail of Lange specimens are clues to his stops in 1931, reflective of his philosophy that illustrations were better than dried specimens. Noting his microscope (Fig. 35), though, gives clues to the quality of his observations of microscopic characters. Especially spore dimensions must be granted some leeway, much less cystidia. Lack of herbarium material forms an obstacle for molecular evidence of transatlantic congruence of taxa, precisely the motivation for his 1931 trip. Molecular analysis will help by facilitating proposal of epitypes. Lange’s conscious decision not to develop a herbarium of even the specimens he illustrated deprived future workers from accurately identifying and mapping the agarics of Europe (Heilmann-Clausen et al. 2019).

Lange’s admiration of Fries, who also did not keep a large herbarium, was explicit. However, Fries’ aquarelles were executed by hired professional artists, not Fries himself (Petersen and Knudsen 2015), even though he approved of each illustration. Fries’ plates may have served as models for Lange, for his uncle owned a copy of Fries’ illustrations.

Lange (1938b) related the intentions for his 1927 and 1931 trips, but we know that he did some mycological sleuthing in 1927 and some lecturing, probably on agricultural topics, in 1931. The latter trip could well have included stops purely for agricultural or educational appearances. The 1939 trip was explicitly devoted to both missions.

There is, of course, a temptation to examine Jakob Lange unidirectionally: by a mycologist as a mycologist, by a sociologist or economist as a social economist, by a politician as a politician. To do so would not do him justice. Lange was all of these, but at the bottom of it all, he was a Dane. The parting words of his memoir (Lange 1938b) were: “But the society we dare to dream about, the country of equality, must be built on the foundation of the people. It has been a happiness for me to have lived in an era and in a country, where a part of this popular character has germinated, to be able – through my information work – to guard and nurse its growth”.

## ﻿End notes

**1. Henry George (1839–1897)**

Henry [NMI] George was a leading American economic and political thinker, but he was also the epitome of the “rags to riches” American story. Raised under a strict Episcopalian father, he revolted against the parochial school, which was replaced by a tutor. He adopted a set of beliefs he called “deistic humanitarianism,” probably not far from present-day Secular Humanism.

His formal education ceased when he was 14 and, at 15, he shipped out as a foremast boy climbing the shrouds of a ship bound for Australia and India. That was 1855 and three years later, he disembarked in San Francisco, essentially penniless, where he settled into a job as a typesetter, unromantic but steady. He married his wife Annie, an Irish Catholic, but their four children were mainly influenced by their father’s deism and humanism.

His experience overseas and at home sowed embryonic seeds of economic thought. How could so many people exist in poverty at the same time that society seemed to become more prosperous? America seemed to magnify this incongruity. In a trip to New York City, he was amazed at the apparent paradox that the poor in that complex city of immigrants were much worse off than the poor in less developed California. He developed a philosophical concept questioning this *status quo* and began to write his thoughts: people should own the value of the product of their personal labour, but the land itself (including natural resources) should belong equally to all members of society. He argued that a single tax on land would create a more productive and just society. The more sophisticated and profitable developers of the land should pay more taxes; farmers should pay less. His words and ideas proved attractive to a wide spectrum of society and his influence grew apace. As might be expected, his popularity was chiefly in the working classes. His thoughts matured and. at age 40. he published a book, “Progress and Poverty” (George 1879), which proved to be his *magnum opus* and which eventually sold over three million copies worldwide. The philosophy became known as “Georgism” and spawned several reform movements during the Progressive Era.

One of his most enthusiastic followers was Jakob Lange in far-off Denmark. Lange would translate George’s books into Danish and expound on George’s philosophy in his teaching, first at Dalum, then at Odense. He integrated George’s principles into an overall context of social equality and education for the “small-holders.”

George became widely travelled after his publications’ successes, including a speaking tour of Great Britain. In his youthful zeal over Georgistic principles, Lange sought to travel to Britain to hear and meet George. Alas, there were no available passenger ships between Denmark and England to match his aspiration and time and he wound up sharing a boat with 600 pigs in order to attend George’s lectures. However, as luck would have it, Lange appeared just after George had spoken and departed for his next appointment. Lange bolted after him and after a couple more missed opportunities, did, indeed, meet and talk with Henry George. Lange was only 33 when George died.

Lange’s 1927 trip to the United States was due, in part, to his experiences and application of Georgist and Grundtvigian principles to Danish agricultural education. Lange came to know and befriend George’s daughter, Anne George de Mille and met several influential American followers of George’s philosophy.

**2. Olive Dame Campbell (1882**–**1954)**

Olive Dame Campbell was born to Lorin Low and Isabel (Arnold) Dame in Medford, Massachusetts. She married John C. Campbell in 1907 and moved to the mountains of north Georgia, where John was President of Piedmont College. From 1908 to 1912, she accompanied him through the mountains of Tennessee, Kentucky, Georgia, West Virginia and North Carolina, surveying social and economic conditions. After 1913, she established residence in Asheville, where John had been appointed Chief Executive Officer of the Southern Highlands Division of the Russell Sage Foundation. At his death in 1919, he had written only the preface and first chapter of a projected study of the Appalachian Region; she completed the manuscript, which was published under her husband’s name as “The Southern Highlander and His Homeland” in 1921 (Campbell 1921).

After John’s death, Mrs. Campbell became executive secretary of the Conference of Southern Mountain Workers, which he had founded; she held the position until 1928. Concurrently, in 1921, she joined the staff of the Russell Sage Foundation. In 1922, her interest in using traditional folk culture as a basis for social reconstruction led her to accept a Scandinavian-American Foundation fellowship for an eighteen-month study of adult education in Scandinavian folk schools. Based on her experiences, she wrote “The Danish Folkschool” (Campbell 1928). In 1928–29, she was instrumental in establishing the Southern Highlands Handicraft Guild, a marketing organisation for mountain craftsmen that still maintains its headquarters in Asheville.

It was during her Scandinavian study-tour that she met Jakob Lange, Headmaster of the *Fyn Husmandsskole* in Odense, Denmark. She interacted with Lange frequently and, in the preface to her book (Campbell 1928), she wrote: “I wish to acknowledge my deep indebtedness to the American Scandinavian Foundation which made this study possible … and especially to Jakob Lange, principal of the Smallholder’s School in Odense”. Thus was formed a close relationship between Denmark and the United States with the cause of social equality at its core. In late 1925, Olive returned to North Carolina and established the John C. Campbell Folk School in Brasstown, Cherokee County and, in 1927, Jakob was invited to join in the celebration of the new endeavour.

In the forty years that Olive Dame Campbell lived in western North Carolina, she collected folk songs, tallied mountain schools, studied Scandinavian education, established a school, organised numerous agricultural cooperatives, wrote books, conceptualised a craft marketing guild and organised multiple conferences (Campbell and Butler 1924; Campbell 1929). While some of her accomplishments are well recognised, others have gone unnoticed because of Campbell’s deference to others. During her lifetime, she wrote three books: one under her own name, one as co-author and one under her husband’s name. She also wrote his biography, kept a journal of their travels and, independently, wrote numerous articles and speeches (Fariello 2015).

**3. John C. Campbell (1867–1914)**

In the United States, John Charles Campbell (1867–1919), a product of Williams College (B.A. 1892) and Andover University (D.D. 1895), applied his experience to education in rural settings in Alabama and the Southern Appalachian Mountains. In 1901, he was appointed as superintendent of Piedmont Collegiate Institution, in Demerest, Georgia, tucked in the foothills of Georgia’s share of the Appalachian Mountain chain. The institution was new, having been founded in 1897 and served a rural population, with an enrolment of less than 400 from 1^st^ grade to second year of college. Within a short time, Campbell was promoted to Dean and eventually to President of Piedmont. Widowered during his tenure at Piedmont, on a recuperation vacation trip to Scotland, he met and, in 1907, married Olive Arnold Dame, 15 years his junior, who shared John’s deep concern about the people of “Southern Appalachia”. Over more than the next decade, they attempted to determine social needs and their remedies within the Appalachian region. They set out trekking the wilderness and back roads from north Georgia to West Virginia. On occasion, they were able to travel by rail; at other times the remoteness of their destinations required they walk into deep hollows and isolated “coves” to make contact with the sequestered inhabitants. The Campbells became amongst the first to distinguish and map Southern Appalachia as a distinct region. John popularised the name Southern Highlands. In 1913, the Campbells established residence in Asheville, North Carolina, in the heart of the southern Appalachians. Across the mountains in Tennessee, Horace Kephart (1862–1931; Kephart 1913) was publishing a very different view of Southern Highland life.

The Campbells shared the same idealism and were determined to provide aid, through a combination of education and humanitarian endeavours and, as their experience grew, they heard about a far-off land where an older tradition was addressing the education and well-being of “small holders”–the Scandinavian Folk High-schools.

Upon the early death of John in 1919, Olive continued their studies. John had intended to publish a book based on their work and she finished that task (Campbell 1921).

**4. Jakob Lange’s impressions of America (Lange 1931; translation, HK).**

Once on the road away from New York in 1931, Lange found himself in rural America. All around him were forests and farms comparable to those of Europe, but with exceptional differences. These differences, he concluded: “... are also found in the human world. There are, of course, European elements, especially Irish, but also Scots, English, Scandinavian etc. – not to speak of the ’coloured,’ but next to that, often distinctly felt, is something American. The American ’farmer’ is not just straightforwardly a Danish peasant and does not have the imprint on him of a history of former suppression, of an underclass living, but also not of romantic overcapitalisation as ‘the peoples’ nucleus and strength’. But nonetheless, I was struck time after time, by how uniform Danish and American attitude could be: a certain natural feeling of equality, a certain ‘well-grounded’ feeling we met everywhere. No snobbery, but straightforward helpfulness is probably the most characterising feature which has come to be developed in the internationally mixed colonies, where ‘what can I do for you’ is the first a newcomer will hear” (Lange 1931).

“The president, the director of the Agricultural high school, Dr Butterfield, had not only a winning personality, but was an important personality of real dimensions, with radical thoughts – which, by the way, cost him his position at the high school a short time after, under pressure from reactionary capitalistic influence, felt here [Denmark] as so often in America.

“But in America you should be able to overcome a surprise, as my wife – totally unprepared – had to, when they asked her to take the podium at a meeting where I had spoken (which she proudly did, so the acclamation was practically larger and at least more heartfelt than after my lecture)”.

Some remarks on the architecture in Washington, D.C. The capital building’s “capitolium with its huge cupula makes a distinct impression – pure in the lines and powerful in the dimensions – especially when you have not seen the many copies, in smaller format, around the capitals of the individual states (e.g. in Maine and Michigan). But by the way there is a strong prominent tendency in American architecture (when something monumental is sought for) to follow classical, Greek-Roman role-models, especially when you so-to-say build in supernatural size: huge Parthenons, in snow white, polished marble, seems a little far from Greek. In opposition to this is ‘the White House,’ the President’s home, which makes an impression on you by its modest noblesse, almost as in Washington’s own house at his mansion, Mount Vernon, not far from the capital, now a common place to visit for people from all States, a kind of American Mecca.

“It is instinctively repulsive as soon as you get south of Washington, to see the distinct barrier between ‘white’ and ‘coloured’ being retained everywhere. [Lack of capitalised references to race is his.] In the waiting rooms along the railway, there is a barrier: white at one side, black at the other; the two sides even have their own ticket-hole. A ‘coloured’ – no matter how weak the negro-features may be, cannot enter a ‘white’ restaurant. Equalizing measures, e.g. common schools, have time after time been abandoned and the underclass look is everywhere connected to the negroes. But you understand anyway better having seen it yourself, why the deep ditch is so difficult to fill”.

**5. Henry Agard Wallace (1888–1965)**

An Iowan, Henry Agard Wallace’s father, Henry C. Wallace, served as Secretary of Agriculture under Warren G. Harding, popular Republican President, so national service was not strange to Henry A. Once graduated from Iowa State University (1910), he joined the family weekly publication, “Wallace’s Farmer” as a writer and soon thereafter, as editor. As a professional farmer, he also founded the Hi-Bred Corn Company (Hi-bred 2021), which eventually became extremely successful. Over subsequent years, Wallace delved into numerous pursuits, from statistics and economics to religious and spiritual movements, including Georgism. After his father’s death in 1924, Henry drifted away from the Republican Party and supported Franklin Roosevelt in the 1932 election campaign. Wallace’s experience with poverty-stricken, southern, tenant farmers resonated with the Danish history of peasant culture. By American standards, Wallace supported a monumental governmental role in agriculture in harmony with Roosevelt’s New Deal policies, a view not without backlash in Congress and the U.S. Supreme Court.

During the War years, Roosevelt sent Wallace, by then Vice President, to the Soviet Union (now Russia) to report on the “collective agriculture” programmes. Unbeknownst to Wallace, he was duped by Soviet authorities and returned with a glowing report, which fed into accusations of being a Communist (with capital C), a label which became anathema as US/Soviet relationships soured. As a result, Wallace was dropped from the ticket for the 1944 election in favour of Harry S. Truman.

Jakob Lange would have found common ground with Wallace had the two met at the Williams College colloquium in 1927 or Lange’s possible lecture in Washington in 1939.

**6. Berea College (1855–present)**

The founding and development of Berea College mirrors both the ebbs and flows of United States history, as well as bearing a close resemblance to the aspirations of the “Small-holders High-schools” of Denmark.

Not far from Lexington, Kentucky, the Rev. John G. Fee started a one-room school in 1855 that eventually would become Berea College (Berea 2021). Fee envisioned a school that would be an advocate of equality and excellence in education for men and women of all races. Although the emancipation decree was not signed until 1865, Fee’s uncompromising faith and courage in preaching against slavery attracted the attention of Cassius M. Clay, a well-to-do Kentucky landowner and prominent leader in the movement for gradual emancipation. Clay offered Fee a 10-acre homestead on the edge of the mountains if Fee would take up permanent residence there. Fee accepted and established an anti-slavery church with 13 members on a ridge they named “Berea” after the biblical town whose populace was open-minded and receptive to the gospel (Acts 17:10).

Berea’s first teachers were recruited from Oberlin College, an anti-slavery stronghold in Ohio. Fee saw his humble church-school as the beginning of a sister institution “which would be to Kentucky what Oberlin is to Ohio, anti-slavery, anti-caste, anti-rum, anti-sin”.

The first articles of incorporation for Berea College were adopted in 1859, the year of Darwin’s “Origin of Species”. However, it also was the year Fee and the Berea teachers were driven from Madison County by southern, pro-slavery sympathisers. Fee spent the Civil War years raising funds for the school; in 1865, following Lincoln’s emancipation proclamation, he and his followers returned. A year later, the articles of incorporation were recorded and, in 1869, the college department became a reality.

Berea’s commitment to interracial education was overturned in 1904 by the Kentucky Legislature’s passage of the Day Law, which prohibited education of black and white students together. When the U.S. Supreme Court upheld the Day Law, Berea set aside funds to assist in the establishment of Lincoln Institute, a school located near Louisville, for black students. When the Kentucky Day Law was amended in 1950 to allow integration above the high school level, Berea was the first college in Kentucky to reopen its doors to black students.

By 1911, the number of students seeking admission to Berea was so great that the trustees amended the College’s constitution to specify the southern mountain region as Berea’s special field of service. The commitment to Appalachia, however, had begun as early as 1858, when the region was identified as a “neglected part of the country”.

In the early 1920s, in addition to its College Department, Berea established a high school that included ungraded classes for students who had not had educational opportunities, an elementary school, a vocational school and a Normal School for teacher training. No student was charged tuition. Most students worked for the College, whether weaving on the loom or milking in the dairy.

**7. Louis F. Post (1849–1928)**

Louis Freeland Post was a controversial Assistant Secretary of Labor in the Woodrow Wilson administration. His collegial friendship with Jakob Lange was based on his (Post) strong and vocal approval of the political ideas of Henry George, especially his uniform tax plan, but it was his stance against anti-immigration laws, which put him at odds with powerful politicians of the day.

Deportation laws were being stringently applied when the temporary absence of the Secretary of Labor and that of the Chief Assistant coincided and left Post in charge of the Department. Post considered the operative deportation process to be a “witch hunt” and he ordered a complete review of recent deportations, dismissing 71% of them as unconstitutional. This, not unexpectedly, brought him afoul of A. Mitchell Palmer, Attorney General and J. Edgar Hoover, Head of the Justice Department’s “Radical Division”, an organisation committed to rooting out foreigners with “radical” intentions, and the precursor to the Federal Bureau of Investigation (FBI). As early as January 1920, Hoover’s FBI began compiling a file on Post and his political leanings, but failed to find substantive evidence of radical connections on his part. A call for Post’s impeachment followed, but his defence of his actions won the day and impeachment was withdrawn.

An evening with the Langes must have provided some hearty conversation on Georgist ideas and the progress of his movement on both sides of the Atlantic.

**8. Mary Whetstone (1845–1929)**

Dr Mary J.S. Whetstone, Corresponding Secretary of the Minnesota Mycological Society, died at the age of 84 years (Davis and Seaver 1930), just two years before the Langes passed through. As Mary J. Snoddy, she received the degree of M.D. from the University of Michigan in 1881. She attended clinics in London, Paris, Vienna, Detroit, Boston and New York, serving on the staff of New England Hospital for Women and Children, Boston. She promoted the Northwestern Hospital for Women and Children, was an active worker for suffrage as a member of the Women’s Welfare League and, in addition to her practice, was interested for many years in the study of fungi (Dillon 1924). She was a premature widow. Her husband, Allen S. Whetstone, also a physician (Univ. of Mich., M.D. 1880), died in 1909. Like so many other collectors across the country, she was a correspondent of Charles Horton Peck and Curtis Gates Lloyd.

In her first letter to Peck (3 November 1898), she introduced him to the Minnesota Mycological Club, then a group of “about 20 enthusiastic, intelligent members”. She inquired whether he would be willing to help “with difficult identifications”. Peck obliged, as he did repeatedly with scores of such collectors and a correspondence commenced, lasting for at least a decade. Descriptions accompanied dried specimens and Peck did his best to provide pertinent information.

However, just as collegial as Whetstone would have been, there was also a prodigious number of Scandinavian settlers in the neighbourhood. The Langes would have been quite at home in the field with transplanted Scandinavians.

**9. The fate of the Odense Husmandsskole.**

(Husmandsskole 2021a, b). The Gestapo established three main headquarters in Denmark: in Copenhagen, at Århus and on Fyn (*Fyns Stifts Landbrugsskole*). The first two were bombed by the British Royal Air Force (RAF), so the Gestapo on Fyn also expected to be bombed. For this reason, they placed huge camouflage nets over the school and surrounding buildings, so when the RAF came with six Mustangs with 225 kg bombs, they could not find the target in spite of the maps the pilots had received from the Danish resistance, who asked for the bombing! They had to fly three times over the target before they finally let the bombs fall. The Gestapo, however, had been warned by telephone from Sild-Rømø, where the Mustangs entered Danish air territory flying only 15 m above the sea, so as not to be detected by the German radar. However, they were discovered and when they arrived at the school, the Gestapo had left and the sirens only left nine minutes for people to escape before the bombs fell. Due to the cover over the buildings, the bombs were not very precise and only nine local people died, just 16 days before the end of the war! The school was rebuilt not too far away.

In memory of Jakob Lange, a monument was built, like a shrine, where he is depicted surrounded by children and people and there is a poem in his honour.
